# Hydrogen Adsorption on Transition-Metal-Decorated Graphene: Thermodynamic and AIMD Insights from DFT and Post-Hartree–Fock Methods

**DOI:** 10.1021/acs.jpca.5c07741

**Published:** 2026-06-26

**Authors:** Wilmer Esteban Vallejo Narváez, Cesar Gabriel Vera de la Garza, Serguei Fomine

**Affiliations:** † Instituto de Investigaciones en Materiales, 7180 Universidad Nacional Autónoma de México, Apartado Postal 70-360, CU, Coyoacán, Ciudad de México, 04510, México

## Abstract

Hydrogen adsorption on transition-metal-decorated graphene was investigated using a finite graphene nanoflake model combined with revPBE-D4 geometry optimizations and ωB97M-V single-point energies, complemented by PNO–CCSD­(T) and DLPNO–CCSD­(T) benchmarks for representative systems. Particular attention was paid to the role of spin-state effects and finite-size electronic structure, which are often neglected in periodic models. Electronic-structure analysis reveals size-dependent open-shell character in graphene nanoflakes —one larger (G) and one smaller (G2)—where G exhibits robust, where G exhibits robust multiconfigurational singlet behavior and G2 a weaker, functional-dependent tendency. CASSCF/NEVPT2 calculations support this picture and corroborate the high-spin assignments of Sc–G and Y–G. Among the metals examined, Y, Nb, and Pd exhibit strong binding to graphene (−0.67 to −1.02 eV), whereas Sc, Zr, and Ni show moderate stabilization. Hydrogen adsorption is strongly system-dependent: the first H_2_ adsorption is exergonic for Y, Zr, Nb, Ni, and Pd (−0.56 to −1.63 eV), while Sc becomes favorable only upon adsorption of a second H_2_ molecule. In contrast, additional H_2_ uptake is generally disfavored for Y, Nb, and Ni, and only moderately favorable for Zr (−0.32 eV) in the second adsorption step. Three representative systems (Pd, Zr, and Sc), spanning distinct bonding regimes—molecular adsorption, dissociative hydride formation, and high-spin configurations—were used to assess the methodology, with ωB97M-V//revPBE-D4 showing close agreement with coupled-cluster interaction energies within these cases. AIMD simulations on Sc–G–2H_2_ and Pd–G–H_2_ reveal distinct finite-temperature responses: desorption of one H_2_ molecule and metal mobility in the Sc system versus persistent H_2_ coordination with bond elongation in the Pd case. Overall, the results show that hydrogen adsorption on TM-decorated graphene nanoflakes depends sensitively on the interplay between metal identity, spin state, and finite-size electronic structure.

## Graphical abstract

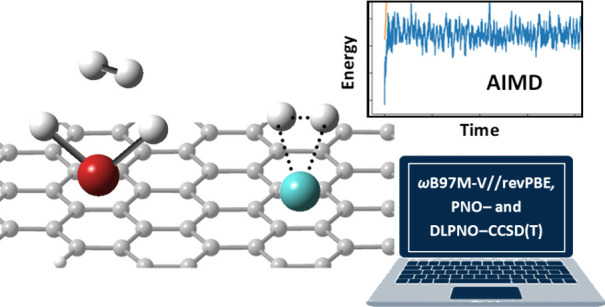

## 1 Introduction

Hydrogen is a clean, abundant, and high-energy-density fuel whose adoption in transportation and portable devices requires efficient and reversible solid-state storage media. Current liquid and compressed-gas technologies suffer from high cost, safety risks, and limited gravimetric capacity. The U.S. Department of Energy (DOE) recommends storage materials achieving ≥ 6.5 *wt*% H_2_ and adsorption energies within 0.2–0.7 eV per H_2_ to ensure reversibility under ambient conditions.^1–4^

Graphene and related 2D materials offer large surface area, low density, and chemical tunability, making them promising platforms for hydrogen storage through multiple adsorption modes, including physisorption, molecular adsorption on metal sites (end-on η^1^-H_2_ and Kubas-type side-on η^2^-H_2_ interactions), and dissociative adsorption leading to metal–hydride formation; in some cases, dissociation may be followed by H migration onto the graphene surface (spillover).^5–9^ However, pristine graphene interacts too weakly with H_2_ (≈ 0.05–0.1 eV), necessitating functionalization through defects, heteroatom doping, or decoration with electropositive metals. In particular, transition-metal (TM) decoration enhances hydrogen binding through *d*-orbital interactions and polarization effects, enabling adsorption modes ranging from physisorption to Kubas-type molecular binding and dissociative hydride formation.^10^ Experimental studies, especially on Pd–graphene systems, have demonstrated enhanced hydrogen uptake via spillover and defect-mediated mechanisms, highlighting the practical potential of these materials.^11,12^

Complementary DFT investigations have systematically examined TM decoration at the single-atom and cluster levels, showing that Pd_4_, Pd_3_P, and Ti/Pd codecorated graphene significantly improve adsorption performance due to strong orbital interactions and charge transfer.^13–17^ However, reported adsorption energies vary widely depending on the exchange–correlation functional and dispersion treatment, complicating quantitative comparisons and limiting predictive reliability.

In contrast, alkali and alkaline-earth metals, such as Li and Ca, have also been utilized to functionalize graphene, thereby enhancing its hydrogen storage capacity.^18–20^ Another promising strategy is dual- and codoping, where graphene is modified with combinations such as Li/Na, B/N, or Sc.^21–28^ These approaches have been shown to adjust hydrogen binding energies toward the desirable range while maintaining reversibility, a critical factor for practical use. In addition, defect engineering has emerged as a key design pathway. Structural modifications such as grain boundaries, heteroatom substitution, and vacancies dramatically influence adsorption energetics and facilitate hydrogen spillover, thereby improving overall storage capacity.^29–33^ Collectively, the experimental studies^7–11,18^ and theoretical investigations^13–19,21–28^ reveal the remarkable versatility of graphene-based materials and identify transition-metal–decorated graphene as a highly promising and adaptable platform for advancing next-generation hydrogen storage technologies.

Despite this, several DFT studies have explored individual TM–graphene systems but reported interaction energies vary widely with the choice of functional and dispersion correction, complicating quantitative comparison. The spread in these results arises from the different exchange–correlation functionals used—LDA-PWC, GGA-PBE-D2, DMol^3^-PBE-DNP, etc.—and the absence of consistent benchmarking.^13,14^ Moreover, no one systematically compares adsorption thermodynamics and dynamics across metals within a single high-accuracy framework.

Recent high-level theoretical studies have significantly advanced the understanding of hydrogen adsorption on metal-decorated graphene. In particular, diffusion Monte Carlo (DMC) calculations have provided highly accurate reference adsorption energies, demonstrating that commonly used density functional approximations can substantially overestimate binding strengths.^34^ In parallel, systematic density functional theory (DFT) investigations have shown that H_2_ adsorption on metal-decorated graphene can be classified into three distinct physical mechanisms: weak van der Waals physisorption, metal-induced polarization, and Kubas-type chemisorption involving back-donation into the H_2_ σ* orbital.^35^ While these works establish reliable energetic benchmarks and mechanistic frameworks, they primarily focus on periodic models and do not explicitly address the role of spin-state effects or finite-size electronic structure.

Despite these advances, several fundamental aspects remain insufficiently understood:

i First, most studies rely on periodic models of graphene and implicitly assume a well-defined electronic ground state, while the role of finite-size effects and edge-induced electronic structure in graphene nanoflakes has received comparatively little attention. In contrast, finite graphene nanoflakes converge adsorption energies to experimental values without periodic boundary conditions.^36^ This strategy is especially suitable for low H_2_ coverage and single-atom or defect environments, where periodic images can artificially bias adsorption.^37^ Tachikawa demonstrated the key role of finite-size and edge effects in alkali-metal-assisted H_2_ adsorption on graphene nanoflakes,^38^ and Xiang et al. reported realistic H_2_ uptake trends for Cr-decorated nanoflakes.^39^ Overall, nanoflakes combined with dispersion-corrected hybrid DFT provide a robust and efficient framework to probe fundamental hydrogen storage mechanisms in 2D materials.
ii Second, the impact of the spin state of the graphene substrate and metal–graphene complex on adsorption energetics has not been systematically addressed, despite the known propensity of low-dimensional carbon systems to exhibit open-shell or spin-polarized ground states.
iii Third, the variability of DFT predictions across different functionals and the lack of consistent methodological frameworks continue to hinder the establishment of reliable adsorption trends across different transition metals.

In this work, we address these gaps by explicitly linking spin-state physics, finite-size effects, and adsorption energetics in metal-decorated graphene systems. We adopt a nanoflake-based model combined with the revised PBE functional coupled with D4 dispersion correction scheme (revPBE-D4),^40^ which has been shown to provide an improved description of chemisorption of atoms and molecules on transition-metal surfaces compared to conventional GGA functionals. Additionally, single-point electronic energies were refined using the range-separated hybrid meta-GGA functional ωB97M to better capture local adsorption environments, charge-transfer effects, and electronic structure features that may not be adequately described by conventional GGA approaches.^41^ This strategy enables a more consistent and higher-level description of hydrogen adsorption in systems where localized interactions and spin polarization play a significant role. Comparative assessments of modern exchange–correlation functionals have shown that ωB97M-V provides a favorable balance between accuracy and computational cost for systems involving both main-group and transition-metal elements with the results comparable with those obtained with CCSD­(T) family of methods.^42^

To further assess the reliability of the computational approach, selected systems are benchmarked using post-Hartree–Fock methods, including PNO–CCSD­(T) and DLPNO–CCSD­(T), which provide near–chemical accuracy for relative energies while maintaining computational efficiency.^43–45^

Our results demonstrate that hydrogen adsorption energetics on TM-decorated graphene are strongly dependent on the spin state of the system, and that commonly assumed trends in metal binding strength can change qualitatively depending on whether the substrate exhibits closed-shell or open-shell character. Moreover, we show that finite graphene nanoflakes provide a physically meaningful framework to capture these effects, highlighting the critical role of edge-induced electronic structure and local charge redistribution.

Using this framework, we present a systematic analysis of selected transition metals across different rows as anchoring centers on graphene. In particular, we examine the adsorption of one and two H_2_ molecules on Sc-, Y-, Zr-, Nb-, and Pd-decorated graphene, and analyze the associated charge-transfer processes, spin-state effects, and dissociative adsorption pathways, complemented by *ab initio* molecular dynamics simulations.

## 2 Computational Methods

All calculations were performed using ORCA 6.0.1 program.^46^ First, we evaluate the performance of the revPBE-D4 functional in combination with the modified triple-ζ basis set def2-mTZVPP,^47^ which was specifically developed to achieve improved computational efficiency while preserving a high level of accuracy in the description of energies, molecular geometries, and noncovalent interactions. This assessment is carried out by comparing computed structural parameters with experimental bond distances for a hydride-bridged heterobimetallic zirconium–aluminum complex and the C–C bond length in graphene.^48,49^ In addition, the ωB97M-V//revPBE-D4 methodology was evaluated for its ability to reproduce experimental bond dissociation energies (BDEs) associated with H_2_ loss from Sc^+^(H_2_)_2_ and Ni^+^(H_2_)_4_ complexes.^50,51^ All these results are presented in Figure S1 and Table S1 in Supporting Information.

Subsequently, a graphene nanoflake comprising 92 atoms was employed as the substrate to investigate selected first-, second-, and third-row transition metals (TMs) as potential hydrogen storage materials. Geometry optimizations were carried out using the revPBE-D4 functional. All optimized structures correspond to true minima on the potential energy surface, as confirmed by the absence of imaginary (negative) vibrational frequencies. Single-point electronic energies were then computed for all systems at the ωB97M-V/def2-mTZVPP level of theory. This functional was selected due to its demonstrated reliability in describing noncovalent interactions and transition-metal complexes.^41^

Interaction energies between graphene and transition metals were computed as stepwise (sequential) H_2_ adsorption on a metal–graphene (TM–G) site. For the *n*-th addition of an H_2_ molecule (with *n* = 1, 2, 3, 4, ...), define the sequence:

$$TM-G-{\left(H_{2}\right)}_{n}+H_{2}\to TM-G-H_{2}$$ 1

The Gibbs free energy for hydrogen adsorption is defined as

2 $$\Delta G^{\left(n\right)}=G\left[TM-G-{\left(H_{2}\right)}_{n}\right]-G\left[TM-G-{\left(H_{2}\right)}_{n-1}\right]-G\left[H_{2}\right]$$

where *G*(H_2_) is the Gibbs free energy of H_2_ molecule, *G*[TM*–*G–(H_2_)_n_], is the Gibbs free energy of TM*–*G–(H_2_)_n_ complex and *G*[TM*–*G–(H_2_)_n‑1_] is the Gibbs free energy of the reactant complex.

The average adsorption energy, Δ*E*, for hydrogen adsorption, which is commonly reported in the literature, is

3 $$\Delta E^{\left(n\right)}=\frac{1}{n}\left[E\left[TM-G-{\left(H_{2}\right)}_{n}\right]-E\left[TM-G\right]-E\left[nH_{2}\right]\right]$$

where *E*(H_2_) is the electronic energy of H_2_ molecule, *E*[TM*–*G–(H_2_)_n_], is the energy of TM–G*–*(H_2_)_n_ complex and *E*[TM*–*G] is the electronic energy of the TM–G complex. Hydrogen adsorption was examined by sequentially adding H_2_ molecules, followed by geometry optimization.

Thermochemical corrections and Gibbs free energies were computed at 298.15 K and 1 atm using ideal-gas translational/rotational contributions and harmonic vibrational frequencies (quasi-RRHO treatment; low-frequency modes treated as vibrations; no anharmonic corrections).

Moreover, self-consistent field (SCF) orbital stability analyses were performed for all systems to confirm that the obtained SCF solutions correspond to minima, rather than saddle points, with respect to orbital rotations.

The ground-state spin configurations of two graphene nanoflakes (G, 92 atoms; G2, 84 atoms) were evaluated using revPBE-D4, ωB97M-D4 and ωB97M-V//revPBE-D4 as shown in Table S2 (Supporting Information). The stability of the broken-symmetry solutions was verified through SCF stability analysis, and no lower-energy solutions were identified. The ⟨S^2^⟩ values were examined to assess the spin consistency of the wave functions. To examine whether the DFT spin-state ordering is affected by multiconfigurational character, selected graphene-based systems were analyzed using multireference calculations. Specifically, the Complete Active Space Self-Consistent Field method combined with second-order *N*-Electron Valence State Perturbation Theory (CASSCF/NEVPT2) was employed. For the pristine G nanoflake, the singlet and triplet states were computed at the CASSCF­(12,12)/NEVPT2/def2-mTZVPP level on revPBE-D4/def2-mTZVPP optimized geometries, and the natural orbital occupations of the CASSCF singlet wave function were inspected to assess the degree of open-shell and biradicaloid character.

For each TM-containing system, all relevant spin multiplicities were explored, and the energetically preferred spin state (i.e., the lowest in total electronic energy and Gibbs free energy) was used for the reported thermodynamic comparisons. The complete data set is provided in Table S3 in Supporting Information.

To further validate the spin-state assignments, the relative energies of different spin multiplicities were compared using both DFT and higher-level methods. For the Sc–G and Y–G complexes, doublet, quartet, and sextet states were evaluated at the CASSCF­(11,12)/NEVPT2/def2-mTZVPP level on revPBE-D4/def2-mTZVPP optimized geometries. A common CASSCF­(11,12) active space was retained for all multiplicities of a given complex; this space included the metal-centered *d*-orbitals and the graphene π-orbitals directly involved in the metal–surface interaction, enabling a consistent comparison of spin-state energetics (Tables S3 and S4 in Supporting Information). Together with the agreement with CASSCF/NEVPT2 results for representative Sc and Y systems, these findings support that the identified high-spin configurations correspond to physically meaningful electronic states within the present theoretical framework.

Benchmark single-point interaction energies were computed at the PNO–CCSD­(T) and DLPNO–CCSD­(T) levels on revPBE-D4-optimized geometries for three representative systems: Pd–G–H_2_, Zr–G–H_2_, and Sc–G–H_2_. These systems were selected to span distinct bonding regimes, including molecular adsorption, dissociative hydride formation, and high-spin configurations, thereby providing a representative assessment of the methodology across chemically diverse scenarios.

To assess the applicability of single-reference coupled-cluster methods, T_1_ diagnostic values were evaluated for these systems. The obtained values are below 0.05 (Table S5 in Supporting Information), indicating that the selected systems fall within a regime where single-reference approaches are expected to provide an acceptable description.

DLPNO–CCSD­(T) and PNO–CCSD­(T) calculations of the binding energies for the Pd–G–H_2_, Zr–G–H_2_, and Sc–G–H_2_ complexes were carried out using the ORCA 6.0.1 ^46^ and TURBOMOLE rev.V-7.9 program packages,^52^ respectively. The coupled-cluster calculations were performed with the def2-mTZVPP basis set. A standard TCutPNO threshold of 1 × 10^–7^ was employed in all DLPNO- and PNO–CCSD­(T) calculations.

NBO charges were calculated using TURBOMOLE rev. V7.9.^52^ NBO/NPA charges were obtained using the Natural Population Analysis (NPA) implementation in TURBOMOLE.

Ab initio molecular dynamics (AIMD) simulations were carried out using the ORCA package at the revPBE-D4/def2-mTZVPP level of theory. The simulations employed a time step of 1.0 fs with initial speeds generated at 300 and 500 K, and temperature control was achieved using a Nosé–Hoover chain (NHC) thermostat set to selected temperature with a relaxation time constant of 10 fs. Each trajectory was propagated for 14000 steps, corresponding to ∼ 14 ps of simulation time.

Periodic hybrid-DFT calculations were not performed because the large graphene supercells required here make exact-exchange evaluations computationally prohibitive under periodic boundary conditions. This limitation is further exacerbated when multiple geometries and AIMD sampling are required. Moreover, periodic calculations are not currently implemented for high quality CCSD­(T) benchmark calculations.

## 3 Results and Discussion

Before discussing the electronic structure and adsorption results, the computational protocol was validated against available experimental data. As shown in Figure S1 in Supporting Information, revPBE-D4 provides geometries in good agreement with experimental bond distances for a hydride-bridged zirconium–aluminum complex and for the C–C bond length in graphene. In addition, the ωB97M-V//revPBE-D4 approach reproduces the experimental bond dissociation energies (BDEs) for H_2_ loss from Sc^+^(H_2_)_2_ and Ni^+^(H_2_)_4_ complexes with good agreement (Table S1 in Supporting Information). These results support the use of revPBE-D4 for geometry optimization and ωB97M-V//revPBE-D4 for subsequent energy evaluation in the present study.

### 3.1 Electronic Structure of Graphene Nanoflakes

To assess the intrinsic electronic structure of the graphene support and its dependence on nanoflake size, the ground-state spin configuration of two graphene nanoflakes (G, 92 atoms; G2, 84 atoms) was systematically investigated across several levels of theory, including revPBE-D4, ωB97M-D4, ωB97M-V//revPBE-D4, and CASSCF­(12,12)/NEVPT2. All results are shown in Table S2 in Supporting Information.

For the larger nanoflake (G), all DFT methods consistently identify an open-shell electronic structure as the lowest-energy solution. In particular, the open-shell singlet state (OSS) is clearly preferred, lying 0.54 and 1.69 eV below the triplet and singlet states, respectively, as shown in Table 1. In particular, for G, the SCF stability analysis converged to an unrestricted solution, confirming that the open-shell singlet (OSS) state is more stable than the closed-shell solution in that case.

**Table 1:** Relative Energies (Δ*E*, in eV) for Different Spin States of Graphene Nanoflakes of Two Sizes (G, 92 Atoms; G2, 84 Atoms), Referenced to the Lowest-Energy State of Each System, Calculated at the ωB97M-V/def2-mTZVPP//revPBE-D4/def2-mTZVPP Level of Theory

System	Spin State	Δ*E* (eV)
G	OSS	0.00
	Triplet	0.54
	Singlet	1.69
G2	OSS	0.00
	Singlet	0.10
	Triplet	0.68

To further assess the electronic structure of the graphene nanoflake and the reliability of the spin-state assignments used throughout this work, selected systems were examined using CASSCF/NEVPT2 calculations (Table S2 in the Supporting Information). For the pristine G nanoflake, the CASSCF­(12,12)/NEVPT2 results identify the singlet as the lowest spin-adapted state, lying 0.19 eV below the triplet. These multireference calculations were designed to probe the frontier π-orbital manifold associated with the low-lying singlet–triplet electronic structure, rather than to provide an exhaustive multireference treatment of the full nanoflake π system. Importantly, the natural orbital occupation numbers of the singlet reference deviate markedly from the ideal closed-shell pattern. In particular, occupations of 1.3366 and 0.6650 indicate two strongly fractionally occupied orbitals, consistent with pronounced biradicaloid character. Additional fractional occupations, such as 1.7897 and 0.2122, further indicate that the electronic structure is not that of a simple two-orbital biradical, but rather that of a moderately multiconfigurational singlet. These multireference results are consistent with the DFT description, which favors a low-energy open-shell singlet solution over the triplet state for G. Nevertheless, the broken-symmetry DFT open-shell singlet and the spin-adapted CASSCF/NEVPT2 singlet are not formally equivalent; rather, the multireference treatment confirms that the low-energy singlet manifold possesses genuine open-shell/multiconfigurational character, consistent with previous reports on graphene nanoflakes.^53^

In contrast, for the smaller nanoflake G2, the energetic separation among the low-lying singlet solutions is substantially reduced and becomes more sensitive to the functional employed. At the ωB97M-V//revPBE-D4 level, the broken-symmetry OSS state lies only 0.10 eV below the restricted singlet, whereas the triplet state remains appreciably higher in energy (Table 1). This behavior suggests a reduced and less robust open-shell character relative to G, rather than a qualitative change in the preferred electronic configuration. Such sensitivity is consistent with previous studies showing that the polyradical character of graphene nanoflakes depends strongly on size, shape, and edge topology.^53^

This functional dependence is particularly evident for G2. At the revPBE-D4 level, the attempted OSS solution collapses to a spin-pure singlet (⟨*S* ^2^⟩ = 0.000), indicating that no distinct broken-symmetry solution is obtained. In contrast, ωB97M-D4 yields essentially degenerate restricted singlet and OSS solutions, while ωB97M-V//revPBE-D4 favors the OSS state by 0.10 eV. Taken together, these results indicate that the degree of open-shell character in G2 is less clearly defined at the DFT level than in the larger G nanoflake. This behavior is consistent with the opposite-sign spin-density pattern expected for an antiferromagnetically coupled broken-symmetry singlet description, as illustrated in Figure 1.^54^ In G the spin density is more delocalized across the graphene framework, whereas in G2 it is more localized, indicating a stronger confinement of the open-shell character. This size-dependent change in spin delocalization is consistent with previous theoretical studies of graphene nanostructures.^55^

**Figure 1:**
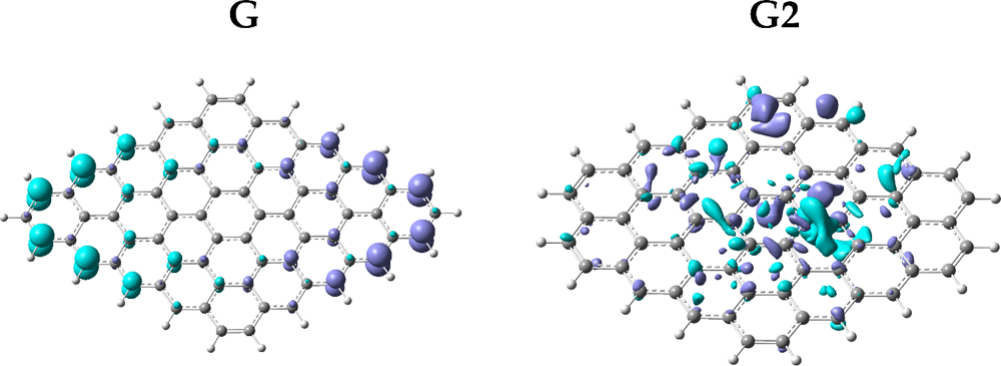
Spin density distribution for the G (left) and G2 (right) systems calculated at the ωB97M-V//revPBE-D4 level of theory. Blue and green isosurfaces represent positive and negative spin density, respectively.

These results highlight that the electronic structure of graphene nanoflakes depends sensitively on their size and shape. While G exhibits a robust open-shell, multiconfigurational singlet character, G2 displays a weaker and more functional-dependent tendency toward an open-shell description. The two nanoflakes therefore differ in the relative stability of their low-lying spin states and in the extent of spin delocalization, as shown by the spin-density distributions in Figure 1. This size-dependent electronic structure is relevant for interpreting metal binding and hydrogen adsorption trends, since the electronic character of the graphene support can modulate the local environment of the active site.

### 3.2 Metal–Graphene Interaction Energies

Based on the demonstrated reliability of the ωB97M-V//revPBE-D4 methodology for describing the structures and energetics of transition-metal systems, together with the electronic structure analysis of graphene nanoflakes, selected first-, second-, and third-row transition metals were further investigated (Figure 2).

**Figure 2:**
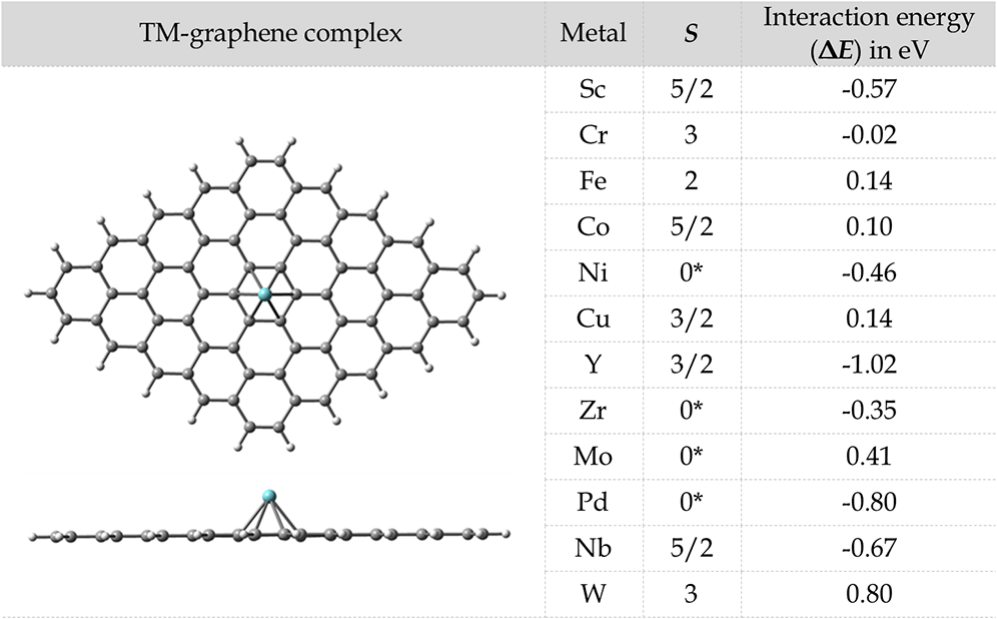
Left: Top and front views of the TM–graphene complexes. Right: Electronic formation energies (in eV) and total spin (*S*) values for the most stable spin states of the transition metal–graphene complexes. The label *S* = 0* denotes the unrestricted broken-symmetry (open-shell singlet) state.

Multireference calculations were also used to evaluate the spin-state assignments of selected metal-decorated graphene systems as shown in Table S4 in Supporting Information. At the CASSCF­(11,12)/NEVPT2 level, Sc–G exhibits a sextet ground state, with the doublet and quartet states lying 0.39 and 0.83 eV higher in energy, respectively. In contrast, Y–G favors a quartet ground state, while the doublet and sextet configurations are destabilized by 0.21 and 0.72 eV, respectively. Because the same active space was employed across all multiplicities of each complex, these results provide a balanced comparison of the competing spin states. The CASSCF/NEVPT2 spin-state ordering therefore corroborates the DFT high-spin assignments adopted for Sc–G and Y–G in the subsequent adsorption analysis.

Notably, Sc and Zr exhibit moderately negative Δ*E* values (−0.57 and – 0.35 eV, respectively), indicating favorable interactions of intermediate strength (Figure 2). Sc adopts a high-spin configuration (S = 5/2), whereas Zr stabilizes a lower-spin state (*S* = 0*), highlighting differences in their electronic structures. More generally, early transition metals tend to display moderately negative interaction energies, consistent with feasible adsorption onto graphene. These interactions may be associated with the presence of partially filled *d* orbitals that can interact with the graphene π-system. However, Sc complexes typically retain high-spin states (*S* ≥ 2), reflecting the weak ligand field generated by pristine graphene. High-spin states may enhance reactivity but reduce long-term electronic stability, a limitation also reported in prior studies on Sc- and Ti-doped graphene.^56^

Among the transition metals studied, Y (−1.02 eV, *S* = 3/2), Ni (−0.46 eV, *S* = 0*) and Nb (−0.67 eV, *S* = 5/2) exhibit negative interaction energies, indicating favorable binding to graphene. Their larger ionic radii and electropositive character of these metals facilitate donation to graphene, while their spin values remain relatively low compared to early 3*d* elements. This balance of strong binding and moderated spin multiplicity makes them promising candidates for catalytic or hydrogen storage applications, consistent with reports that Y- and Zr-decorated graphene exhibit enhanced hydrogen uptake and stability.^57^

In contrast, Cr, Fe, Co, Cu, Mo, and W do not exhibit favorable interaction energies, suggesting weak or unstable adsorption as shown in Figure 2. These elements show weaker stabilization, with some complexes displaying positive or near-zero interaction energies, indicative of endergonic or marginally stable binding. Their electronic configurations, half-filled or nearly filled *d*-shells—reduce the driving force for charge transfer, while simultaneously promoting high-spin configurations. For instance, Fe often remains in spin states above *S* = 2, in line with their free-atom multiplicities. This destabilization trend has been observed experimentally, where mid-row transition metals aggregate on graphene instead of forming uniform isolated sites.^56^

Finally, Pd (−0.80 eV, *S* = 0*) forms exergonic complexes with negative interaction energies and stabilizes in singlet states. Pd exhibits a closed-shell-like behavior (weak spin polarization), although *S* = 0* state corresponds to an unrestricted broken-symmetry solution. This behavior ensures robust thermodynamic and electronic stability, making them ideal for catalytic applications. Their role as active centers for hydrogen activation is well documented, and Pd- and Pt-decorated graphene have been experimentally validated as effective hydrogen dissociation platforms.^17^

### 3.3 Hydrogen Adsorption on TM–Graphene Complexes

Hydrogen adsorption was systematically analyzed for the most stable TM–graphene systems (Sc, Y, Zr, Nb, Ni and Pd). Figures 3 and 4 illustrates optimized geometries of the first and second H_2_ adsorption steps for Sc, Y, Zr, Nb, Ni and Tables 2 summarize the interaction free energies (Δ*G*), average electronic interaction energies (Δ*E*) for hydrogen adsorption as well as total spin states (*S*), including literature values for comparison. Importantly, this work provides both electronic interaction energies and Gibbs free interaction energies, allowing a more realistic assessment of thermodynamics at ambient conditions—data seldom reported in prior TM–graphene studies, which typically focus only on Δ*E* or binding energy per H_2_.

**Figure 3:**
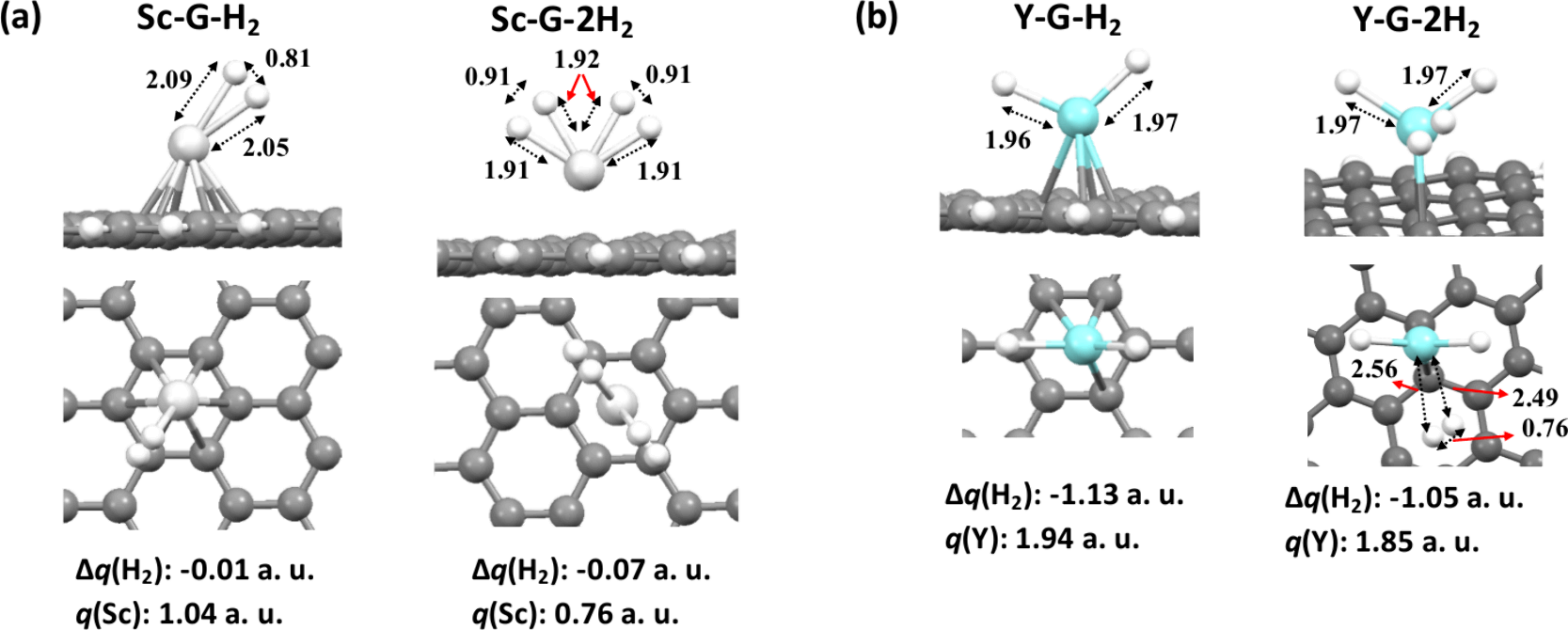
Optimized geometries of H_2_- and 2H_2_-complexes with graphene decorated with (a) Sc and (b) Y. The M−H and H−H bond distances (Å), together with the NBO charge transfer to H_2_ and the metal center, are indicated.

**Table 2:** Gibbs Free Interaction Energies (Δ*G*), Average Electronic Interaction Energies (Δ*E*), and Total Spin (*S*) for Hydrogen Adsorption on TM–Graphene Complexes Obtained in This Work, Compared to Representative Literature Values (ΔE*) Corresponding to Average Electronic Adsorption Energies Per H_2_ Molecule

System	Δ ***G*** (eV)	Δ ***E*** (eV)	***S***	Δ ***E*** * (eV) from literature	Literature Method	ref.
**Sc–G–H**_**2**_	0.06	–0.33	5/2	0.17–0.23	GGA-PBE, PAW, periodic	^58^
**Sc–G–2H**_**2**_	–0.29	–0.49	3/2	–0.56	B3LYP/6–31G(d,p) (nanoflake)	^59^
**Y–G–H**_**2**_	–1.09	–1.41	3/2	–0.67	PBE-GGA, periodic	^60^
**Y–G–2H**_**2**_	0.47	–0.74	1/2	–0.69	PBE-GGA, periodic	^60^
**Zr–G–H**_**2**_	–1.63	–2.00	0*	–0.66	PBE-D2, periodic slab	^61^
**Zr–G–2H**_**2**_	–0.32	–1.36	0*	—	—	—
**Pd–G–H**_**2**_	–0.62	–0.94	0*	–0.73	PBEPBE/LanL2DZ (nanoflake)	^62^
				–0.96	PW91/plane-wave, periodic	^63^
**Nb–G–H**_**2**_	–1.33	–1.63	5/2	–0.23	PBE-D2, periodic	^64^
**Nb–G–2H**_**2**_	0.23	–0.97	1/2	–0.39	PBE-D2, periodic	^64^
**Ni–G–H**_**2**_	–0.56	–1.14	0*	–1.21	PBE, periodic	^65^
**Ni–G–2H**_**2**_	0.26	–0.75	0*	–0.61	PBE, periodic	^65^

**Figure 4:**
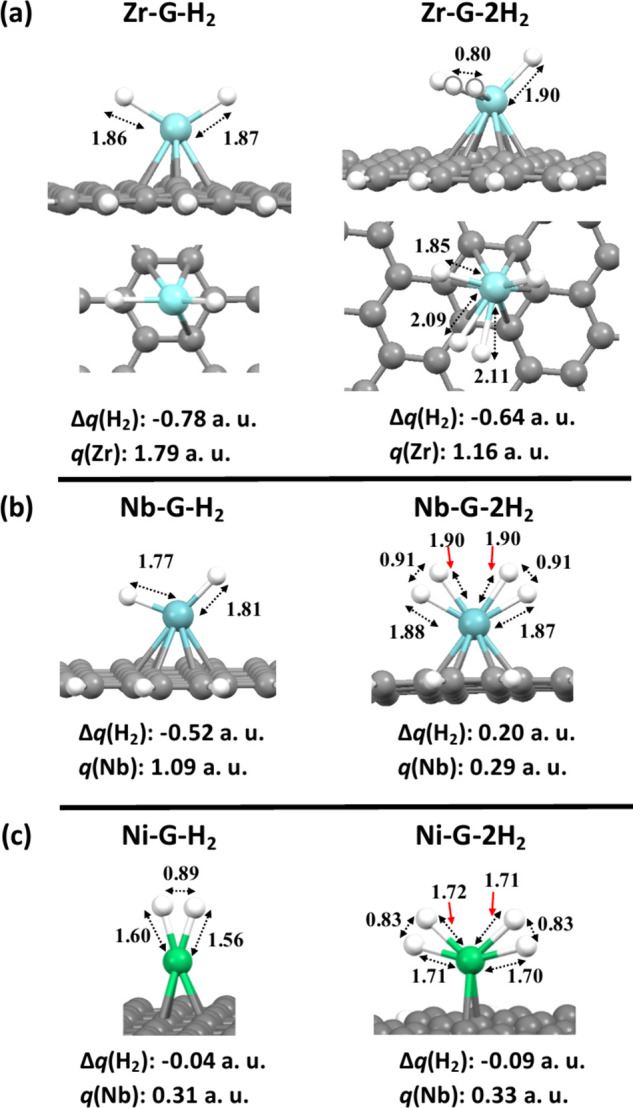
Optimized geometries of H_2_- and 2H_2_-complexes with graphene decorated with (a) Zr, (b) Nb, and (c) Ni. The bond distance of M-H and H–H (in Å) are indicated as well as the NBO charge transfer for some systems.

The binding energies of the Pd–H_2_–G, Zr–H_2_–G, and Sc–H_2_–G complexes were systematically evaluated at the ωB97M-V//revPBE-D4 and coupled-cluster levels (DLPNO–CCSD­(T)//revPBE-D4 or PNO–CCSD­(T)//revPBE-D4) using the def2-mTZVPP basis set, as summarized in Table 3.

**Table 3:** Calculated Binding Energies (in eV) for the Pd–H_2_–G, Zr–H_2_–G, and Sc–H_2_–G Complexes Obtained at the ωB97M-V//revPBE-D4 and DLPNO–CCSD­(T)//revPBE-D4 or PNO–CCSD­(T)//revPBE-D4 Levels of Theory Using the def2-mTZVPP Basis Set^a^

	ωB97M-V//revPBE-D4	CC//revPBE-D4	Error with respect to CC//revPBE-D4
System	eV (kcal/mol)		
Pd–H_2_-G, OSS	–0.94 (−21.60)	–0.94 (−21.65)^b^	0.00 (0.05)
Zr–H_2_-G, OSS	–2.00 (−46.09)	–2.06 (−47.50)^b^	0.06 (1.40)
Sc–H_2_-G, 5/2	–0.08 (−1.82)	–0.09 (−2.00)^c^	0.01 (0.18)

a CC represents DLPNO–CCSD­(T).

b CC represents PNO–CCSD­(T).

c Errors relative to CC//revPBE-D4 are also reported, with the most stable spin state indicated. Values in parentheses are given in kcal/mol.

For the Sc–G–H_2_ complex, the Sc–H distances (2.05–2.09 Å) and the short H–H distance (0.81 Å) indicate weak activation of the H_2_ molecule as shown in Figure 3(a). The negligible NBO charge transfer in the H_2_ molecule (Δ*q* = – 0.01 au) confirms limited electron donation to the σ* orbital, consistent with the positive Δ*G* value (0.06 eV) as indicated in Table 2. The high-spin configuration (*S* = 5/2) further destabilizes the interaction, in line with previous reports that high-spin early TMs display reduced electronic stabilization on graphene.^57^

The spin density distributions for the Sc–G and Sc–G–H_2_ systems (Figure S2) show that, in both cases, the spin density is primarily localized at the Sc center, confirming that the magnetic character is predominantly metal-driven. In the Sc–G system, partial delocalization over adjacent carbon atoms indicates interaction between the Sc *d*-orbitals and the graphene π-system. Upon H_2_ adsorption, the spin density becomes more localized around the metal center, reflecting a redistribution of the electronic structure induced by the adsorbate.

Upon adsorption of a second H_2_, Sc–H distances shorten to ∼ 1.91–1.92 Å and the H–H bond elongates to 0.91 Å, correlating with an increased charge transfer (Δ*q* = – 0.07 au) and a favorable free energy (Δ*G* = – 0.29 eV, *S* = 3/2) as shown in Table 2 and Figure 3(a). This suggests that Sc requires multiple H_2_ molecules to stabilize its coordination sphere. The calculated average Δ*E* values (−0.33 to – 0.49 eV) are consistent with previously reported theoretical data (0.17–0.23 eV and – 0.56 eV), indicating that ωB97M-V//revPBE-D4 captures the overall binding trend for Sc systems. These results are compatible with prior observations that interaction strength may increase upon adsorption of a second H_2_ molecule.

Despite the high-spin character of the Sc systems (*S* = 5/2), which was corroborated by CASSCF/NEVPT2 calculations, the T_1_ diagnostic remains below 0.05, providing no indication of severe single-reference breakdown (Table S5 in Supporting Information), indicating that they can be reliably described using single-determinant methods. This is further supported by the excellent agreement between ωB97M-V//revPBE-D4 and PNO–CCSD­(T) results, which yield nearly identical interaction energies (−0.08 and – 0.09 eV, respectively). These findings demonstrate that, for this high-spin system, the selected DFT method not only correctly predicts the ground spin state—consistent with NEVPT2—but also provides interaction energies in close quantitative agreement with CCSD­(T) methodologies.

Regarding Y–G–H_2_ complex, the results show a marked structural reorganization, where the H_2_ dissociates and hydride species is formed (Y–H = 1.96–1.97 Å) as presented in Figure 3(b). This agrees with the highly exergonic free energy (Δ*G* = – 1.09 eV) and a moderate spin state (*S* = 3/2) displayed in Table 2. Besides, the large charge transfer to the adsorbed H_2_ fragment (Δ*q*(H_2_) = – 1.13 au), together with the strongly positive metal charge (*q*(Y) = +1.94 au), indicates pronounced electron donation from the metal and strong activation of H_2_, consistent with a hydride-like/dissociative adsorption regime rather than a purely molecular (Kubas-type) interaction.

On the other hand, the second H_2_ adsorption destabilizes the system (Δ*G* = 0.47 eV) with elongated Y–H distances (2.49–2.56 Å), indicating partial activation and reduced stabilization (*S* = 1/2)-as shown inFigure [Fig fig3](b) and Table 2. The lowered spin (*S* = 1/2) reflects increased electron pairing but cannot compensate for the endergonic binding. For Y–G–2H_2_, the two adsorbed H_2_ molecules exhibit a net charge gain of Δ*q*(2H_2_) = – 1.05 au (≈ – 0.50 au per H_2_), while the H–H distances remain essentially molecular (*d*(H–H) ≈ 0.76 Å), indicating a strongly polarized yet nondissociative adsorption regime. The calculated Δ*E* values (−1.41 and – 0.74 eV) are more stabilizing than the literature (∼−0.67 to – 0.69 eV), reflecting the superior ability of ωB97M-V//revPBE-D4 to capture strong charge-transfer interactions in dissociative adsorption regimes compared to semilocal periodic functionals.

The Zr–G–H_2_ complex also leads to hydride formation, with short M–H distances (1.86–1.87 Å) and total dissociation of H_2_ as indicated in Figure 4(a). The large negative free energy (Δ*G* = – 1.63 eV) and open-shell singlet ground state (*S* = 0*) confirm strong binding and high electronic stability (Table 2). NBO analysis shows significant electron transfer to hydrogen (−0.78 au). For the second H_2_, mixed binding occurs: two hydrides remain short (Zr–H = 1.85–1.90 Å), while two weaker interactions form at Zr–H = 2.09–2.11 Å for the second hydrogen molecule (Figure 4(a)). The Δ*G* value becomes slightly negative (−0.32 eV, *S* = 0*), suggesting an attractive interaction for the second hydrogen molecule. Concerning the Δ*E* values (−2.00 and – 1.36 eV), they are noticeably more exergonic than those obtained with periodic PBE-D2 methods (≈−0.66 eV). This difference reflects the improved description of exchange and dispersion provided by ωB97M-V//revPBE-D4, as well as the ability of the nanoflake model to accurately capture local chemical environments and metal−π interactions. For Zr–G–H_2_ (*S* = 0*), RHF-based DLPNO–CCSD­(T) calculations were used as a correlated benchmark for the interaction energy. The *T*_1_ diagnostic, which remains below 0.05 (Table S5 in the Supporting Information), was considered only as an auxiliary indicator that severe single-reference breakdown is not evident for this system. All methods consistently predict strong binding (≈ – 2.00 to – 2.06 eV), demonstrating excellent agreement between ωB97M-V//revPBE-D4 and DLPNO–CCSD­(T) for the Zr complexes as shown in Table 3.

The possibility of storing more than two hydrogen molecules on Zr–G complexes was also examined. According to Table 4, the adsorption of the third and fourth H_2_ adsorption are endergonic (Δ*G* = 0.03 and 0.02 eV, respectively, indicating that further hydrogen uptake is thermodynamically unfavorable. The optimized structure of the Zr–G–3H_2_ and Zr–G–4H_2_ complexes are shown in Figure S3 in Supporting Information. This nonlinear adsorption behavior suggests a delicate interplay between steric hindrance and charge transfer effects. Similar behavior of Zr-decorated carbon frameworks has been reported for hydrogen storage.^66^

**Table 4:** Gibbs Free Interaction Energies (Δ*G*), Average Electronic Interaction Energies (Δ*E*) for the Third and Fourth Hydrogen Adsorption Process on Graphene Decorated with Zr, and the Total Spin (***S***) of the Most Stable State^a^

	Δ ***G***	Δ ***E***	
Species	eV		***S***
Zr-G-3H_2_	0.03	–1.01	2
Zr-G-4H_2_	0.02	–0.87	2

a The optimized structures are shown in Figure S3 in Supporting Information.

For Nb–G–H_2_, the first hydrogen molecule undergoes complete activation into hydrides, with short Nb–H distances (1.77–1.81 Å) as shown in Figure 4(b). The pronounced stabilization is reflected by the large negative free energy (Δ*G* = – 1.33 eV); however, the high spin state (*S* = 5/2), as reported in Table 2, points to reduced electronic stability despite the favorable thermodynamics. NBO charge transfer to hydrogen (−0.52 au) confirms substantial electron donation. The adsorption of a second H_2_ is unfavorable, with Δ*G* = 0.23 eV and no clear hydride formation, consistent with partial site saturation. The Δ*E* values (−1.63 and – 0.97 eV) align with the reported range (−0.23 to – 0.39 eV), though more stabilizing here due to the inclusion of a hybrid meta-GGA functional; this highlights the tendency of PBE-type periodic methods to underestimate interaction strength in electron-rich hydride complexes.

For Ni-decorated graphene, the first H_2_ adsorption is both electronically and thermodynamically favorable (Δ*E* = −1.14 eV, Δ*G* = −0.56 eV), indicating a stable interaction under standard conditions as shown in Table 2 and Figure 4(c). The H–H bond is significantly elongated (0.89 Å vs 0.74 Å in free H_2_), with short Ni–H distances (1.56–1.60 Å), consistent with a partially activated dihydrogen ligand. NBO analysis shows charge transfer from the metal to H_2_ (Δ*q* = −0.04 au, *q*(Ni) = +0.31 au), supporting a σ-donation/π-backdonation mechanism typical of a Kubas-type interaction.

In contrast, the second H_2_ adsorption remains electronically stabilizing (Δ*E* = −0.26 eV) but becomes thermodynamically unfavorable (Δ*G* = −0.26 eV), indicating dominance of entropy penalties. The weaker interaction is reflected in shorter H–H distances (0.83 Å) and longer Ni–H bonds (1.70–1.72 Å in Figure 4(c)). Although charge transfer increases (Δ*q* = −0.09 au), the reduced bond activation suggests less effective orbital overlap, highlighting that NBO charge alone does not track interaction strength.

The first adsorption step agrees well with previous DFT reports (∼ – 1.2 eV), supporting the reliability of the computational approach (Table 2). However, the second adsorption exhibits significantly stronger electronic stabilization than typically reported, where additional H_2_ binding is generally weak.

For Pd–G–H_2_, the Pd–H distances are the shortest in the series (1.71 Å) as indicated in Figure 5, whereas the H–H bond remains elongated (0.86 Å), emphasizing the balance between metal–hydrogen bond formation and H–H bond weakening. The nearly neutral NBO charge transfer to H_2_ (Δ*q* = 0.04 au) suggests a balance between donation and back-donation. The thermodynamic analysis reveals moderate exergonic stabilization (Δ*G* = −0.62 eV in Table 2), while the singlet spin state (*S* = 0*) indicates open-shell singlet electronic stability within the range favorable for reversible hydrogen storage; however, no stable adsorption of a second H_2_ molecule was observed. These features align with experimental evidence of Pd-decorated graphene acting as an efficient hydrogen dissociation platform.^17^ The computed Δ*E* (−0.94 eV) matches literature values (−0.73 to −0.96 eV) obtained with periodic DFT and nanoflake models, confirming that Pd maintains molecular activation rather than full dissociation.

**Figure 5:**
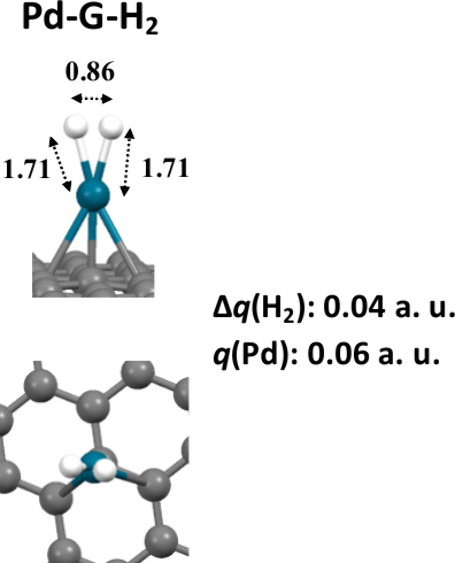
Optimized geometries of H_2_ complex with graphene decorated with Pd. The bond distance of M-H and H–H (in Å) are indicated as well as the NBO charge transfer.

High-level DLPNO–CCSD­(T) single-point calculations were carried out to benchmark the Pd–G–H_2_ (*S* = 0*) interaction energy (Table 3). The ωB97M-V//revPBE-D4 and DLPNO–CCSD­(T) results are in excellent quantitative agreement, both yielding an interaction energy of – 0.94 eV. The *T*_1_ diagnostic, which remains below 0.05 for this system (Table S5 in the Supporting Information), was used only as an auxiliary indicator that the correlated wave function does not exhibit severe single-reference breakdown; it was not taken as a standalone justification for adopting an RHF-based treatment. To evaluate the sensitivity of the correlated energies to the Hartree–Fock reference more directly, PNO–CCSD­(T) calculations were performed using both RHF and broken-symmetry UHF references for Pd–G and Pd–G–H_2_ (Table S6 in the Supporting Information). Although the RHF and UHF mean-field energies differ substantially, the final PNO–CCSD­(T) total energies are nearly identical: the RHF- and UHF-referenced values differ by only 0.000986 *E*_*h*_ for Pd–G and 0.000501 *E*_*h*_ for Pd–G–H_2_, corresponding to 0.62 and 0.31 kcal mol^–1^, respectively. Taken together, the agreement between ωB97M-V//revPBE-D4 and DLPNO–CCSD­(T), the absence of anomalously large *T*_1_ values, and the weak dependence of the final PNO–CCSD­(T) energies on the RHF versus broken-symmetry UHF reference support the use of the RHF-based correlated treatment for the Pd-containing systems examined here. This conclusion, however, should not be generalized to systems with stronger multiconfigurational character, for which an explicit multireference treatment may be required.

The spin density distributions for the Pd–G–H_2_ and Pd–G2–H_2_ systems (Figure 6) reveal a clear dependence of the electronic structure on the size of the graphene nanoflake. For the larger nanoflake (Pd–G–H_2_), the spin density exhibits partial delocalization over the graphene framework, particularly across the π-conjugated network surrounding the adsorption site. This behavior indicates electronic coupling between the Pd *d*-orbitals and the extended π-system of graphene, consistent with previous studies showing that larger graphene nanoflakes facilitate spin delocalization and metal–support electronic interaction.^54^

**Figure 6:**
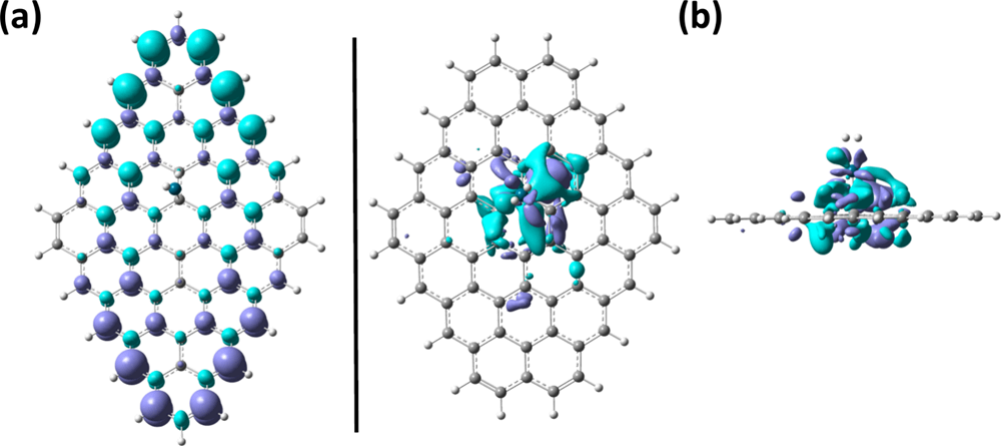
Spin density distributions for (a) Pd–G–H_2_ and (b) Pd–G2–H_2_ (top and side views) systems, calculated at the ωB97M-V //revPBE-D4 level of theory. Blue and green isosurfaces represent positive and negative spin density, respectively.

In contrast, for the smaller nanoflake (Pd–G2–H_2_), the spin density is more strongly localized around the Pd–H_2_ region, with minimal delocalization over the graphene lattice. This suggests a reduced interaction between the metal center and the π-system, leading to a more confined open-shell character. Such localization is consistent with the reduced polyradical character and electronic delocalization reported for smaller graphene nanostructures.^55^

On the other hand, this work focuses on the interaction energetics and adsorption mechanisms of H_2_ on TM-decorated graphene and therefore does not provide a quantitative gravimetric storage capacity (wt%), which would require explicit coverage/loading models and adsorption isotherm calculations.

### 3.4 Ab Initio Molecular Dynamics Simulations

Ab initio molecular dynamics (AIMD) simulations were performed for the Sc–G–2H_2_ and Pd–G–H_2_ systems as representative cases to probe their short-time dynamical behavior under finite-temperature conditions as shown in Figure 7. These systems were selected because their computed binding free energies (−0.29 and – 0.62 eV, respectively) span a physically relevant range for H_2_ adsorption on metal-decorated carbon supports, 0.2–0.6 eV,^67^ allowing comparison between moderately bound and more strongly interacting regimes without implying a priori dynamical stability or reversibility.

**Figure 7:**
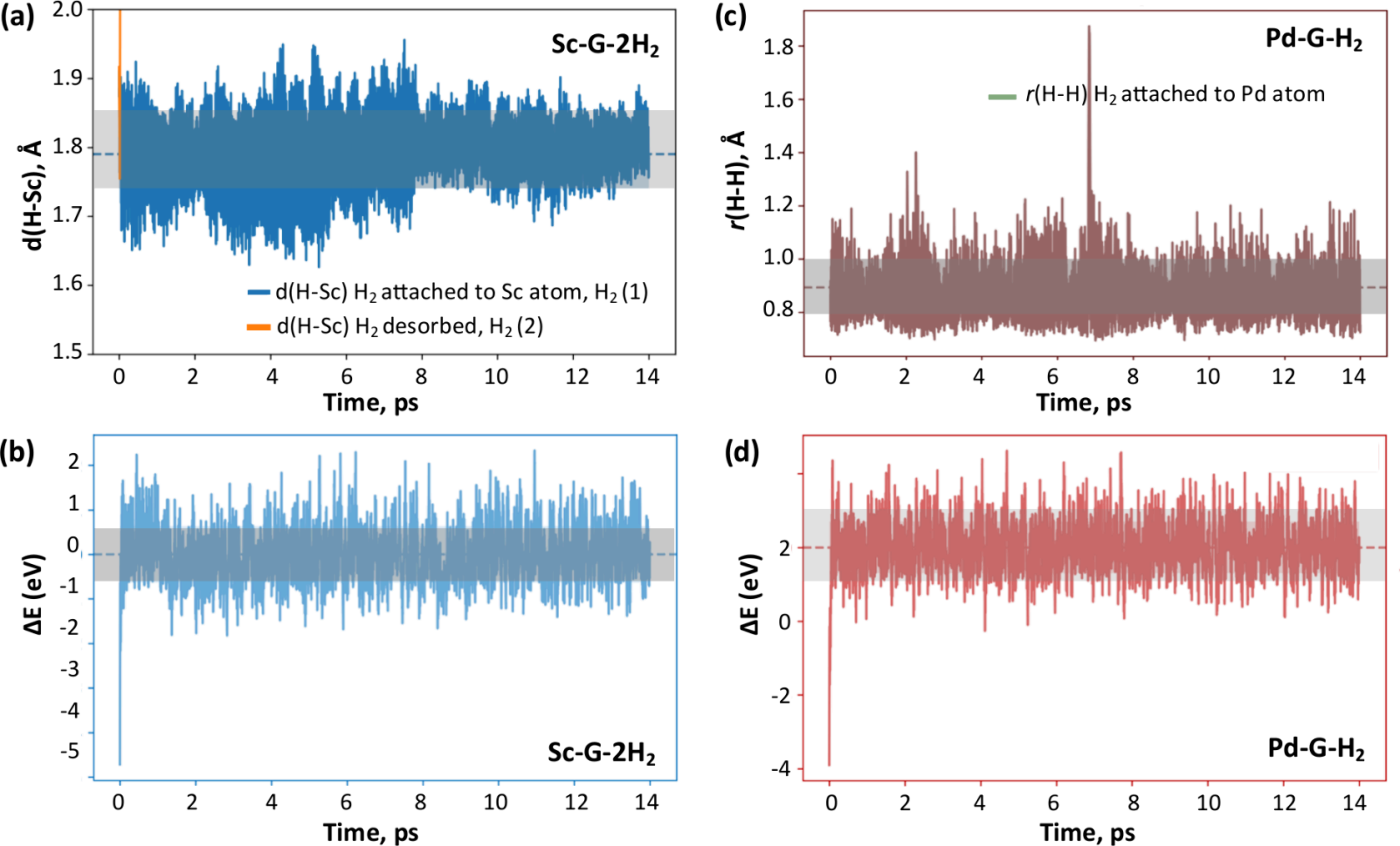
AIMD stability metrics for Sc- and Pd-decorated graphene systems at 500 K. (a) Time evolution of the average H–Sc distances for the two H_2_ molecules adsorbed on Sc-decorated graphene (Sc–G–2H_2_), with mean values of ⟨*d*_H–Sc_⟩ = 1.79 ± 0.05 Å (H_2_(1)) and 445.19 ± 249.35 Å (H_2_(2)). (b) Relative potential energy fluctuations for the Sc–G–2H_2_ system, reported as Δ*E*_pot_ (*t*) = *E*_pot_ (*t*) – ⟨*E*_pot_⟩, showing an average fluctuation of ⟨Δ*E*_pot_⟩ = 0 eV and a standard deviation of σ = 0.63 eV. (c) Time evolution of the H–H bond distance for H_2_ adsorbed on Pd-decorated graphene (Pd–G–H_2_), with ⟨*r*_H–H_⟩ = 0.89 ± 0.11 Å. (d) Relative potential energy fluctuations for the Pd–G–H_2_ system, expressed as Δ*E*_pot_ (*t*) = *E*_pot_ (*t*) – ⟨*E*_pot_⟩, with ⟨Δ*E*_pot_⟩ = 0 eV and σ = 0.70 eV. In Figures 7(b) and 7(d), the dashed horizontal line marks the mean value, and the shaded region corresponds to ± 1 standard deviation (±1σ). Energies are reported in eV.

For the Sc–G system with two H_2_ molecules, the analysis of H–H distances (Figure S4 in Supporting Information) revealed that one of the H_2_ molecules departs from the adsorption site, as reflected by the continuous increase in the H–Sc distance beyond 440 Å (Figure 7(a)). This behavior corresponds to desorption rather than stable multimolecule adsorption under the simulated conditions. In contrast, the second H_2_ molecule remains in proximity to the Sc center, maintaining a bond distance consistent with molecular adsorption over the sampled time scale.

In addition, the AIMD trajectory shows that the Sc center undergoes positional fluctuations across the graphene nanoflake, indicating a degree of surface mobility under finite-temperature conditions. Such behavior is consistent with previous studies of transition-metal adatoms on graphene,^68^ where relatively low diffusion barriers can enable thermally activated migration between adsorption sites in the absence of strong anchoring defects. This observation suggests that the metal–support interaction may not be sufficiently rigid to fully suppress surface diffusion on short time scales, although the limited duration of the simulation does not allow for a quantitative assessment of diffusion barriers or long-term structural stability.

The relative potential energy profile (Figure 7(b)) fluctuates around a well-defined mean value after an initial equilibration period, as expected for a thermostated trajectory, indicating that the system remains numerically stable over the sampled time scale. These fluctuations are consistent with thermal motion around accessible configurations and provide a qualitative picture of the system’s short-time dynamical behavior. However, such observations alone are not sufficient to establish thermodynamic stability or resistance to desorption. A qualitatively similar behavior is observed in the simulations at 300 K (Figure S5), although the limited sampling prevents quantitative comparison or assessment of long-time scale stability.

For the Pd–graphene system with one H_2_ molecule, the H–H bond distance (Figure 7(c)) exhibits fluctuations between 0.8 and 1.0 Å with occasional elongations, suggesting partial activation of H_2_ under Pd coordination without full dissociation over the sampled trajectory. The H–Pd distances (Figure S6 in the Supporting Information) stabilized around 1.7–1.9 Å, consistent with partial activation of H_2_ without complete dissociation. The AIMD simulation also suggests that the Pd atom exhibits surface mobility across the graphene nanoflake, as evidenced by its positional fluctuations during the trajectory. This behavior is consistent with relatively low diffusion barriers enabling thermally activated migration. The corresponding potential energy fluctuations (Figure 7(d)) remain bounded after equilibration, indicating a stable numerical trajectory over the sampled time scale, but without implying long-term thermodynamic or kinetic stability.

Overall, these results reveal distinct short-time dynamical responses for Sc and Pd centers: in the Sc system, one H_2_ molecule undergoes desorption and the metal center exhibits noticeable surface mobility, whereas in the Pd system the interaction with H_2_ is maintained, accompanied by bond elongation without full dissociation over the sampled trajectory. It is important to emphasize that these AIMD simulations are limited to short time scales (up to 14 ps) and are performed within a canonical (NVT) ensemble without enhanced sampling techniques; therefore, they do not provide direct information on kinetic barriers, rare events, or long-time scale stability.

## 4 Conclusions

In this work, hydrogen adsorption on transition-metal–decorated graphene was examined within a finite nanoflake framework using revPBE-D4 geometries, ωB97M-V single-point energies, and selected post-Hartree–Fock benchmarks. The results suggest that hydrogen adsorption on transition-metal–decorated graphene cannot be understood without explicitly considering the electronic structure of the graphene support. The larger graphene nanoflake G exhibits a robust open-shell/multiconfigurational singlet character, whereas the smaller G2 model shows a weaker and more functional-dependent tendency toward an open-shell description. This size-dependent electronic structure may modulate metal binding and adsorption energetics by altering the local electronic environment of the active site.

Multireference calculations further refine this picture. CASSCF­(12,12)/NEVPT2 identifies a spin-adapted singlet ground state for G, 0.19 eV below the triplet, with natural orbital occupations consistent with biradicaloid and moderately multiconfigurational character. In addition, CASSCF­(11,12)/NEVPT2 calculations corroborate the DFT high-spin ground-state assignments for Sc–G and Y–G.

At the metal–graphene level, Y, Nb, Pd, Ni, Zr, and Sc form favorable complexes of varying strength (−0.35 to −1.02 eV), whereas Cr, Fe, Co, Cu, Mo, and W are weakly bound or unfavorable for the nanoflake considered here. Upon hydrogen adsorption, the first H_2_ molecule binds exergonically to Y, Zr, Nb, Ni, and Pd (−0.56 to −1.63), while Sc becomes thermodynamically favorable only after adsorption of a second H_2_ molecule with a Gibbs free energy of −0.29 eV. Among the systems studied, Y, Zr, and Nb show pronounced H_2_ activation and hydride-like character, whereas Pd and Ni retain more molecularly activated H_2_. Additional H_2_ adsorption is generally disfavored beyond the first step for Y, Nb, and Ni, while Zr remains favorable for the second adsorption step but becomes slightly endergonic for the third and fourth additions.

We selected three representative systems (Pd, Zr, and Sc) that span distinct bonding regimes (molecular adsorption, dissociative hydride formation, and high-spin configurations). These cases were selected to assess the performance of the methodology across chemically distinct bonding regimes. For Pd–G–H_2_, Zr–G–H_2_, and Sc–G–H_2_, ωB97M-V//revPBE-D4 reproduces coupled-cluster interaction energies very closely. It provides evidence that the chosen methodology performs consistently across different bonding regimes relevant to this study. For the Pd-containing systems, the final PNO–CCSD­(T) energies obtained from RHF and broken-symmetry UHF references differ by only 0.62 kcal mol^–1^ for Pd–G and 0.31 kcal mol^–1^ for Pd–G–H_2_, indicating that the correlated benchmark energies are not materially affected by the reference choice in these cases.

Spin-density analysis of the Sc and Pd systems suggests that while the magnetic character remains centered at the metal site, the extent of spin delocalization and metal–support interaction is strongly modulated by the size of the graphene nanoflake, which in turn influences the electronic structure of the active site.

AIMD simulations further reveal qualitatively distinct dynamical responses for Sc and Pd: in Sc–G–2H_2_, one H_2_ molecule desorbs and the metal center displays surface mobility, whereas in Pd–G–H_2_ the H_2_ molecule remains coordinated with bond elongation but without full dissociation over the sampled trajectory.

Overall, these findings establish that spin-state effects and finite-size electronic structure are critical factors influencing hydrogen adsorption on graphene-based materials. They also demonstrate that ωB97M-V//revPBE-D4, when combined with a finite-cluster framework, yields interaction energies comparable in magnitude to relevant experimental reference values, periodic DFT trends, and high-level PNO–CCSD­(T) and DLPNO–CCSD­(T) results, supporting its reliability as a practical and accurate approach for studying transition-metal–decorated graphene in hydrogen storage applications.

## Supplementary Material



## Data Availability

All data supporting this study are included within the article and the Supporting Information and are available from the corresponding author upon reasonable request.

## Notes

Comparison with experimental data; CASSCF/NEVPT2 results; complete energetic data and structural details of all studied species; AIMD snapshots; and XYZ coordinates of all systems (PDF)
