# Supplementary material for: Hydrogen Adsorption on Transition-Metal-Decorated Graphene: Thermodynamic and AIMD Insights from DFT and Post-Hartree–Fock Methods
Source: J Phys Chem A. 2026 Jun 26;130(27):5115–26. doi: 10.1021/acs.jpca.5c07741 (PMC13359363; doi:10.1021/acs.jpca.5c07741)
Supplement: Supplementary file 1 [file jp5c07741_si_001.pdf]

Supporting Information

Hydrogen Adsorption on Transition-Metal–Decorated Graphene: Thermodynamic and AIMD Insights from DFT and Post-Hartree–Fock Methods

Wilmer Esteban Vallejo Narváez,[a*] Cesar Gabriel Vera de la Garza,[a] Serguei Fomine[a*]

[a] Instituto de Investigaciones en Materiales, Universidad Nacional Autónoma de México, Apartado Postal 70-360, CU, Coyoacán, 04510 Ciudad de México, México. E-mail: wilmervall@comunidad.unam.mx, fomine@unam.mx

Contents

		Page
1.	Figure S1. Comparison of structural parameters calculated by DFT with experimental data.	3
2.	Table S1. Comparison of binding dissociation energies calculated by DFT with experimental data.	4
3.	Table S2. Evaluation of electronic structure of G and G2.	5
4.	Table S3. Total energies, Gibbs free energies, and spin states of all species studied.	6
5.	Table S4. Evaluation of the lowest-energy spin state for Sc and Y systems.	9
6.	Table S5. T1 diagnostic for selected open-shell and high-spin systems.	10
7.	Table S6. Dependence of the PNO-CCSD(T) total electronic energies on the Hartree–Fock reference for the Pd–G and Pd–G–H_2 systems.	11
8.	Figure S2. Spin density plots for Sc-G and Sc-G-H_2 complexes.	12
9.	Figure S3. Optimized geometries of 3H_2- and 4H_2-complexes with Zr- decorated graphene.	13
10.	Figure S4. Average Sc–C distances and H–H distances for two H_2 molecules adsorbed on Sc-decorated graphene during AIMD simulations at 500 K.	14
11.	Figure S5. H–H distances for two adsorbed H_2 molecules, average Sc–C distances, relative potential energy, and Sc–H distances for the Sc–G–2H_2 complex during AIMD simulations at 300 K.	15
12.	Figure S6. Pd–H distances for one H_2 molecule adsorbed and average Pd–C distances for the complex Pd-G-H_2 during AIMD simulations at 500 K.	16
13.	XYZ coordinates of the species.	17

(a)

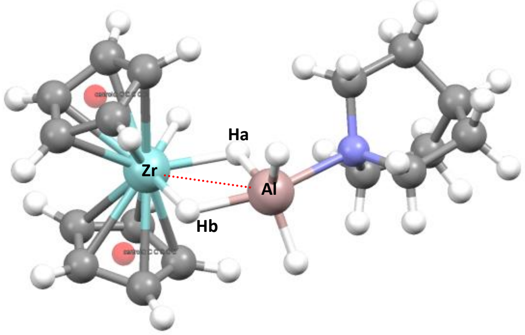

Selected distances (Å)			
**Pair of atoms**	**Exp.[1]**	**revPBE-D4**	**ωb97M-D4**
**Zr-Ha**	1.91	1.94	1.96
**Zr-Hb**	1.83	1.92	1.93
**Zr-Al**	3.00	2.98	2.98
**Zr-Centroid 1**	2.193	2.209	2.213
**Zr-Centroid 2**	2.199	2.209	2.212

(b)

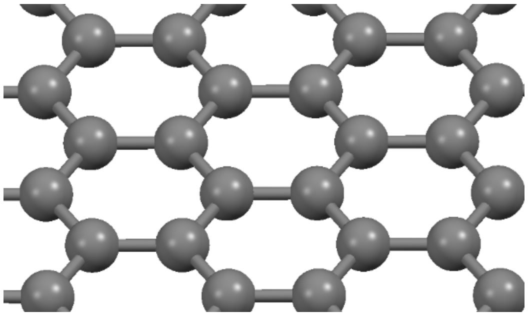

Distances (Å)			
**Pair of atoms**	**Exp. [2]**	**revPBE-D4**	**ωb97M-D4**
**C-C**	1.421	1.419 – 1.426	1.401 -1.418

Figure S1. (a) Selected bond distances (in Å) for a hydride-bridged heterobimetallic zirconium–aluminum complex obtained from X-ray crystallography, and (b) the experimental C–C bond length in graphene, together with values calculated at the revPBE-D4 and ωB97M-D4 levels of theory using the def2-mTZVPP basis set.

[1] Khan, K.; Raston, C. L.; McGrady, J. E.; Skelton, B. W.; White, A. H. Hydride-Bridged heterobimetallic complexes of zirconium and aluminum. *Organometallics* **1997**, *16* (15), 3252–3254. https://doi.org/10.1021/om970141i

[2] Baskin, Y.; Meyer, L. Lattice constants of graphite at low temperatures. *Physical Review* **1955**, *100* (2), 544. https://doi.org/10.1103/physrev.100.544

Table S1. Experimental binding dissociation energies (BDEs) for Sc and Ni cation complexes corresponding to H_2 loss, along with calculated values obtained at the revPBE-D4 and ωB97M-V//revPBE-D4 levels of theory using the def2-mTZVPP basis set. Experimental BDE references are indicated by superscript numbers and detailed in the corresponding footnotes. $-\Delta H_0^o$ corresponds to the bond dissociation energy at 0 K, including zero-point energy (ZPE) correction.

Reaction	Experimental BDE	revPBE-D4	ωB97M-V//revPBE-D4
	$-\Delta H_0^o$ in kcal/mol		
$Sc^+(H_2)_2 \rightarrow Sc^+(H_2) + H_2$	6.40 ± 0.50 [3]	6.05	6.21
$Ni^+(H_2)_4 \rightarrow Ni^+(H_2)_3 + H_2$	7.10 ± 0.30 [4]	8.04	7.05

[3] Bushnell, J. E.; Kemper, P. R.; Maitre, P.; Bowers, M. T. Insertion of Sc+ into H2: The First Example of Cluster-Mediated .sigma.-Bond Activation by a Transition Metal Center. *Journal of the American Chemical Society* **1994**, *116* (21), 9710–9718. https://doi.org/10.1021/ja00100a041

[4] Kemper, P. R.; Weis, P.; Bowers, M. T. Ni+(H2) : Ligand bond energies for ground state ions. *Chemical Physics Letters* **1998**, *293* (5–6), 503–510. https://doi.org/10.1016/s0009-2614(98)00831-8

Table S2. Electronic energies (E_h), ⟨S^2⟩ values, and relative energies for different spin states of graphene nanoflakes of two sizes, G (92 atoms) and G2 (84 atoms), computed at the revPBE-D4/def2-mTZVPP, ωB97M-V/def2-mTZVPP//revPBE-D4/def2-mTZVPP, ωB97M-D4/def2-TZVP(-f), and CASSCF(12,12)/NEVPT2/def2-mTZVPP levels of theory. The CASSCF(12,12)/NEVPT2 calculations were performed on geometries optimized at the revPBE-D4/def2-mTZVPP level. Relative energies (ΔE) are given in eV with respect to the lowest-energy state of each method.

**G**	**revPBE-D4**			**ωb97M-V//revPBE-D4**			**ωb97M-D4**			**CASSCF (12,12)/ NEVPT2[a]**	
**spin state**	E, E_h	⟨S^2⟩	ΔE (eV)	E, E_h	⟨S^2⟩	ΔE (eV)	E, E_h	⟨S^2⟩	ΔE (eV)	**E, E_h**	ΔE (eV)
**OSS**	**-2681.7872**	**0.890**	**0.00**	**-2681.0355**	**2.378**	**0.00**	**-2682.9088**	**2.279**	**0.00**		
singlet	-2681.7846	0.000	0.07	-2680.9734	0.000	1.69	-2682.8580	0.000	1.38	**-2674.0294[b]**	**0.00**
triplet	-2681.7838	2.032	0.09	-2681.0155	2.222	0.54	-2682.8942	2.198	0.40	-2674.0224	0.19

[a] NEVPT2 energies computed using a CASSCF(12,12) reference. Natural orbital occupations used for electronic-structure assignment are those of the CASSCF(12,12) reference.

[b] These occupations, 1.97288, 1.97102, 1.96677, 1.92510, 1.78971, 1.33660, 0.66504, 0.21215, 0.07357, 0.03173, 0.02859, and 0.02686, deviate substantially from the ideal closed-shell pattern of 2, 2, 2, 2, 2, 2, 0, 0, 0, 0, 0, 0. In particular, the strongly fractional occupations of 1.33660 and 0.66504 indicate two partially occupied active orbitals, consistent with biradicaloid character. The additional fractional occupations, especially 1.78971 and 0.21215, suggest that this state is not a pure two-orbital biradical but rather a moderately multiconfigurational singlet.

**G2**	**revPBE-D4**			**ωb97M-V//revPBE-D4**			**ωb97M-D4**		
**spin state**	E, E_h	⟨S^2⟩	ΔE (eV)	E, E_h	⟨S^2⟩	ΔE (eV)	E, E_h	⟨S^2⟩	ΔE (eV)
**OSS**	**-2451.8978**	**0.000**	**0.00**	**-2451.2087**	**0.865**	**0.00**	**-2452.9229**	**0.286**	**0.00**
singlet	-2451.8978	0.000	0.00	-2451.2051	0.000	0.10	-2452.9228	0.000	0.00
triplet	-2451.8735	2.025	0.66	-2451.1837	2.246	0.68	-2452.8978	2.221	0.68

Table S3. Electronic, Gibbs free energies in E_h, total spin (S), $\langle S^2 \rangle$ and ideal $\langle S^2 \rangle$ values of all species studied with ωb97M-D4/def2-TZVP(-f), revPBE-D4/def2-mTZVPP and ωb97M-V/def2-mTZVPP//revPBE-D4/def2-mTZVPP level of theories. The energy values reported in Tables 1 and 2, as well as in Figure 2 of the main text, were obtained for the most stable electronic state (i.e., the lowest energy configuration). For each system, we first compared the energies of the species with different spin states (Table S3), and the lowest-energy spin state was selected for reporting. S = 0* label denotes an unrestricted broken-symmetry (open-shell singlet) solution used to approximate an antiferromagnetically coupled singlet-like state.

				ωb97M-D4				revPBE-D4			ωb97M-V //revPBE-D4	
Species	*mul*	*S*	*Spin state*	*Electronic* (E_h)	*Gibbs* (E_h)	$\langle S^2 \rangle$	Ideal $\langle S^2 \rangle$	*Electronic* (E_h)	*Gibbs* (E_h)	$\langle S^2 \rangle$	*Electronic* (E_h)	$\langle S^2 \rangle$
Graphene (G)	1	0	singlet	-2682.8580	-2682.2307	0.000	0.00					0.000
	3	1	triplet	-2682.8942	-2682.2690	2.198	2.00					2.222
	1	0*	OSS	-2682.9088	-2682.2835	2.279	0.00	-2681.7871	-2681.1896	0.890	-2681.0355	2.378
Sc atom	2	1/2	doublet	-760.6595	-760.6756	0.753	0.75	-760.5032	-760.5192	0.753	-760.5702	0.753
	4	3/2	quartet	-760.6221	-760.6388	3.751	3.75					
	6	5/2	sextet	-759.6122	-759.6292	8.751	8.75					
Sc-G	2	1/2	doublet	-3443.5743	-3442.9613	3.054	0.75					
	4	3/2	quartet	-3443.5772	-3442.9643	5.192	3.75					
	6	5/2	sextet	-3443.5822	-3442.9691	9.186	8.75	-3442.3415	-3441.7521	8.807	-3441.6259	9.233
Sc-G-H_2	2	1/2	doublet	-3444.7514	-3444.1264	3.159	0.75					
	4	3/2	quartet	-3444.7502	-3444.1265	5.285	3.75					
	6	5/2	sextet	-3444.7620	-3444.1356	9.192	8.75	-3443.5281	-3442.9260	8.813	-3442.7960	9.256
Sc-G-2H_2	2	1/2	doublet	-3445.9319	-3445.2954	3.028	0.75					
	4	3/2	quartet	-3445.9568	-3445.3165	4.184	3.75	-3444.7230	-3444.1088	3.810	-3443.9783	4.226
	6	5/2	sextet	-3445.9499	-3445.3100	9.205	8.75					
Y atom	2	1/2	doublet	-38.1890	-38.2060	0.760	0.75	-38.2516	-38.2686	0.991	-38.1462	0.759
	4	3/2	quartet	-38.1402	-38.1578	3.751	3.75					
	6	5/2	sextet	-37.2745	-37.2925	8.751	8.75					
Y-G	2	1/2	doublet	-2721.1098	-2720.4973	3.045	0.75					
	4	3/2	quartet	-2721.1275	-2720.5175	4.289	3.75	-2720.0847	-2719.4954	4.652	-2719.2192	4.595
	6	5/2	sextet	-2721.1194	-2720.5065	9.230	8.75					
Y-G-H_2	2	1/2	doublet	-2722.3473	-2721.7256	2.096	0.75					
	4	3/2	quartet	-2722.3532	-2721.7323	4.191	3.75	-2721.3117	-2720.7120	3.805	-2720.4291	4.215
	6	5/2	sextet	-2722.2906	-2721.6674	9.162	8.75					
Y-G-2H_2	2	1/2	doublet	-2723.5261	-2722.8915	2.075	0.75	-2722.5002	-2721.8824	0.770	-2721.5897	2.113
	4	3/2	quartet	-2723.4876	-2722.8502	4.169	3.75					
	6	5/2	sextet	-2723.4764	-2722.8388	9.235	8.75					
Zr atom	1	0	singlet	-46.8252	-46.8416	0.000	0.00					
	3	1	triplet	-46.8635	-46.8809	2.002	2.00	-46.9350	-46.9524	2.518	-46.8166	2.002
	5	2	quintuplet	-46.8463	-46.8642	6.002	6.00					
Zr-G	1	0	singlet	-2729.7319	-2729.1148	0.000	0.00					
	3	1	triplet	-2729.7895	-2729.1776	3.806	2.00					
	5	2	quintuplet	-2729.7876	-2729.1751	6.569	6.00					
	1	0*	OSS	-2729.7927	-2729.1797	3.478	0.00	-2728.7816	-2728.1893	1.436	-2727.8648	3.364
Zr-G-H_2	1	0	singlet	-2729.1093	-2729.7319	0.000	0.00					
	3	1	triplet	-2730.4153	-2729.7895	3.525	2.00					
	5	2	quintuplet	-2730.4121	-2729.7876	6.457	6.00					
	1	0*	OSS	-2731.0276	-2730.4050	2.422	0.00	-2730.0086	-2729.4042	0.967	-2729.0964	2.899
Zr-G-2H_2	1	0	singlet	-2731.8695	-2731.4570	0.000	0.00					
	3	1	triplet	-2732.2010	-2731.5612	3.258	2.00					
	1	0*	OSS	-2732.2162	-2731.5748	2.407	0.00	-2731.1970	-2730.5793	0.236	-2730.2810	2.435

Zr-G-3H_2	1	0	singlet	-2733.3630	-2732.7025	0.000	0.00					
	3	1	triplet	-2733.3882	-2732.7340	3.503	2.00					
	5	2	quintuplet	-2733.3900	-2732.7368	6.437	6.00	-2732.3782	-2731.7491	6.057	-2731.4510	6.463
	1	0*	OSS	-2733.3841	-2732.7291	2.116	0.00					
Zr-G-4H_2	1	0	singlet	-2734.5579	-2733.8570	0.000	0.00					
	3	1	triplet	-2734.5760	-2733.9090	3.495	2.00					
	5	2	quintuplet	-2734.5783	-2733.9113	6.436	6.00	-2733.5653	-2732.9213	6.058	-2732.6248	6.467
	1	0*	OSS	-2734.5702	-2733.9019	2.281	0.00					
Pd atom	1	0	singlet	-127.8684	-127.8851	0.000	0.00	-127.9286	-127.9452	0.000	-127.7968	0.000
	3	1	triplet	-127.8296	-127.8473	2.001	2.00					
	1	0	singlet	-127.8684	-127.8851	0.000	0.00					
Pd-G	1	0	singlet	-2810.7556	-2810.1332	0.000	0.00					
	3	1	triplet	-2810.7923	-2810.1730	2.206	2.00					
	5	2	quintuplet	-2810.7864	-2810.1689	6.323	6.00					
	1	0*	OSS	-2810.8065	-2810.1875	2.273	0.00	-2809.7758	-2809.1785	0.886	-2808.8617	2.378
Pd-G-H_2 Cx_I	1	0	singlet	-2811.9534	-2811.3189	0.000	0.00					
	3	1	triplet	-2811.9901	-2811.3587	2.204	2.00					
	1	0*	OSS	-2812.0043	-2811.3747	2.278	0.00	-2810.9790	-2810.3717	0.888	-2810.0542	2.376
Cx_II	1	0*	OSS	-2811.9994	-2811.3672	2.291	0.00					
Nb atom	2	1/2	doublet	-56.7501	-56.7671	2.013	0.75					
	4	3/2	quartet	-56.7753	-56.7930	3.755	3.75	-56.8558	-56.8735	4.474	-56.7244	3.754
	6	5/2	sextet	-56.7347	-56.7528	8.751	8.75					
Nb-G	4	3/2	quartet	-2739.7009	-2739.0887	5.138	3.75					
	6	5/2	sextet	-2739.7100	-2739.0961	10.650	8.75	-2738.7146	-2738.1205	9.434	-2737.7847	10.351
Nb-G-H_2	4	3/2	quartet	-2740.9263	-2740.3017	5.057	3.75					
	6	5/2	sextet	-2740.9344	-2740.3117	9.250	8.75	-2739.9269	-2739.3231	8.821	-2739.0026	9.250
Nb-G-2H_2	2	1/2	doublet	-2741.4817	-2742.1253	3.169	0.75	-2741.1277	-2740.5057	1.126	-2740.1720	3.045
	4	3/2	quartet	-2741.4609	-2742.1014	6.243	3.75					
	6	5/2	sextet	-2741.4803	-2742.1227	9.225	8.75					
Ni atom	1	0	singlet	-1508.2502	-1508.2660	0.000	0.00					
	3	1	triplet	-1508.3091	-1508.3259	2.003	2.00	-1508.1643	-1508.1811	2.001	-1508.1767	2.002
	5	2	quintuplet	-1508.1329	-1508.1502	6.001	6.00					
	7	3	septuplet	-1507.8020	-1507.8196	12.001	12.00					
Ni-G	1	0	singlet	-4191.1723	-4190.5525	0.000	0.00					
	3	1	triplet	-4191.2049	-4190.5860	2.509	2.00					
	5	2	quintuplet	-4191.2154	-4190.6020	6.385	6.00					
	7	3	septuplet	-4191.2087	-4190.5920	12.326	12.00					
	1	0*	OSS	-4191.2221	-4190.6045	2.351	0.00	-4190.0139	-4189.4208		-4189.2292	2.406
Ni-G-H_2	3	1	triplet	-4192.4179	-4191.7868	3.317	2.00					
	5	2	quintuplet	-4192.4147	-4191.7881	6.390	6.00					
	1	0*	OSS	-4192.4366	-4191.8067	2.326	0.00	-4191.2360	-4190.6233	0.845	-4190.4291	2.363
Ni-G-2H_2	3	1	triplet	-4193.6040	-4192.9570	3.346	2.00					
	1	0*	OSS	-4193.6202	-4192.9724	2.294	0.00	-4192.4205	-4191.7864	0.905	-4191.6003	2.394
Fe atom	1	0	singlet	-1263.5768	-1263.5925	0.000	0.00					
	3	1	triplet	-1263.6012	-1263.6881	2.001	2.00					
	5	2	quintuplet	-1263.6994	-1263.7166	6.006	6.00	-1263.5495	-1263.5667	6.005	-1263.5847	6.005
	7	3	septuplet	-1263.5642	-1263.5817	12.002	12.00					
Fe-G	1	0	singlet	-3946.4762	-3945.8563	0.000	0.00					
	3	1	triplet	-3946.5515	-3945.9374	4.254	2.00					
	5	2	quintuplet	-3946.5912	-3945.9776	7.339	6.00	-3945.3846	-3944.7886	6.810	-3944.6151	8.378
	7	3	septuplet	-3946.5714	-3945.9576	13.323	12.00					
	1	0*	OSS	-3946.5507	-3945.9358	3.281	0.00					
W atom	1	0	singlet	-66.8018	-66.8192	0.000	0.00					
	3	1	triplet	-66.8468	-66.8652	3.246	2.00					
	5	2	quintuplet	-66.8883	-66.9072	6.074	6.00					
	7	3	septuplet	-66.8975	-66.9168	12.002	12.00	-67.0013	-67.0205	12.001	-66.8275	12.002
W-G	1	0	singlet	-2749.7350	-2749.1158	0.000	0.00					
	1	0*	OSS	-2749.7608	-2749.1449	3.618	0.00					
	3	1	triplet	-2749.7741	-2749.1593	4.512	2.00					
	5	2	quintuplet	-2749.7786	-2749.1647	6.675	6.00					
	7	3	septuplet	-2749.7777	-2749.1658	12.437	12.00	-2748.7917	-2748.2025	12.147	-2747.8335	12.548

Cu atom	2	1/2	doublet	-1640.5179	-1640.5344	0.750	0.75	-1640.3816	-1640.3981	0.752	-1640.3853	0.750
	4	3/2	quartet	-1640.2803	-1640.2975	3.751	3.75					
	6	5/2	sextet	-1639.9095	-1639.9270	8.750	8.75					
	8	7/2	octet	-1639.3234	-1639.3412	15.751	15.75					
Cu-G	2	1/2	doublet	-4323.4078	-4322.7935	2.060	0.75					
	4	3/2	quartet	-4323.4140	-4322.7981	4.140	3.75	-4322.1980	-4321.6022	3.787	-4321.4155	4.518
	6	5/2	sextet	-4323.4159	-4322.7959	9.071	8.75					
	8	7/2	octet	-4323.3552	-4322.7395	16.176	15.75					
Cr atom	1	0	singlet	-1044.3057	-1044.3213	0.000	0.00					
	3	1	triplet	-1044.3722	-1044.3888	3.997	2.00					
	5	2	quintuplet	-1044.3805	-1044.3976	7.001	6.00					
	7	3	septuplet	-1044.4581	-1044.4756	12.000	12.00	-1044.3307	-1044.3481	12.000	-1044.3542	12.000
	9	4	nonet	-1042.8905	-1042.9082	20.001	20.00					
Cr-G	5	2	quintuplet	-3727.3605	-3726.7430	9.173	6.00					
	7	3	septuplet	-3727.3586	-3726.7449	13.316	12.00	-3726.1583	-3725.5613	12.550	-3725.3903	14.334
Mo atom	1	0	singlet	-67.9238	-67.9403	0.000	0.00					
	3	1	triplet	-67.9886	-68.0060	3.922	2.00					
	5	2	quintuplet	-68.0365	-68.0544	7.000	6.00					
	7	3	septuplet	-68.0678	-68.0861	12.001	12.00	-68.1641	-68.1824	12.000	-68.0132	12.001
Mo-G	1	0	singlet	-2750.9050	-2750.2845	0.000	0.00					
	1	0*	OSS	-2750.9562	-2750.3390	2.523	0.00	-2749.9482	-2749.3550	1.260	-2749.0335	2.631
	3	1	triplet	-2750.9426	-2750.3269	3.293	2.00					
	5	2	quintuplet	-2750.9474	-2750.3321	6.380	6.00					
Co atom	2	1/2	doublet	-1382.7479	-1382.7643	1.756	0.75					
	4	3/2	quartet	-1382.7594	-1382.7693	3.754	3.75	-1382.6123	-1382.6294	3.751	-1382.6398	3.753
	6	5/2	sextet	-1382.6066	-1382.6240	8.751	8.75					
	8	7/2	octet	-1382.3230	-1382.3407	15.751	15.75					
Co-G	2	1/2	doublet	-4065.6586	-4065.0466	2.037	0.75					
	4	3/2	quartet	-4065.6581	-4065.0461	4.124	3.75					
	6	5/2	sextet	-4065.6642	-4065.0519	9.150	8.75	-4064.4371	-4063.8414	8.827	-4063.6717	9.170
	8	7/2	octet	-4065.6527	-4065.0403	16.327	15.75					

Table S4. Electronic energies were computed at the CASSCF (11,12)/NEVPT2 level of theory using the def2-mTZVPP basis set for the doublet, quartet, and sextet states of the Sc–G and Y–G complexes, with geometries optimized at the revPBE-D4/def2-mTZVPP level. Relative energies (ΔE) are given in eV with respect to the lowest-energy state of each system at the CASSCF/NEVPT2 level.

System	Spin state	E(CASSCF/NEVPT2) (E_h)	ΔE (eV)
**Sc–G**	**5/2**	**-3434.015653**	**0.00**
Sc–G	1/2	-3434.001385	+0.39
Sc–G	3/2	-3433.985295	+0.83
**Y–G**	**3/2**	**-2712.115802**	**0.00**
Y–G	1/2	-2712.108012	+0.21
Y–G	5/2	-2712.089412	+0.72

The same active space, CASSCF (11,12), was employed for all spin states (doublet, quartet, and sextet) of the Sc–G and Y–G complexes. The selected orbitals correspond to metal-centered d orbitals and graphene π orbitals involved in the metal–surface interaction. Natural orbital occupation numbers show a consistent distribution across spin states, indicating that the chosen active space provides a balanced description of all multiplicities.

Table S5. T1 diagnostic values calculated at the DLPNO-CCSD(T)/def2-mTZVPP//revPBE-D4/def2-mTZVPP level of theory for selected *open-shell* singlet and high-spin ground state species studied in this work. In parentheses is indicated the most stable spin state.

Species	T1 diagnostic
Pd-H_2-G (OSS)	0.013
Pd-G (OSS)	0.013
Zr-H_2-G (OSS)	0.012
Zr-G (OSS)	0.013
Sc-H_2-G (5/2)	0.022
Sc-G (5/2)	0.021

Table S6. Dependence of the PNO-CCSD(T)/def2-mTZVPP//revPBE-D4/def2-mTZVPP total electronic energies on the Hartree–Fock reference for the Pd–G and Pd–G–H_2 systems. Energies are reported in Hartree. ΔE_{ref} corresponds to the absolute energy difference between calculations initiated from the RHF and broken-symmetry UHF references at each level of theory. Values in parentheses are given in kcal mol^{-1}, using 1 E_h = 627.509474 kcal mol^{-1}.

System	Level of theory	RHF-reference energy (E_h)	OSS/UHF-reference energy (E_h)	ΔE_{ref} (E_h)	ΔE_{ref} (kcal mol^{-1})
Pd–G	HF	-2791.58596	-2791.87278	0.28681	179.98
	CCSD	-2801.13794	-2801.01522	0.12272	77.01
	CCSD(T)	-2801.67369	-2801.67270	0.00099	0.62
Pd–G-H_2	HF	-2792.71168	-2792.91330	0.20162	126.52
	CCSD	-2802.33144	-2802.24402	0.08742	54.86
	CCSD(T)	-2802.86966	-2802.86916	0.00050	0.31

The substantial energy differences between the RHF and broken-symmetry UHF solutions at the mean-field level decrease markedly upon inclusion of electron correlation. At the PNO-CCSD(T) level, the reference dependence is reduced to 0.62 kcal mol^{-1} for Pd–G and 0.31 kcal mol^{-1} for Pd–G–H_2, indicating that the final correlated energies are essentially insensitive to the choice of RHF or OSS/UHF reference for these systems.

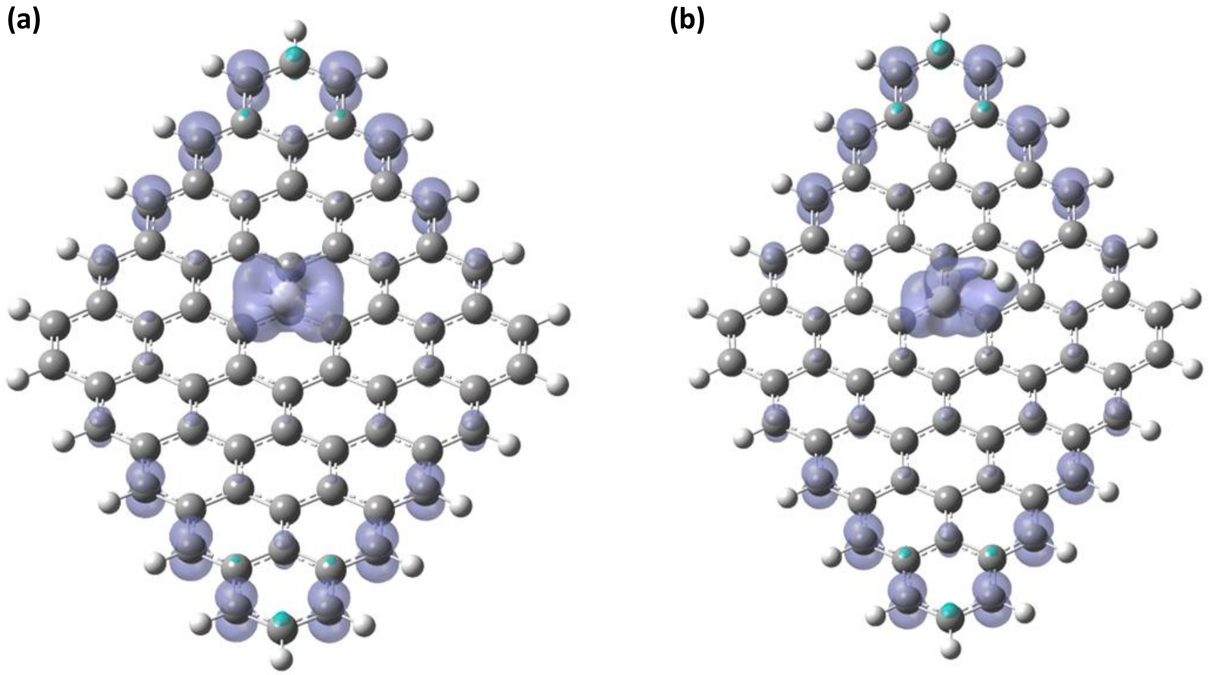

Figure S2. Spin density distribution for (a) Sc–G and (b) Sc–G–H_2 systems calculated at the ωB97M-V/def2-mTZVPP//revPBE-D4/def2-mTZVPP level of theory. Blue and green isosurfaces represent positive and negative spin density, respectively.

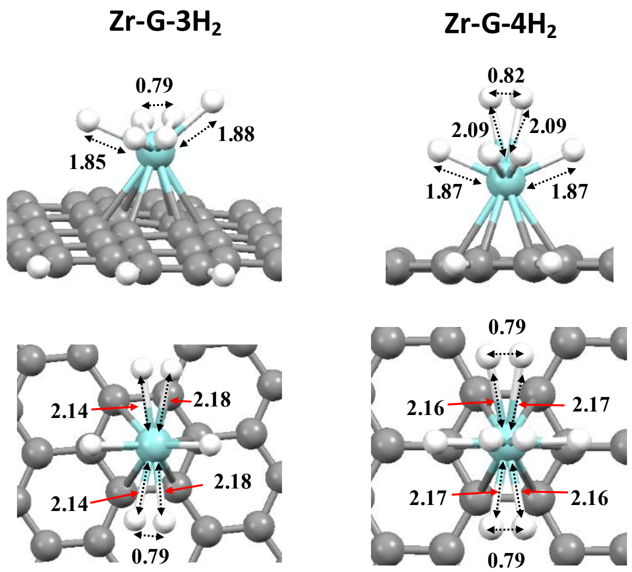

Figure S3. Optimized geometries of $3H_2$- and $4H_2$-complexes with graphene decorated with Zr. The bond distance of Zr-H and H-H are indicated.

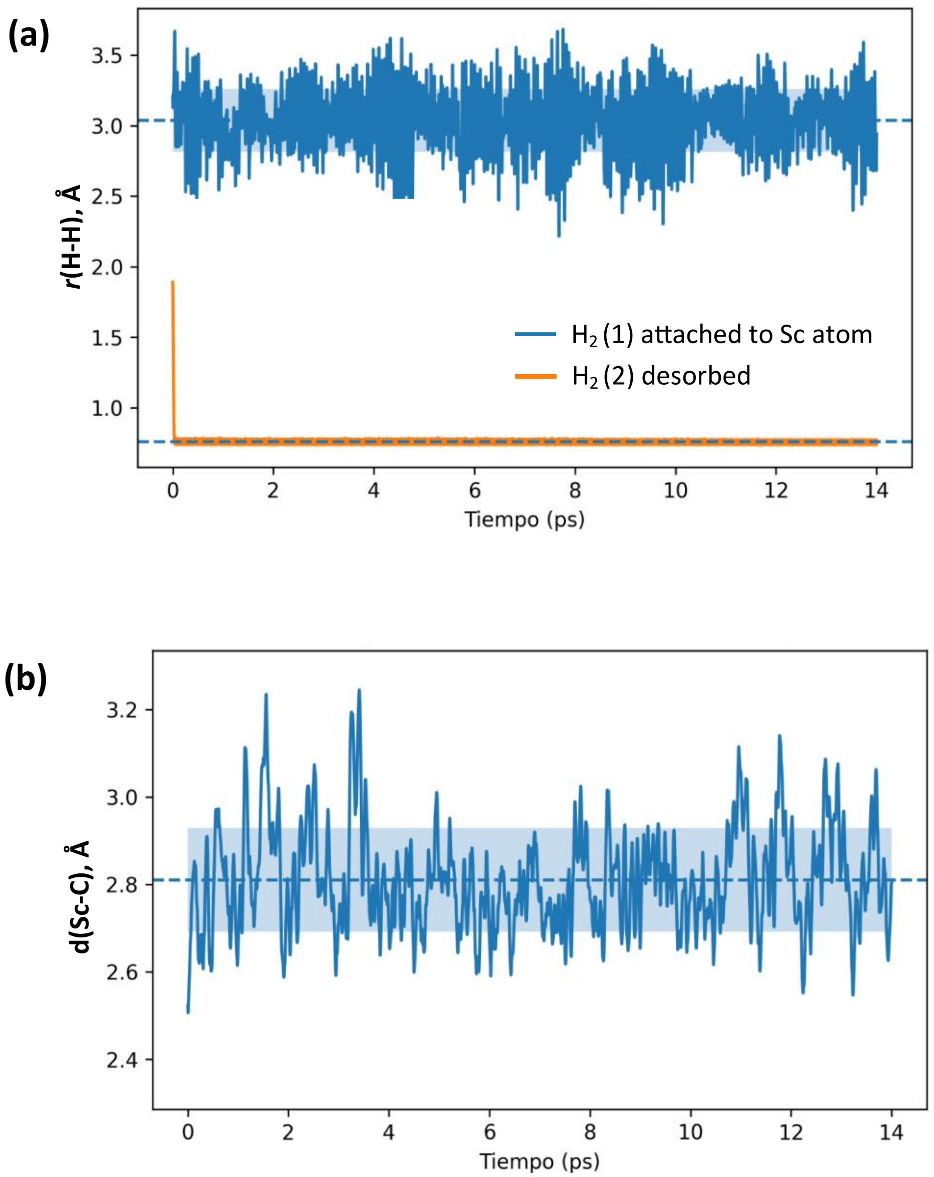

Figure S4. (a) H–H distances for two H_2 molecules adsorbed and (b) average Sc–C distances for the complex Sc-G-2H_2 as a function of time during AIMD simulations at 500 K.

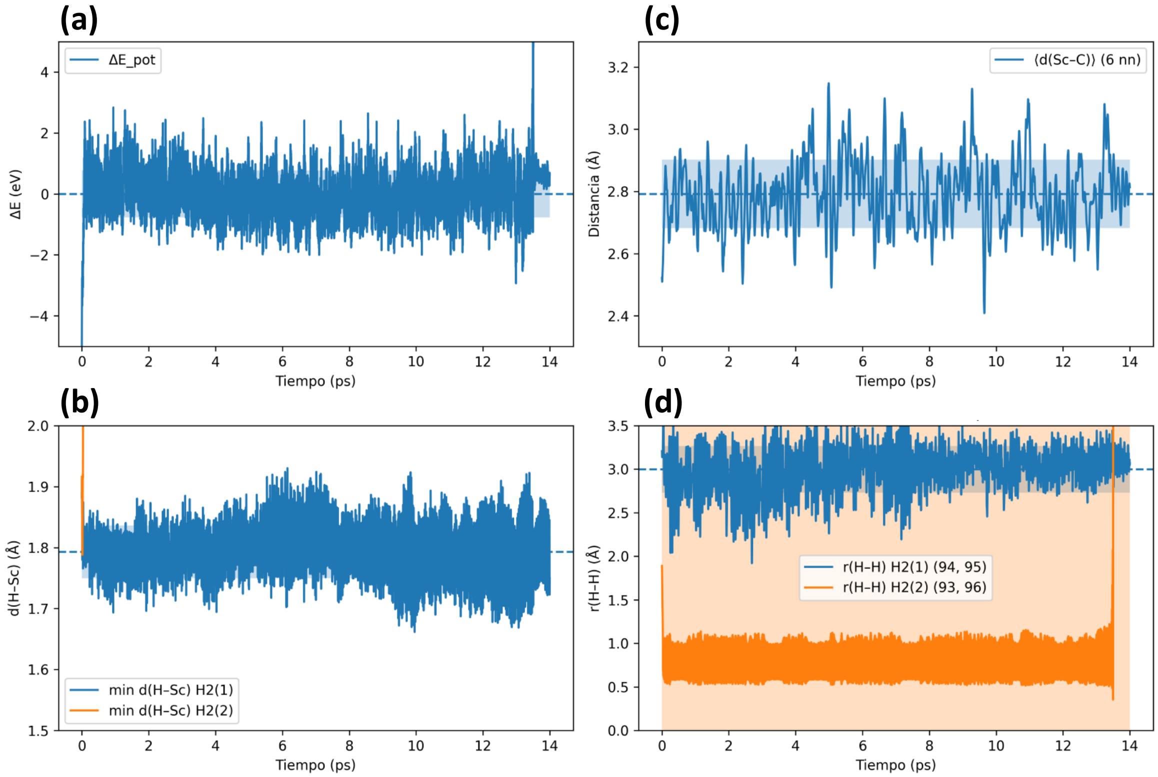

Figure S5. (a) Relative potential energy fluctuations, (b) the average H–Sc distances for the two H_2 molecules adsorbed, (c) average Sc–C distances and (d) H–H distances for two H_2 molecules adsorbed on the complex Sc-G-2H_2 as time evolution during AIMD simulations at 300 K.

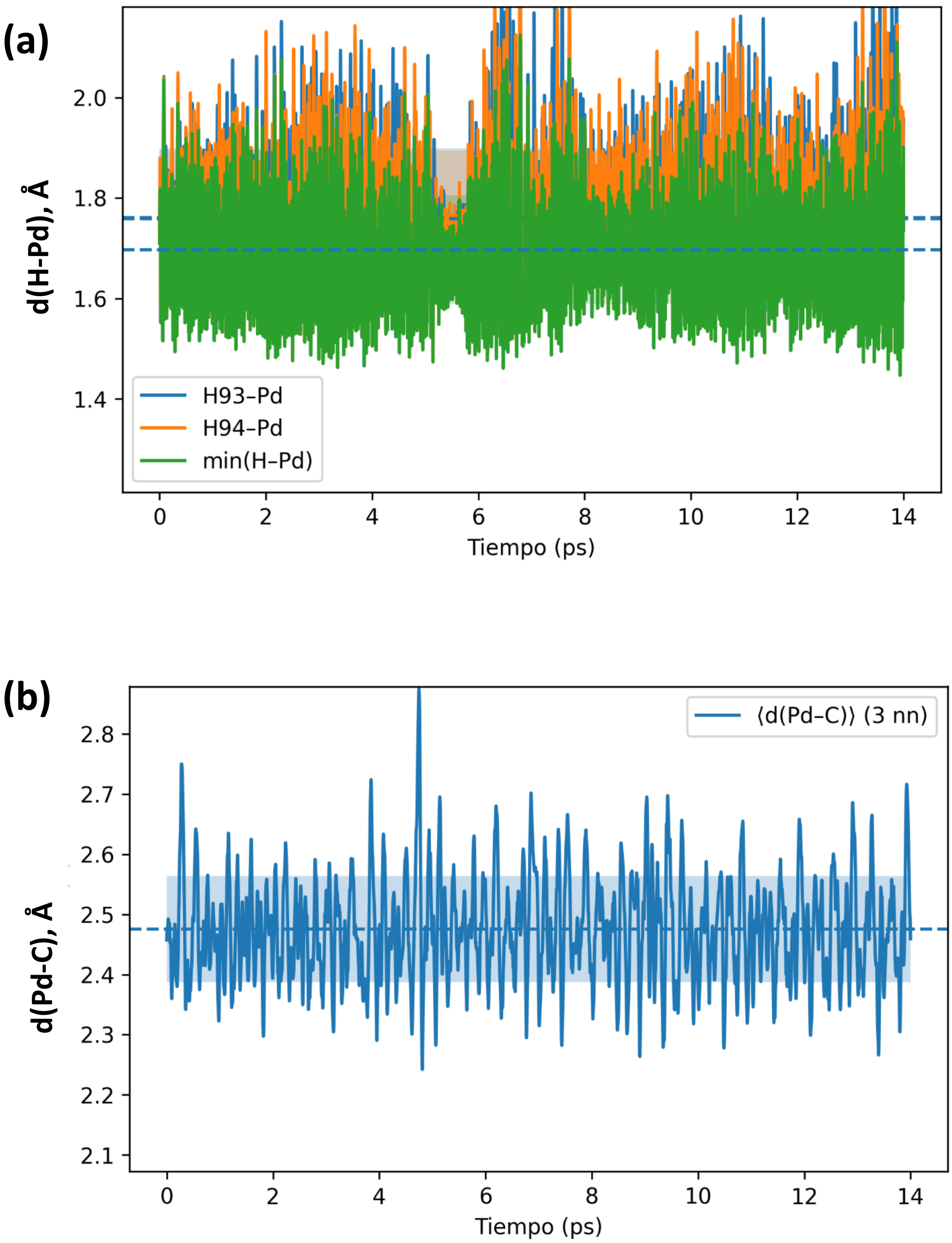

Figure S6. (a) H–Pd distances for hydrogen atoms and minimal separation on Pd-decorated graphene and (b) average Pd–C distances for the complex Pd-G-H_2 as time evolution during AIMD simulations at 500 K.

XYZ coordinates (revPBE-D4/def2-mTZVPP)

G (OSS)

C	-7.16604113794413	3.69791552227633	-0.00864837529362
C	-7.16841887736346	1.23681549794761	-0.01463296232024
C	-7.16089416745967	-1.23166359419970	-0.02839250692994
C	-7.14354196336405	-3.69258587023890	-0.04700043593327
C	-2.85469057782075	-6.16710749914147	-0.08418326967535
C	-2.90751845633799	3.72176748207818	-0.02926424770088
C	-2.90392040360810	1.25055506843748	-0.04206803593041
C	-2.89646737602168	-1.21934320845270	-0.05744653870339
C	-2.89228681520874	6.19855933278385	-0.02190655095055
C	-2.88506693874042	-3.69048662971136	-0.08095846688963
C	1.35971452515073	3.74570788903954	-0.06148554164972
C	1.36006901231649	1.26524519192873	-0.07767705184789
C	1.36757159252476	-1.20781294391616	-0.09471371496420
C	1.38227841871774	-3.68820191555569	-0.09834296832317
C	-11.44409557742504	1.20359853990776	0.01012594504469
C	5.63089436865878	1.28603003174538	-0.10105049035779
C	-11.43671627001717	-1.22466808342153	-0.00248348278996
C	5.63845673641147	-1.20250226897238	-0.11296837157029
C	-5.76544750340246	3.72392696850832	-0.01633387478700
C	-5.75071180380134	1.24352466094597	-0.03303779210992
C	-5.74323770358265	-1.22961422633500	-0.04853589530569
C	-5.74284268572590	-3.71007727536125	-0.05503892361690
C	-1.49080049039173	-6.16287688945760	-0.09277706123249
C	-1.49809630464670	3.72613250522154	-0.04670695872784
C	-1.48666037694056	1.25503086791691	-0.06518081905108
C	-1.47907549025933	-1.21500623442697	-0.06419734521680
C	-1.52836328693997	6.20273059495068	-0.02915250032553
C	-1.47553095900828	-3.68619174578965	-0.07362546197061
C	2.76043689049734	3.72829202574081	-0.07106867878377
C	2.77785829945399	1.26724264969619	-0.07736824389561
C	2.78533568368875	-1.20120952391717	-0.09058988835516
C	2.78282602827068	-3.66217636199658	-0.10826826367612
C	-10.02162067182416	1.23810341961259	-0.00021947958664
C	7.05354012104345	1.26024657762609	-0.10913958175617
C	-10.01406322575418	-1.25041526398488	-0.01324696967728
C	7.06091687142819	-1.16804522557333	-0.12073712069363
C	-5.03855405548462	2.47880329203641	-0.02391324446739
C	-5.03206049713767	0.00907025456044	-0.03372554506171
C	-5.02351232170331	-2.46057151813855	-0.04780839588455
C	-5.02924599057132	4.94412438659217	-0.01612346929208
C	-4.99923568657571	-4.92576336579383	-0.06608992475938
C	-0.77497895402218	2.49343396431538	-0.04651066288021
C	-0.76857585045455	0.02213273643027	-0.06422011364709
C	-0.76005742994530	-2.44912335051162	-0.08822762100977
C	-0.77177003722074	4.97429798503987	-0.04032592057750
C	-0.74170014935724	-4.92981030177150	-0.09107937562588
C	3.49491530883260	2.51713932329451	-0.08144301599502
C	3.49620141427928	0.03525154049137	-0.09901392554647
C	3.50996162349357	-2.44659817089226	-0.10554958627070
C	-9.30210484117171	2.45209010282417	0.00027743961660
C	-9.30035268554549	-0.00397366406174	-0.01139721183828
C	7.74650831646507	0.04820836854042	-0.11876185525301
C	-9.28712529227093	-2.45990101995668	-0.02514102843732

C	-3.62309285939965	2.48476348984304	-0.03935650601570
C	-3.61457263056949	0.01346811077483	-0.05660936226972
C	-3.60805253774225	-2.45784188934522	-0.06312813691566
C	-3.64142003977289	4.96551349553389	-0.02353938230120
C	-3.61131472335494	-4.93867041485644	-0.07445926512943
C	0.64041213084873	2.49623470313253	-0.06941279294673
C	0.64905662339245	0.02647804269857	-0.06981787920173
C	0.65545753748792	-2.44317167020208	-0.08445119498099
C	0.61612665414535	4.96136550315737	-0.04865675542172
C	0.64608050089806	-4.90836651961726	-0.10088598843409
C	4.90398770221721	2.49553340806291	-0.09104278337283
C	-12.12971574228814	-0.01265320886128	0.00901731469435
C	4.91719301243113	0.03956681002869	-0.10280970239765
C	4.91888388357962	-2.41641354618186	-0.11512151268717
C	-7.89313851809650	2.48228084487681	-0.00846764534850
C	-7.87937183503971	0.00045163233036	-0.02620233321892
C	-7.87802914722312	-2.48147176811105	-0.03454482640334
H	-3.40485239940213	-7.11125855157336	-0.08437810059163
H	-5.54446885172954	-5.87336407484368	-0.06606018263572
H	-7.68844113173886	-4.64016646534428	-0.05117981183735
H	-9.83276333134028	-3.40698287786202	-0.02757756852325
H	-11.98136790708593	-2.17141480367155	-0.00336813641535
H	-13.22181271793299	-0.01597892621238	0.01719112047871
H	-11.99456147610519	2.14694017789657	0.01903517737751
H	-9.85353661709006	3.39577829436163	0.00726158035635
H	-7.71669789060816	4.64214331840514	-0.00299675525766
H	-5.58025736738447	5.88836292657711	-0.01050313642462
H	-3.44814063178929	7.13933463635785	-0.01433207465692
H	-0.97824713366882	7.14690184001174	-0.02684441411711
H	1.16142555099545	5.90893017798100	-0.04444842788360
H	3.30531996319503	4.67587275911007	-0.06914287802171
H	5.44960145114210	3.44263175230757	-0.09124348726152
H	7.59824814945042	2.20696043595029	-0.10763364446163
H	8.83862428965497	0.05155186573316	-0.12481354524128
H	7.61130798114586	-2.11143780396223	-0.12824767699816
H	5.47020792935147	-3.36015857457130	-0.12420335305976
H	3.33340203220619	-4.60642351159083	-0.11891080639800
H	1.19704858436375	-5.85259640364871	-0.11222963004353
H	-0.93492417063647	-7.10363265055083	-0.10141036533389

G2 (OSS)

C	-7.17162010073996	3.69045240570259	-0.00840092486989
C	-7.17030386796131	1.23765380390642	-0.01508107755733
C	-7.16280954379171	-1.23253757199375	-0.02896846027089
C	-7.14919239942903	-3.68521672382299	-0.04684078287532
C	-2.85476744950818	-6.16324863495421	-0.08432153622388
C	-2.90868761776323	3.71913320156473	-0.02987709597731
C	-2.90445838737483	1.25065505461490	-0.04133440678464
C	-2.89703736190859	-1.21947195992882	-0.05779825447506
C	-2.89233596330493	6.19467541508797	-0.02200086581637
C	-2.88625544980391	-3.68786832447127	-0.08042859521323
C	1.36060203807399	3.74196691333273	-0.06172557191713
C	1.35840788367836	1.26540311844144	-0.08020677998031

C	1.36592186911214	-1.20795635958072	-0.09409430921727
C	1.38314126590705	-3.68445902872926	-0.09868209978320
C	5.60716059529066	1.29043121801711	-0.10047373786218
C	5.61476966156013	-1.20705126271067	-0.11257746941143
C	-5.76631141383940	3.72015627027573	-0.01633094198412
C	-5.74907169194014	1.24363609983149	-0.03309895001648
C	-5.74162164574840	-1.22972705418981	-0.04908655048138
C	-5.74373892492777	-3.70634221938664	-0.05485271806058
C	-1.49077490720855	-6.15901053417963	-0.09307165091361
C	-1.49695331205280	3.72350684353798	-0.04710301190958
C	-1.48615633949815	1.25513204871556	-0.06538850643345
C	-1.47858280173674	-1.21510999006478	-0.06399320709521
C	-1.52830944872333	6.19885040662604	-0.02931389870768
C	-1.47439992201346	-3.68356165660245	-0.07411812169879
C	2.76609028851316	3.72088143717806	-0.07114370467521
C	2.77974847531363	1.26811996556767	-0.07857195401644
C	2.78724953794399	-1.20207199257565	-0.09217733093081
C	2.78842434115401	-3.65476318741278	-0.10837878297610
C	-9.99789231150388	1.24266731488951	-0.00037850858060
C	-9.99027676656607	-1.25481246713403	-0.01349615725056
C	-5.04102104985922	2.47712760080274	-0.02517955578651
C	-5.03128512175420	0.00905886125841	-0.03267471463819
C	-5.02602715192524	-2.45891802340431	-0.04872515948569
C	-5.03138037409044	4.93998926481650	-0.01625211214110
C	-5.00139435890236	-4.92166227795744	-0.06591279816315
C	-0.77670738339707	2.49219519238903	-0.04691876898393
C	-0.76844873003320	0.02212801658070	-0.06585905752353
C	-0.76177417221423	-2.44790220350200	-0.08800105549694
C	-0.77037012025967	4.97091380458306	-0.04059826016060
C	-0.74032191840632	-4.92642538566331	-0.09131658374916
C	3.49037451116957	2.51453476440559	-0.08119565822409
C	3.49619201664588	0.03523831817451	-0.09803785453755
C	3.50541648621405	-2.44403510807206	-0.10542286125225
C	-9.31862836650144	2.43947449457343	0.00068254552355
C	-9.30587841070977	-0.00395349108697	-0.01213420170067
C	-9.30377643952094	-2.44743411558994	-0.02479093340504
C	-3.62141192717441	2.48353401893747	-0.03918602308889
C	-3.61471682636719	0.01346550859887	-0.05667174541622
C	-3.60639975568472	-2.45662774643297	-0.06280450505987
C	-3.64282571330977	4.96211739566968	-0.02343580837810
C	-3.61274340267263	-4.93530422756587	-0.07458516534736
C	0.64289421794477	2.49457378943921	-0.06724364461659
C	0.64831341667829	0.02647852341912	-0.06799652918413
C	0.65790624791140	-2.44150491934311	-0.08582212270059
C	0.61826451302876	4.95726600972905	-0.04918286845469
C	0.64818835655377	-4.90424791848291	-0.10130008202747
C	4.92066844980078	2.48306129555932	-0.09062502177474
C	4.92274069891366	0.03958118643577	-0.10326163411827
C	4.93550128673745	-2.40384839510893	-0.11470742162424
C	-7.88854395918931	2.47966625716535	-0.00817188355466

C	-7.87936160059945	0.00043649178159	-0.02594929755590
C	-7.87347595283911	-2.47888817479515	-0.03435877369979
H	-3.40445001561993	-7.10772472845557	-0.08460187493751
H	-5.54713680472343	-5.86901763040295	-0.06583992575679
H	-7.69547153726344	-4.63216585631822	-0.05053062273431
H	-9.84725800342160	-3.39528113056565	-0.02648481129631
H	-9.86778218726641	3.38401386293459	0.00857414342743
H	-7.72362838120652	4.63405326196746	-0.00215975591704
H	-5.58288009040238	5.88398763521897	-0.01017512257294
H	-3.44770220404135	7.13578484167979	-0.01431501019598
H	-0.97866865471726	7.14334566812286	-0.02706697164758
H	1.16405431338200	5.90459238605368	-0.04547285979570
H	3.31236157694957	4.66782751310535	-0.06975148951963
H	5.46413296535021	3.43091886443134	-0.08980646073158
H	5.48465472576143	-3.34839079907766	-0.12282704581667
H	3.34038056118882	-4.59837603706038	-0.11823477224946
H	1.19964961912259	-5.84823921748664	-0.11241858072347
H	-0.93537419609481	-7.10009468980029	-0.10172056549696
H	6.69995816386381	1.28751780482012	-0.10666215124481
H	6.70752568963335	-1.19737261311586	-0.11869353456554
H	-11.09063078167406	1.23302722952461	0.00769760977930
H	-11.08306055421097	-1.25190772244240	-0.00535467771201

Sc-G

C	-7.1976376	3.6770811	0.0738907
C	-7.1946273	1.2163138	0.0223910
C	-7.1873884	-1.2566019	-0.0193579
C	-7.1756820	-3.7175480	-0.0516312
C	-2.8701490	-6.1861888	-0.1060612
C	-2.9286789	3.7033474	0.0241499
C	-2.9288221	1.2350751	-0.0791885
C	-2.9214052	-1.2465240	-0.1252925
C	-2.9063866	6.1713238	0.1017782
C	-2.9067814	-3.7171907	-0.1168354
C	1.3553453	3.7289410	0.0262552
C	1.3586575	1.2462928	-0.0435525
C	1.3658645	-1.2344401	-0.0810628
C	1.3775800	-3.7181997	-0.0956076
C	-11.4693701	1.1797865	0.1035642
C	5.6271461	1.2618995	0.0241688
C	-11.4622102	-1.2479755	0.0626571
C	5.6345174	-1.2267661	-0.0156245
C	-5.7892956	3.7079423	0.0501451
C	-5.7778249	1.2250765	-0.0191964
C	-5.7706264	-1.2555653	-0.0647326
C	-5.7671543	-3.7391644	-0.0764273
C	-1.5051896	-6.1822855	-0.1092194
C	-1.5051449	3.7078992	0.0037559
C	-1.4909070	1.2388325	-0.0861457
C	-1.4834683	-1.2427927	-0.1244368
C	-1.5414387	6.1752167	0.0964188
C	-1.4831868	-3.7131282	-0.1045221

C	2.7640062	3.7067524	0.0399525
C	2.7756807	1.2460961	-0.0086101
C	2.7829213	-1.2268335	-0.0488581
C	2.7860557	-3.6879438	-0.0799427
C	-10.0458709	1.2148490	0.0759461
C	7.0509639	1.2353781	0.0421803
C	-10.0385486	-1.2737628	0.0338496
C	7.0581825	-1.1924272	0.0032151
C	-5.0654083	2.4646593	-0.0029802
C	-5.0636971	-0.0129206	-0.0578312
C	-5.0505676	-2.4906105	-0.0873618
C	-5.0522977	4.9212540	0.0793535
C	-5.0231649	-4.9485073	-0.0876463
C	-0.7803803	2.4839607	-0.0224805
C	-0.7688051	0.0011223	-0.1518182
C	-0.7652576	-2.4851180	-0.1146257
C	-0.7825363	4.9497002	0.0540605
C	-0.7532784	-4.9517434	-0.1085083
C	3.4923703	2.4989877	0.0215100
C	3.4938018	0.0117631	-0.0316573
C	3.5070986	-2.4758197	-0.0586065
C	-9.3318427	2.4288989	0.0851179
C	-9.3255882	-0.0271076	0.0399843
C	7.7438233	0.0233737	0.0312826
C	-9.3173412	-2.4832234	0.0021654
C	-3.6468075	2.4763204	-0.0233381
C	-3.6437220	-0.0073732	-0.1452128
C	-3.6316414	-2.4930928	-0.0986516
C	-3.6584027	4.9413571	0.0659026
C	-3.6292403	-4.9600201	-0.1029870
C	0.6385813	2.4813131	-0.0204268
C	0.6516295	0.0040708	-0.0732003
C	0.6534264	-2.4739332	-0.1022053
C	0.6114506	4.9378975	0.0597654
C	0.6406833	-4.9319118	-0.1027784
C	4.9058578	2.4716430	0.0374696
C	-12.1549686	-0.0363304	0.0963840
C	4.9141258	0.0156094	-0.0056314
C	4.9204250	-2.4405488	-0.0415991
C	-7.9186724	2.4648596	0.0594585
C	-7.9054808	-0.0222869	0.0037285
C	-7.9040008	-2.5098954	-0.0251075
H	-3.4189872	-7.1312101	-0.1043554
H	-5.5646804	-5.8977811	-0.0777334
H	-7.7208648	-4.6644118	-0.0498733
H	-9.8643518	-3.4291787	-0.0018159
H	-12.0060197	-2.1949581	0.0577663
H	-13.2468926	-0.0399143	0.1177369
H	-12.0186954	2.1231929	0.1304239
H	-9.8844046	3.3712109	0.1127363
H	-7.7485214	4.6200433	0.1072988
H	-5.5993794	5.8666494	0.1163058
H	-3.4605357	7.1126905	0.1341597
H	-0.9924324	7.1196655	0.1261294
H	1.1531179	5.8864092	0.0968810
H	3.3092602	4.6531548	0.0683424
H	5.4528837	3.4173196	0.0605949
H	7.5947968	2.1820968	0.0646196

H	8.8358696	0.0264019	0.0451585
H	7.6076158	-2.1361253	-0.0046685
H	5.4730901	-3.3831782	-0.0489781
H	3.3370799	-4.6314143	-0.0817779
H	1.1879158	-5.8779415	-0.1008237
H	-0.9509458	-7.1241593	-0.1118640
Sc	-2.2027552	-0.0378814	1.9160188

Y-G

C	-7.18301716431368	3.69990909670528	0.00155702050395
C	-7.18165948405217	1.23905048280352	-0.00695919133093
C	-7.17454668113801	-1.23372528780669	-0.02306682151989
C	-7.16193347341527	-3.69470653846270	-0.04616900791859
C	-2.85642233747953	-6.16367838957268	-0.11476597853511
C	-2.91400181855785	3.72616840886608	-0.06998427465910
C	-2.91336167688612	1.25647223083613	-0.10917215599952
C	-2.90651246238577	-1.22518634662563	-0.12017739437384
C	-2.89300432637791	6.19535382279790	-0.04895469402082
C	-2.89272771323950	-3.69491221430177	-0.10943721680445
C	1.36906507575195	3.75109891039286	-0.08955824690042
C	1.37300612906952	1.26920638050238	-0.10993414041574
C	1.38048619638890	-1.21105733806147	-0.11857460576316
C	1.39079808599370	-3.69322126019019	-0.11814653748999
C	-11.45639849404279	1.20393608787201	0.07874846278941
C	5.64244206867774	1.28600968053632	-0.06666713722688
C	-11.44931584664302	-1.22432124654129	0.06235562087229
C	5.64986824648823	-1.20311941154032	-0.07508278013791
C	-5.77501412938772	3.72905742581418	-0.02678528395430
C	-5.76459550477664	1.24705197649106	-0.05030756142736
C	-5.75727428073061	-1.23325014907715	-0.06619411657206
C	-5.75352966383253	-3.71557640384196	-0.07458275402809
C	-1.49136526577471	-6.15931030138945	-0.12173166022038
C	-1.49268486789228	3.73104764009630	-0.09449180697144
C	-1.47814626286744	1.26192438008714	-0.13814761483409
C	-1.47126934115921	-1.22070152290707	-0.15630761010365
C	-1.52804315175610	6.19967373871260	-0.06054204379342
C	-1.47096347800339	-3.69047803944256	-0.13073363852510
C	2.77773962194452	3.73049221412273	-0.08112257151501
C	2.79092586925798	1.26943569888588	-0.08787640767621
C	2.79801043495190	-1.20323103999550	-0.09692697780860
C	2.79943683481132	-3.66408410816196	-0.10850493417961
C	-10.03305392736510	1.23870881146703	0.04830706500498
C	7.06653316933019	1.25961886891077	-0.05454241408530
C	-10.02574432455018	-1.25045035068455	0.03158209427706
C	7.07341616871989	-1.16871777392216	-0.06266961686398
C	-5.05135099554934	2.48580527210537	-0.05733559487725
C	-5.05120642159480	0.00910229527378	-0.07272233384366
C	-5.03720345977281	-2.46773249437086	-0.09069188920096
C	-5.03771381020239	4.94319582753552	-0.02899964257102
C	-5.00921949016008	-4.92503204227605	-0.08862729157881
C	-0.76661543639023	2.50617480437320	-0.10121232708890
C	-0.75557723165315	0.02266020489446	-0.18505330459180
C	-0.75258023079065	-2.46104451825244	-0.12576075792939
C	-0.76910590003961	4.97394573243872	-0.07748196521730
C	-0.74001813316561	-4.92887231620957	-0.12409926371988
C	3.50726096436163	2.52260059051676	-0.07962981224272

C	3.50861587649760	0.03523567892782	-0.09273626712475
C	3.52178873354011	-2.45209796045977	-0.09757082973912
C	-9.31828824769386	2.45307859899619	0.04037800166260
C	-9.31320275142730	-0.00369255508745	0.02507283562322
C	7.75915182780209	0.04758044918477	-0.05266936975642
C	-9.30410136045506	-2.46033113610850	0.00746442635301
C	-3.63227894445972	2.49694140076766	-0.08019507520217
C	-3.63048066509816	0.01388323933466	-0.14472476149131
C	-3.61837556409144	-2.47030164878501	-0.10717604114826
C	-3.64457003200957	4.96496662428974	-0.05093653845603
C	-3.61565381967226	-4.93811003772659	-0.10629486516530
C	0.65288896644750	2.50364796671425	-0.10631995598538
C	0.66651304598497	0.02696514701888	-0.11675617171165
C	0.66722067915516	-2.44983453935886	-0.12487123246906
C	0.62431359941187	4.96062762305650	-0.07882252347428
C	0.65351670580848	-4.90721750420680	-0.12039580541851
C	4.92071462414697	2.49598158541575	-0.06897878035411
C	-12.14188545408575	-0.01239123372315	0.08532753453299
C	4.92995319954326	0.03935510430490	-0.07659482505377
C	4.93499165818757	-2.41739455038783	-0.08575625859217
C	-7.90554142523101	2.48773170690927	0.01095865706517
C	-7.89230365967904	0.00056145417367	-0.01196029785966
C	-7.89105787280093	-2.48682511006414	-0.02165510734974
H	-3.40489275347004	-7.10882637345964	-0.11377286968364
H	-5.55160669703865	-5.87405778695171	-0.08169447115366
H	-7.70663409712677	-4.64196297040005	-0.04121794119744
H	-9.85137065121093	-3.40628977190832	0.01010274075851
H	-11.99384487348817	-2.17115958702127	0.06730314454041
H	-13.23388482899043	-0.01574391180032	0.10843508857833
H	-12.00646767881223	2.14740244458097	0.09640377461585
H	-9.87088780495428	3.39578827730205	0.05593104517433
H	-7.73301620525269	4.64397308917552	0.01832656084100
H	-5.58584124791767	5.88877285717795	-0.01075873033883
H	-3.44744361721303	7.13692179057402	-0.03623644664232
H	-0.97939838741092	7.14470222455568	-0.05627912313670
H	1.16677655205185	5.90957368168856	-0.06682685514921
H	3.32232210177385	4.67778329013025	-0.07317212550812
H	5.46801573415634	3.44188770573296	-0.06276553839997
H	7.61115462620517	2.20637584240340	-0.04713902222650
H	8.85141075494997	0.05067318573615	-0.04365949042938
H	7.62353268371846	-2.11231638740566	-0.06163479645015
H	5.48776215374102	-3.36013749335569	-0.08577094645903
H	3.34959168258666	-4.60819365692176	-0.10771710607325
H	1.20169616359680	-5.85291894396606	-0.11656053633873
H	-0.93684386756850	-7.10090946174845	-0.12518442146953
Y	-2.13957535194678	0.00251770329156	2.11924294233169

Zr-G

C	-7.17260372266947	3.70268324720041	-0.00674259472792
C	-7.18076464879680	1.23856965005683	-0.03696362522247
C	-7.17343403418185	-1.23323453291936	-0.04844467638833
C	-7.14989051591794	-3.69732109247739	-0.04252484666781
C	-2.85421607891796	-6.17341430333694	-0.03986103883731
C	-2.90848313620269	3.73029209391473	-0.02307557030217
C	-2.91806378430337	1.25837349012605	-0.09521393549928
C	-2.91041326728269	-1.22628387076301	-0.11634073232448

C	-2.89191592340995	6.20469308997382	0.02300784798276
C	-2.88526856737596	-3.69904410629717	-0.07560903123024
C	1.36930713623074	3.75263029660697	-0.06088989520762
C	1.37531040782032	1.26805893392496	-0.11808425889875
C	1.38268399340227	-1.21010738064487	-0.12838268986470
C	1.39213111258931	-3.69542472961947	-0.10041403391999
C	-11.45467001884045	1.20536317086080	0.01702582738659
C	5.64531055903826	1.28823513444770	-0.10066183194527
C	-11.44721998311279	-1.22644198882074	0.00558381085036
C	5.65270095399978	-1.20449066886899	-0.11403799308616
C	-5.77516664288023	3.73107415890004	-0.01716365617215
C	-5.76626088169705	1.24625889637627	-0.07099037200505
C	-5.75892000852697	-1.23183999662733	-0.08618680738298
C	-5.75227375102303	-3.71688514277734	-0.05410199435053
C	-1.49057030049043	-6.16945378859971	-0.04965504572494
C	-1.49775240216979	3.73479337251937	-0.04607061762104
C	-1.47321833401367	1.26254939131390	-0.11077223850021
C	-1.46547381514464	-1.22211322379672	-0.11606570522077
C	-1.52825353720484	6.20865968471890	0.01547576941127
C	-1.47436501770109	-3.69473549648326	-0.07049387317519
C	2.76693149750560	3.73303272924852	-0.06766644303221
C	2.79022964063084	1.26916526837687	-0.09872274345528
C	2.79747634840129	-1.20266454310283	-0.11236854140846
C	2.78954207648684	-3.66702617021120	-0.10745283843368
C	-10.03586970562228	1.24021388455465	-0.00154088988770
C	7.06446462236420	1.26189401698621	-0.10055150155549
C	-10.02825071543945	-1.25255005931671	-0.01338108355222
C	7.07151038621872	-1.16982305526215	-0.11365661971308
C	-5.04556929140804	2.48768419774002	-0.05495414746448
C	-5.05834127132339	0.00938086951332	-0.09372775254557
C	-5.03039796428038	-2.46875204791894	-0.08095102661198
C	-5.03304067468943	4.95099429419111	0.00697410003527
C	-5.00250254710389	-4.93230095303549	-0.04162316444160
C	-0.76042736685056	2.50634702748149	-0.06557198973047
C	-0.75241450469045	0.02333999848173	-0.17799148441323
C	-0.74490532025249	-2.46212997596685	-0.09731634057736
C	-0.76856226686359	4.98086139833060	-0.01646057239422
C	-0.73797909383543	-4.93703153247627	-0.06838378912732
C	3.51007028906447	2.52062508774454	-0.08696585361632
C	3.50791494936768	0.03551336374724	-0.11948371496282
C	3.52502527244903	-2.44982149431336	-0.11353217672726
C	-9.31008932693297	2.45607381224621	-0.00080511953420
C	-9.31430267862137	-0.00392082053926	-0.01911389353585
C	7.75429991500038	0.04805426045420	-0.10773168684297
C	-9.29512209384703	-2.46395167920672	-0.02405017040372
C	-3.63830578920168	2.49799345484632	-0.05408283198295
C	-3.63179680267069	0.01364165172126	-0.16122557693051
C	-3.62273289847330	-2.47047941453189	-0.07382497731379
C	-3.64474419071349	4.97246132167131	0.00097159578994
C	-3.61414868650726	-4.94538665537001	-0.04951707876649
C	0.64700229161644	2.50474862678990	-0.09090244003786
C	0.67467659227513	0.02693553581192	-0.12954886965743
C	0.66215410563630	-2.45170714197212	-0.11603346296695
C	0.61979903815490	4.96778014093071	-0.02650464763397
C	0.65029645504419	-4.91557599728864	-0.07961226320424
C	4.91218829173759	2.49964082120747	-0.08957106384634
C	-12.13706195434077	-0.01268249052619	0.01964421443164
C	4.93116459411353	0.03973823922908	-0.10968551800321

C	4.92692535748576	-2.42038080278869	-0.11619791951237
C	-7.90820252735944	2.48559784903393	-0.01593313485429
C	-7.89132073630788	0.00051346003404	-0.04725902575330
C	-7.89309673210756	-2.48483518711679	-0.03998591790918
H	-3.40355885651038	-7.11802196594266	-0.02674609848061
H	-5.54629977223141	-5.88061118537228	-0.02360467875384
H	-7.69519478919527	-4.64480321160313	-0.03145874023150
H	-9.84140084000327	-3.41067748804981	-0.01935575759514
H	-11.99330186298487	-2.17226265687784	0.00871283283830
H	-13.22948177936475	-0.01615598448335	0.03341668570957
H	-12.00638408546170	2.14783782166678	0.02907598206878
H	-9.86205694116655	3.39940561133024	0.01278975355925
H	-7.72386303091431	4.64656112131128	0.01298536681372
H	-5.58269696443561	5.89579946537430	0.02918666670126
H	-3.44673404715482	7.14595644221207	0.04364050043901
H	-0.97873183969814	7.15311739188848	0.03143338935979
H	1.16384446172733	5.91589159159804	-0.00582619267764
H	3.31238628766676	4.68039416693526	-0.05412732584286
H	5.45855122110587	3.44630886307468	-0.08248908731726
H	7.61070833906709	2.20761774318297	-0.09507835838188
H	8.84681814348649	0.05129545505628	-0.10832054646159
H	7.62311226752348	-2.11244196035575	-0.11831535405722
H	5.47890894364428	-3.36379229759589	-0.11935980399870
H	3.34094858987104	-4.61102457023883	-0.10453485313952
H	1.20012094088021	-5.86053056583584	-0.07411435606232
H	-0.93561512462490	-7.11085748824730	-0.04592563257822
Zr	-2.18451604655291	0.00584566263537	1.85492828881657

Pd-G

C	-7.15227879284890	3.70050002143079	-0.01450044713754
C	-7.15660684045877	1.23925486610475	-0.00416115495463
C	-7.14922049076093	-1.23354530798706	-0.01692298660728
C	-7.13021655437270	-3.69449601567818	-0.05270305280637
C	-2.83811975521620	-6.16922236773904	-0.09659350580714
C	-2.89078218300739	3.72518214742669	-0.01859305788574
C	-2.88125230170141	1.26092318664544	-0.01002316328070
C	-2.87387476133206	-1.22978398276093	-0.02400381609188
C	-2.87502976997217	6.20101291800355	-0.03175475444759
C	-2.86865783431938	-3.69376735182464	-0.05927760965124
C	1.37880218788367	3.74725298017692	-0.06742259381700
C	1.37833217466796	1.26597584633615	-0.07913152970318
C	1.38580340299402	-1.20881876597664	-0.08239642753838
C	1.40092373563336	-3.68989383787230	-0.10642383353059
C	-11.43041666754351	1.20426319367268	0.00031211040010
C	5.64863181794622	1.28577487716231	-0.12756270193965
C	-11.42317013636239	-1.22396145775294	-0.01217022401115
C	5.65608552295472	-1.20276510744191	-0.14088430097747
C	-5.75076698494053	3.72557234725547	-0.02298296276672
C	-5.74082162705982	1.24834994956786	-0.01324648250389
C	-5.73341729649361	-1.23414107831677	-0.02632740162489
C	-5.72857716304356	-3.71114734465756	-0.06127619756486
C	-1.47421107223328	-6.16403770604527	-0.10176837473883
C	-1.48048494446845	3.72810117987642	-0.04521901496401
C	-1.46454032574723	1.25778903809672	-0.03772116829335
C	-1.45725979384957	-1.21776085643058	-0.05527387890239
C	-1.51112500396702	6.20406062690123	-0.03758320297535

C	-1.45832589595558	-3.68800082173378	-0.08198961543432
C	2.77934755337523	3.72913378738540	-0.08299544010105
C	2.79564625674614	1.26745951949646	-0.10055456763047
C	2.80301446144301	-1.20163690166359	-0.11282119932918
C	2.80133757321450	-3.66327718185176	-0.12070133165008
C	-10.00823683462114	1.23903962573539	-0.00460721553881
C	7.07105875818170	1.25988397807829	-0.14545486910723
C	-10.00080274691984	-1.25026248605676	-0.01740371626635
C	7.07834845322375	-1.16823667385918	-0.15853907079770
C	-5.02431376940413	2.48213023912056	-0.02409290835810
C	-5.02927352634470	0.00910796686289	0.00180951617358
C	-5.00957759974820	-2.46347062770516	-0.04942989258861
C	-5.01336026365800	4.94627245327829	-0.02467202284484
C	-4.98388005331611	-4.92736781514063	-0.07610088151539
C	-0.75563110083576	2.49583508572785	-0.05395225607816
C	-0.74957068202252	0.02216929918637	-0.06075028997252
C	-0.74078396806825	-2.45154093984441	-0.06466510524551
C	-0.75386117416000	4.97540788981098	-0.04377301455742
C	-0.72432368466325	-4.93091469201022	-0.09444328803108
C	3.51310691268181	2.51739324431721	-0.09725289237828
C	3.51420115977098	0.03501599556695	-0.10294426808309
C	3.52787711003476	-2.44714929620194	-0.12313130407662
C	-9.28965298834805	2.45373820334018	-0.00605858254277
C	-9.28667969479593	-0.00347505648271	-0.01096593488372
C	7.76401077432151	0.04793239214515	-0.16064441592921
C	-9.27498497951245	-2.46058612399714	-0.03127671172986
C	-3.60792012998520	2.49104118769315	-0.02494679147619
C	-3.59639543304781	0.01314286863356	0.03933087673897
C	-3.59314682803596	-2.46399996970265	-0.04907249371742
C	-3.62555261073759	4.96854596898127	-0.02863434609849
C	-3.59596728090490	-4.94136453321843	-0.08005105043477
C	0.65995037139038	2.49777827977746	-0.05237532243859
C	0.66641539476495	0.02643061906093	-0.08141953683835
C	0.67442450801393	-2.44484788011533	-0.09375391268839
C	0.63425756123528	4.96288363417688	-0.05694202009296
C	0.66367752030982	-4.90997449538339	-0.10645255600094
C	4.92208620292996	2.49538789143521	-0.11250842810398
C	-12.11612209953869	-0.01191537622848	-0.00287205887958
C	4.93469988245582	0.03931403762333	-0.12518995876234
C	4.93670136324498	-2.41670089245019	-0.13829818519253
C	-7.88090470334150	2.48536635507221	-0.01043813013487
C	-7.86645396990736	0.00077322878484	-0.01827033664192
C	-7.86606970373944	-2.48381833639914	-0.03595387199697
H	-3.38751615410278	-7.11382425040030	-0.10590466728868
H	-5.52979093887550	-5.87455421048746	-0.08737543285115
H	-7.67479096629062	-4.64216522834703	-0.06220305894558
H	-9.82204851029291	-3.40683361412390	-0.04029816116472
H	-11.96810668991382	-2.17058076027976	-0.01649636250701
H	-13.20826637946481	-0.01518694549745	0.00086769882569
H	-11.98092165176751	2.14764925379656	0.00569059768963
H	-9.84231524721337	3.39676868072048	-0.00538017072695
H	-7.70247350195152	4.64496274882240	-0.01407588810554
H	-5.56487182327715	5.89026572346682	-0.02653911417842
H	-3.43001599851550	7.14237272909486	-0.03103089819386
H	-0.96064120706202	7.14806796385167	-0.03975224413975
H	1.17953558264693	5.91044679217289	-0.06137160808241
H	3.32534980717131	4.67606937276350	-0.08792299629415
H	5.46809616991458	3.44227781486433	-0.11307728961399

H	7.61576230211150	2.20663561311452	-0.14754242163510
H	8.85607426361246	0.05126412726833	-0.17459989711342
H	7.62868634097008	-2.11164495972668	-0.17080248258478
H	5.48828474552192	-3.36030153106360	-0.14905239609948
H	3.35301597455994	-4.60689962384148	-0.13052047968891
H	1.21458040927791	-5.85424150964010	-0.11759489783309
H	-0.91809042496178	-7.10466287461374	-0.11419711161067
Pd	-3.66935915020326	-0.00169342133392	2.38301092752018

Nb-G

C	-7.17710242313543	3.69948327877144	-0.04714731219913
C	-7.17435486687172	1.23373299002288	-0.02791886371606
C	-7.16701233076046	-1.24023233675429	-0.00911195932958
C	-7.14710327754265	-3.70305021653693	-0.01195735317576
C	-2.85264255132858	-6.17624834854483	-0.08723138927758
C	-2.91034706244216	3.72536147788359	-0.03399660147632
C	-2.91251314331314	1.25587477099721	-0.05086637047766
C	-2.89522040627378	-1.23622632441877	-0.04555397576698
C	-2.88983649734897	6.19686209375303	-0.02824286190912
C	-2.88234115315505	-3.70164001653615	-0.06041626591826
C	1.37026598543092	3.74515823586306	-0.02495760572977
C	1.37152599148886	1.26077378610410	-0.05554049065120
C	1.37939546097802	-1.21879572810014	-0.09794227844087
C	1.39082541301600	-3.69845916612686	-0.13927676525780
C	-11.44725099804607	1.19392219173516	-0.02033359342780
C	5.63943369728887	1.27876070684328	-0.09735811621138
C	-11.43960294632654	-1.23475384487186	-0.00362203523108
C	5.64674197927424	-1.21048954771046	-0.14065521270779
C	-5.77587094677019	3.72760104463504	-0.04758787482423
C	-5.75940740134517	1.24030068111973	-0.04172619934739
C	-5.74961889344189	-1.23758847894726	-0.02553530979258
C	-5.74630186302591	-3.71853561509709	-0.02350420066132
C	-1.48893555026388	-6.17239614875491	-0.11312309502806
C	-1.49289191276838	3.72660180951660	-0.02417462595659
C	-1.47621510560925	1.25225962609847	-0.00475841982902
C	-1.46979470709834	-1.23298735930342	-0.08656658235755
C	-1.52586560594780	6.19959532137332	-0.01630460988275
C	-1.47272574134849	-3.69811454095715	-0.10382794310408
C	2.77316689982827	3.72540011013902	-0.03530404684843
C	2.78671006172928	1.26245751961807	-0.07827007995999
C	2.79625561308025	-1.21059110695848	-0.12128487456190
C	2.79261148606996	-3.67291389780666	-0.15168830670822
C	-10.02572690220867	1.23038340212049	-0.02448547432798
C	7.06108634525574	1.25176439748886	-0.11105088341722
C	-10.01694793840391	-1.25987193378039	-0.00734004674063
C	7.06868005563590	-1.17638297799325	-0.15327323298040
C	-5.04784128419994	2.48299014731867	-0.04604681903018
C	-5.04652157332296	0.00339433388476	-0.01586595505070
C	-5.02715167622445	-2.46992601749020	-0.02150678548093
C	-5.03362265545064	4.94432520928920	-0.04541553034654
C	-4.99811058661066	-4.93331470392015	-0.03971491731778
C	-0.76282216049970	2.50154490921486	-0.00999624167356
C	-0.75128069955687	0.00801706277870	-0.06884889510416
C	-0.75236223056315	-2.46349686618231	-0.09929217977794
C	-0.76690661665467	4.97155399517467	-0.01362077195363
C	-0.73787967373634	-4.93989383092663	-0.11891730732326

C	3.50531948574673	2.51432372340836	-0.05854477306244
C	3.50582845162329	0.02728352781067	-0.09610009554407
C	3.52012075721714	-2.45749702808517	-0.14293620413941
C	-9.30907270195497	2.44563960684190	-0.03626985174942
C	-9.30437783917123	-0.01228075452610	-0.01687578217731
C	7.75400839168870	0.03964837781850	-0.13852335900101
C	-9.29162003336739	-2.46932572306853	-0.00439563481975
C	-3.63583068160647	2.49896955880248	-0.04707902211979
C	-3.62234787160916	0.00450974396072	-0.00688526366934
C	-3.61002777175542	-2.47174120685081	-0.04950461595191
C	-3.64333042729386	4.96561021548457	-0.03883655635128
C	-3.61047637271701	-4.94811373453295	-0.06266703606696
C	0.65112643177592	2.49626111134611	-0.03207325258443
C	0.66513055873913	0.01807684330557	-0.08371158883347
C	0.66585442640655	-2.45418696913205	-0.12169907278965
C	0.62342754003981	4.95782345924687	-0.01075920228707
C	0.65042943806876	-4.91759280292496	-0.14038106487432
C	4.91449081294979	2.48904526293538	-0.07042255829453
C	-12.13246130258083	-0.02313919858511	-0.00980752694187
C	4.92555145946287	0.03177877261317	-0.11257686868554
C	4.92876046838808	-2.42506076688347	-0.15432917931134
C	-7.90111020598346	2.48185124104334	-0.03946957702639
C	-7.88514057854136	-0.00662735368447	-0.02423727558973
C	-7.88295068541100	-2.49233915546360	-0.00837800251846
H	-3.40275976927471	-7.12045915087877	-0.08554440616906
H	-5.54337227521771	-5.88099941270819	-0.04102268085728
H	-7.69116360922442	-4.65090623740058	-0.01232994519842
H	-9.83822838472581	-3.41585247533754	-0.00166823325959
H	-11.98401665836119	-2.18165587588610	0.00345620789547
H	-13.22466490875495	-0.02653634335109	-0.00701705409607
H	-11.99895059021787	2.13664907051522	-0.02593701455375
H	-9.86374969298627	3.38742497226765	-0.04323066918570
H	-7.73005352633632	4.64202004404885	-0.05063601820072
H	-5.58203390623729	5.89003557492202	-0.04694787885367
H	-3.44353486473259	7.13894101277084	-0.03107022380931
H	-0.97580221123863	7.14376180857948	-0.00951267117714
H	1.16734793344029	5.90606974826751	-0.00289179332312
H	3.31934465948202	4.67197076750174	-0.02746230618002
H	5.46205347944530	3.43488271072205	-0.05871003186671
H	7.60660173893209	2.19800290892768	-0.09997502611425
H	8.84612985639083	0.04337092945676	-0.14882267815012
H	7.61883161132461	-2.11970821778614	-0.17493790227676
H	5.48156188352290	-3.36779291734748	-0.17376411075446
H	3.34327909000963	-4.61687543607875	-0.16657819425336
H	1.20080114078597	-5.86213584730381	-0.15416369136722
H	-0.93383451204466	-7.11351439141405	-0.13080985149737
Nb	-2.37536225157551	0.60568745260626	2.15016030792932

Ni-G

C	-7.17620411743742	3.70332539962614	-0.00280031703391
C	-7.17868736826626	1.23975245448985	-0.01806493135031
C	-7.17122995260219	-1.23469657281391	-0.02827144381290
C	-7.15361285679002	-3.69815221366508	-0.03637102581602
C	-2.85484512624273	-6.17420225993089	-0.08247003538842
C	-2.90971635606936	3.73149382232347	-0.04796570895579
C	-2.91421056285153	1.26463274817279	-0.09455877117252

C	-2.90712399026134	-1.23313610859645	-0.12239420856656
C	-2.89253891769275	6.20569494560592	-0.02054810522043
C	-2.88683336965421	-3.70024602183510	-0.09974262775899
C	1.36920292975268	3.75272315502654	-0.06283869003342
C	1.37133372455991	1.26936502798738	-0.09676346395644
C	1.37873201187512	-1.21175345622737	-0.10659527726721
C	1.39185251952447	-3.69528230231513	-0.10484932086825
C	-11.45206513640967	1.20355366197947	0.05118237801503
C	5.63973922840132	1.28657114558969	-0.07127065056620
C	-11.44476363258078	-1.22502971793542	0.04066525538207
C	5.64728480121202	-1.20329461882764	-0.08552218348226
C	-5.77515803317475	3.73112578166456	-0.02219819487369
C	-5.76198688679411	1.24750614869136	-0.05006094914738
C	-5.75460489541234	-1.23345681166093	-0.06597535834218
C	-5.75238494127904	-3.71715175214587	-0.05697260913650
C	-1.49046582004471	-6.16998677863000	-0.09272236653223
C	-1.49648896272237	3.73597132280756	-0.06941697606907
C	-1.47737243443155	1.26869420205757	-0.10676073841963
C	-1.47027254129871	-1.22794167703507	-0.11324276834913
C	-1.52814552944724	6.20977298640391	-0.02722881188486
C	-1.47352728133031	-3.69551363124907	-0.09601849092017
C	2.77054841281487	3.73361096630016	-0.06125601570588
C	2.78822748848774	1.27028008841184	-0.07713057285394
C	2.79568419179826	-1.20418381690343	-0.09185492511707
C	2.79301629185677	-3.66764124723386	-0.10306179431593
C	-10.02994984856187	1.23856520648612	0.02726707220653
C	7.06225152800748	1.26017478007048	-0.06604057840876
C	-10.02241271870012	-1.25129442377119	0.01631301448230
C	7.06961285311206	-1.16840533411699	-0.08008749687027
C	-5.04845633907449	2.48676889576356	-0.04789257293795
C	-5.05196878939845	0.00925169407600	-0.06481786432314
C	-5.03343070714224	-2.46826491437064	-0.06961393904367
C	-5.03404880200480	4.95075001834271	-0.01720219269770
C	-5.00384334468207	-4.93227441944534	-0.06379608111284
C	-0.76474977267972	2.51008894379796	-0.08175291614129
C	-0.75089834629938	0.02286441007754	-0.14534678348714
C	-0.74953332400275	-2.46527271969685	-0.11554329209640
C	-0.76832733956374	4.98226451385150	-0.04708883747460
C	-0.73803270635461	-4.93778151714296	-0.09934547963496
C	3.50494852755005	2.52120812933144	-0.06917490161240
C	3.50566908626546	0.03524338755602	-0.09385140923340
C	3.52002267291238	-2.45073743279734	-0.09762262064869
C	-9.31174271770149	2.45330019880173	0.02176485844027
C	-9.30863847644537	-0.00412509470682	0.00931031690351
C	7.75498686923481	0.04794962566899	-0.07067318402627
C	-9.29683352235994	-2.46153007974197	0.00027855195364
C	-3.63428890175729	2.50165467309717	-0.07431484732362
C	-3.63252505892270	0.01357161604992	-0.12916834687641
C	-3.61875377579443	-2.47426493131867	-0.09191054368816
C	-3.64511621646827	4.97359673001854	-0.03066184411293
C	-3.61482091285986	-4.94665357837459	-0.08090023081328
C	0.64989757663065	2.50390475187702	-0.07810936034580
C	0.66888398262658	0.02662415533937	-0.10140997271205
C	0.66483048337680	-2.45073988554998	-0.10960354590939
C	0.62077564961486	4.96780636065772	-0.04798787301152
C	0.65091209701074	-4.91498473966700	-0.10277324875101
C	4.91407398964342	2.49687567491872	-0.06547389598575
C	-12.13739007312941	-0.01285541710641	0.05742339052652

C	4.92583927008735	0.03948044270595	-0.08137596770916
C	4.92896426226483	-2.41795001171164	-0.09379484492249
C	-7.90294981727055	2.48627110387394	-0.00034407711558
C	-7.88871272524977	0.00035979235152	-0.02092708471524
C	-7.88784154114723	-2.48572256209360	-0.02254084259348
H	-3.40331305070826	-7.11941937752200	-0.07538015104107
H	-5.54834517455779	-5.88031063035799	-0.05302851708998
H	-7.70019411301502	-4.64468709559333	-0.02862526853871
H	-9.84416297345787	-3.40767288986212	0.00463953923402
H	-11.98969877478017	-2.17164301529758	0.04586704381055
H	-13.22944192575550	-0.01623731472769	0.07567215734850
H	-12.00255673092309	2.14687115311491	0.06454493465297
H	-9.86475437809432	3.39605600891495	0.03449551333391
H	-7.72860972763790	4.64634654641581	0.01422819757678
H	-5.58436312974035	5.89536731456955	-0.00171355693029
H	-3.44671596778858	7.14748681726689	-0.00618852211366
H	-0.97947383645076	7.15484724727232	-0.01743757509307
H	1.16545698143164	5.91570728358697	-0.03439061181324
H	3.31720899780163	4.68009031946104	-0.05179263106208
H	5.46147480077551	3.44297100714009	-0.05913305227641
H	7.60723067167839	2.20674989014249	-0.05854018829138
H	8.84718491347608	0.05122987436972	-0.06691749505329
H	7.62025488507569	-2.11171982550350	-0.08353105435204
H	5.48205043187860	-3.36073968402723	-0.09824284427246
H	3.34545372445482	-4.61080074877203	-0.10292594166398
H	1.20137352297101	-5.85964009534541	-0.10316591193482
H	-0.93615883617706	-7.11181180684556	-0.09471398199910
Ni	-2.18339067871273	0.01034364836477	1.47110959891204

Sc-G-H_2

C	-7.1818391	3.6728448	0.1087423
C	-7.1791327	1.2109336	0.0587546
C	-7.1740212	-1.2625251	0.0098036
C	-7.1642976	-3.7245010	-0.0368515
C	-2.8582339	-6.1961372	-0.1448837
C	-2.9123649	3.7007043	0.0276458
C	-2.9145691	1.2333474	-0.0671703
C	-2.9090869	-1.2588590	-0.1387794
C	-2.8879796	6.1669099	0.0999580
C	-2.8938918	-3.7291553	-0.1346933
C	1.3741769	3.7236910	0.0189663
C	1.3757748	1.2385064	-0.0403805
C	1.3818183	-1.2442765	-0.0822398
C	1.3932037	-3.7306460	-0.1149667
C	-11.4534367	1.1768515	0.1458122
C	5.6439867	1.2505694	0.0443964
C	-11.4481502	-1.2509302	0.0979174
C	5.6499266	-1.2382217	-0.0007904
C	-5.7729301	3.7052224	0.0760105
C	-5.7623935	1.2200793	0.0112055
C	-5.7571040	-1.2622093	-0.0446337
C	-5.7557761	-3.7482471	-0.0715526
C	-1.4928130	-6.1929376	-0.1549922
C	-1.4870212	3.7050125	-0.0018537
C	-1.4722769	1.2368002	-0.0828005
C	-1.4670392	-1.2558326	-0.1365684

C	-1.5225496	6.1705269	0.0846831
C	-1.4687511	-3.7258497	-0.1297623
C	2.7834367	3.6985955	0.0387216
C	2.7928279	1.2365869	0.0000857
C	2.7990460	-1.2371378	-0.0440326
C	2.8017390	-3.6994570	-0.0924049
C	-10.0301382	1.2107165	0.1185245
C	7.0674992	1.2232536	0.0675978
C	-10.0244833	-1.2777946	0.0694873
C	7.0735529	-1.2046584	0.0221272
C	-5.0501771	2.4616700	0.0178985
C	-5.0500625	-0.0191694	-0.0342410
C	-5.0388414	-2.4998463	-0.0799021
C	-5.0368059	4.9186164	0.0979103
C	-5.0127448	-4.9581866	-0.0991704
C	-0.7617845	2.4809314	-0.0321405
C	-0.7539883	-0.0055775	-0.1505503
C	-0.7494858	-2.4973117	-0.1372779
C	-0.7634948	4.9454197	0.0425075
C	-0.7386937	-4.9637247	-0.1438606
C	3.5099402	2.4897322	0.0297233
C	3.5100568	0.0015542	-0.0197060
C	3.5224589	-2.4870005	-0.0587848
C	-9.3153961	2.4245064	0.1282634
C	-9.3109387	-0.0317015	0.0799280
C	7.7596786	0.0107003	0.0563802
C	-9.3041958	-2.4879147	0.0322046
C	-3.6317080	2.4736967	-0.0162483
C	-3.6280569	-0.0136343	-0.1341112
C	-3.6192929	-2.5040451	-0.1105604
C	-3.6414984	4.9378972	0.0731326
C	-3.6179622	-4.9705066	-0.1249660
C	0.6572238	2.4762241	-0.0255277
C	0.6690623	-0.0044235	-0.0702223
C	0.6697927	-2.4858396	-0.1147709
C	0.6320862	4.9333356	0.0452309
C	0.6561942	-4.9444501	-0.1370536
C	4.9232900	2.4609670	0.0533093
C	-12.1399379	-0.0388326	0.1350060
C	4.9305061	0.0045446	0.0116723
C	4.9354680	-2.4518568	-0.0355332
C	-7.9022884	2.4603905	0.0984924
C	-7.8906279	-0.0275438	0.0415479
C	-7.8911243	-2.5158999	0.0006270
H	-3.4070365	-7.1411233	-0.1512755
H	-5.5550767	-5.9069744	-0.0948554
H	-7.7107189	-4.6705990	-0.0399900
H	-9.8524095	-3.4330891	0.0239971
H	-11.9930696	-2.1972565	0.0885810
H	-13.2318879	-0.0416344	0.1553265
H	-12.0021506	2.1205914	0.1740029
H	-9.8679952	3.3667383	0.1571065
H	-7.7331262	4.6155361	0.1420963
H	-5.5835114	5.8640787	0.1363591
H	-3.4411449	7.1088008	0.1327602
H	-0.9736158	7.1152280	0.1067577
H	1.1743855	5.8816263	0.0764178
H	3.3300601	4.6442401	0.0642714

H	5.4710261	3.4062435	0.0765009
H	7.6122066	2.1694461	0.0926643
H	8.8516764	0.0131179	0.0734496
H	7.6228893	-2.1483956	0.0111511
H	5.4882696	-3.3943649	-0.0477523
H	3.3530478	-4.6427181	-0.1007636
H	1.2032085	-5.8905231	-0.1449555
H	-0.9397906	-7.1353434	-0.1713259
Sc	-2.1825575	-0.0609010	1.8401163
H	-2.1331380	1.7055658	2.8829065
H	-2.1450048	1.1881127	3.5097603

Sc-G-2H_2

C	-7.21669264534934	3.69153052582560	-0.05259314884467
C	-7.20727777665540	1.22995908537834	-0.01192967518184
C	-7.19218326210622	-1.24228287731133	0.02869206235297
C	-7.17183091414941	-3.70234707888008	0.05788146711944
C	-2.86677392919608	-6.16759655611934	-0.08984807028913
C	-2.94934911942443	3.72704421522530	-0.07924637368493
C	-2.94381193495969	1.26303148286951	-0.10945739834439
C	-2.92088533888268	-1.22605660145547	-0.14475718603872
C	-2.92905699131813	6.19470718566289	-0.03871682576294
C	-2.90176083115454	-3.69737460047214	-0.09406753005444
C	1.33276675617473	3.74793879037609	0.05306605957673
C	1.34438189819079	1.26734884027281	-0.02239489190066
C	1.35796747427398	-1.21209239060448	-0.09244687335782
C	1.37741117923251	-3.69469847289124	-0.15080734725350
C	-11.48108837386279	1.18404776429853	0.09638217809115
C	5.61254412362871	1.29386146699992	0.08778790481114
C	-11.46619040878423	-1.24339464584105	0.14139076511337
C	5.62705007293570	-1.19361433620411	0.00491649488119
C	-5.80895138110357	3.72562971975377	-0.07851875120631
C	-5.78925875343121	1.24245017530104	-0.05834234666082
C	-5.77554044033509	-1.23785167067072	-0.02634270407214
C	-5.76362329472046	-3.71927267194040	0.01157467158370
C	-1.50234738584819	-6.16419256150403	-0.13705883006237
C	-1.52954542755767	3.72755528347934	-0.03965814941452
C	-1.51074449628301	1.25732220943702	-0.12860453350030
C	-1.48770742795043	-1.23202577369281	-0.13133141620674
C	-1.56443794718982	6.19742168269275	0.00255271728490
C	-1.48215118012889	-3.69620467734532	-0.13971190958322
C	2.74115987600460	3.73035052305659	0.09239531570324
C	2.76127070147594	1.27100869225577	0.02603836306643
C	2.77621314275836	-1.20026086219087	-0.05212338973138
C	2.78527333815464	-3.66097291615647	-0.13045295264596
C	-10.05793192290907	1.22229053928259	0.05823029282273
C	7.03600026636406	1.27089126473500	0.11829097064247
C	-10.04297410985011	-1.26605955550611	0.10375531164161
C	7.05051646325489	-1.15597849683863	0.03692725752499
C	-5.08307572726383	2.48353092935068	-0.09277070210481
C	-5.07184164750270	0.00639993652084	-0.05137292152486
C	-5.05064840261929	-2.47041875529256	-0.02479087086365
C	-5.07437315667381	4.94172707898617	-0.07985105921542
C	-5.01652264201216	-4.92763757876128	-0.00701912758298
C	-0.80187529163807	2.50164193116293	-0.03874869957048
C	-0.78105931762639	0.01951164704005	-0.13900605080279

C	-0.76626567530153	-2.46688748855989	-0.15329233444968
C	-0.80624721882021	4.97098416967290	0.00874948539009
C	-0.75069012562080	-4.93413960167264	-0.15383622955880
C	3.47398608234748	2.52515459584954	0.07777606670699
C	3.48275964163903	0.03879593496335	-0.00334959725416
C	3.50337700823235	-2.44677494553177	-0.08172495623600
C	-9.34733530548164	2.43818030748521	0.01620256179923
C	-9.33444395921555	-0.01780563986621	0.06276185957372
C	7.73226778933455	0.06088203455629	0.09284441131686
C	-9.31786772497868	-2.47421488928180	0.10462562087559
C	-3.66531004262543	2.49807823661319	-0.10881285144988
C	-3.65059528783636	0.01173940762540	-0.13429968779027
C	-3.62943021115464	-2.47165551785659	-0.07159260173040
C	-3.68083632854340	4.96504136506222	-0.07130442133663
C	-3.62487756717094	-4.94142537721569	-0.06139415682763
C	0.61956347410391	2.49975984994265	0.00077818988699
C	0.64059075496962	0.02364039877173	-0.06310389280157
C	0.65157519148681	-2.45252752181664	-0.14275870114409
C	0.58563258507997	4.95643225733415	0.05647282026898
C	0.64281135578967	-4.91064837980570	-0.16784357639222
C	4.88727315877154	2.50165805591431	0.11020484427977
C	-12.16294963364943	-0.03338652369106	0.13735925018799
C	4.90381823699691	0.04637989619693	0.03146277165365
C	4.91626698059497	-2.40875978570109	-0.05215029311686
C	-7.93463770473924	2.47670504850067	-0.01924767562774
C	-7.91360773385294	-0.00975291980019	0.02137062577235
C	-7.90470862411425	-2.49712766954576	0.06500905632631
H	-3.41535445633449	-7.11250595888096	-0.07295764888444
H	-5.55778966469834	-5.87714978985377	0.01712288961391
H	-7.71292627868295	-4.65134878473988	0.08307524930390
H	-9.86217215856329	-3.42142485300693	0.13369328372767
H	-12.00764852451318	-2.19153557390549	0.17278423263209
H	-13.25465188532034	-0.03956873374509	0.16605649794710
H	-12.03376884435243	2.12619622077591	0.09283906208302
H	-9.90299931651625	3.37922216365935	0.01180827604120
H	-7.77078228022528	4.63339585831404	-0.04915856804896
H	-5.62407702832002	5.88649547563676	-0.07180365834818
H	-3.48189638219614	7.13730506034515	-0.04105092570728
H	-1.01575562064580	7.14197534771453	0.03168305480423
H	1.12701781140775	5.90549424757140	0.09267193447461
H	3.28239289098134	4.67888819380939	0.12940701512410
H	5.43161152838600	3.44844997565033	0.15075884666542
H	7.57761992197654	2.21844055743771	0.16114415773781
H	8.82426460092657	0.06661338229565	0.11642069775030
H	7.60334955703768	-2.09778924775408	0.01666630883209
H	5.47194453610260	-3.34955893366311	-0.07335453900443
H	3.33936640503345	-4.60274462107343	-0.14394290789869
H	1.19251311960830	-5.85541019938756	-0.17670871311183
H	-0.94955713854729	-7.10662308329842	-0.15645500136350
Sc	-2.21416588522720	-0.00188814114577	1.94247228994158
H	-1.64921924829725	-0.77139806305142	3.60397337872040
H	-1.25465918254079	-1.29458603466701	2.97063228107429
H	-3.17486768741001	1.27496746133750	2.98834226242714
H	-2.78035171927193	0.74329488519657	3.61466155838947

Y-G-H_2

C	-7.20489005387510	3.68249408990275	-0.02847334880609
C	-7.19784032515598	1.22164985428152	-0.02795592164808
C	-7.18468689669515	-1.25093999514918	-0.02470058164291
C	-7.16258991853894	-3.71085006353213	-0.03190102076862
C	-2.85949099969088	-6.17584279376747	-0.15211812922191
C	-2.93428082228615	3.71876391707065	-0.12407673627881
C	-2.92682737934481	1.24951410581912	-0.24505792664347
C	-2.90975757679468	-1.24081774338402	-0.20617582166975
C	-2.91739629032806	6.18520570337334	-0.05716127991824
C	-2.89501665367446	-3.70407057565428	-0.16569668661281
C	1.34858917814871	3.74488383191539	-0.02452874933561
C	1.35464275042890	1.26156990393424	-0.07759547419735
C	1.36456321603831	-1.21599832487109	-0.11964817177367
C	1.37977559417411	-3.69441730772091	-0.14329321037343
C	-11.46942220925956	1.17599928926065	0.12295959183555
C	5.62373123253588	1.28308734755692	0.02132782712721
C	-11.45692818083705	-1.25226279117026	0.12565271070566
C	5.63398757751004	-1.20498509267545	-0.02344635250242
C	-5.79618084386290	3.71528655214477	-0.07536147415167
C	-5.78178047987467	1.23291945564119	-0.09087813836142
C	-5.76696817931946	-1.24781881154880	-0.08700886522991
C	-5.75646613710889	-3.72631581331618	-0.08347972167026
C	-1.49514931886549	-6.16891044554168	-0.16759606817758
C	-1.51238912074552	3.72404541609342	-0.09886270375160
C	-1.49535702079725	1.25069236233266	-0.16072421549823
C	-1.48527145023860	-1.23172095213204	-0.18040785456221
C	-1.55225997981880	6.19039342012244	-0.04272737588405
C	-1.48014968017199	-3.69666149004147	-0.16871506037558
C	2.75677659921915	3.72491453514875	0.00638077365682
C	2.77219150958469	1.26411362505876	-0.03474639357599
C	2.78305550645450	-1.20703080458656	-0.07773708971138
C	2.78533849511374	-3.66652164219948	-0.11909310392669
C	-10.04719836989823	1.21395255230371	0.07148072447311
C	7.04713158789767	1.25846224656250	0.04985920287868
C	-10.03334565025420	-1.27522194052041	0.07414869406826
C	7.05783071318719	-1.16915938803105	0.00624591270087
C	-5.07167596580090	2.47338135544410	-0.11664426966289
C	-5.06241911156082	-0.00391002973098	-0.10041082344906
C	-5.04348084166441	-2.47840238040879	-0.11154765189382
C	-5.06086645208196	4.92936967619749	-0.07613861837362
C	-5.00846212955453	-4.93677931527883	-0.10619839532489
C	-0.78521954206668	2.49735917959348	-0.10721822004257
C	-0.77035084777643	0.01112194024774	-0.16210258767649
C	-0.76524099414761	-2.46347834244684	-0.17592590906840
C	-0.79164979082949	4.96612736005611	-0.05405436331633
C	-0.74666539538387	-4.93637843668634	-0.16972862110581
C	3.48667891384293	2.51733332287494	0.00042501983328
C	3.49186778577650	0.03127473122499	-0.04990462854964
C	3.50784613594551	-2.45355432184719	-0.08715012609434
C	-9.33610818216641	2.43014677088233	0.04366807790481
C	-9.32487171651605	-0.02692116672525	0.04817980978224
C	7.74172105079249	0.04698537396160	0.04201001564578
C	-9.30935833028858	-2.48319242114835	0.04755746990006
C	-3.65059840315107	2.48753802099484	-0.16432038184657
C	-3.63773351262949	0.00096278260053	-0.20117580170678

C	-3.62127470671107	-2.47746045464118	-0.15537136253446
C	-3.66714603184070	4.95484719054562	-0.08806596024776
C	-3.61876198145443	-4.95008196013593	-0.14119239074786
C	0.63432442903509	2.49493431348979	-0.06394291557715
C	0.64743925728250	0.01913572060290	-0.10447458787554
C	0.65415399082408	-2.45224830242612	-0.14381301017007
C	0.60278799548981	4.95265385709975	-0.02116354751047
C	0.64368091759601	-4.91136946474948	-0.16193876882546
C	4.89995836578488	2.49219522184237	0.02881418489478
C	-12.15189848077361	-0.04220260289749	0.14943957262736
C	4.91285282730866	0.03619111378790	-0.01582248315094
C	4.92066473907458	-2.41925764390384	-0.05910435207407
C	-7.92344660272389	2.46915345532194	-0.00672066367905
C	-7.90488907746142	-0.01864117684743	-0.00598387978515
C	-7.89668469851871	-2.50523584979801	-0.00322358981147
H	-3.40798104743999	-7.12081275112340	-0.14359081814034
H	-5.55158669247097	-5.88528836276301	-0.08772963070545
H	-7.70353531602097	-4.66000340237642	-0.01222256433722
H	-9.85330334191109	-3.43078287745509	0.06564705989081
H	-11.99880130391088	-2.20036826926956	0.14610325187866
H	-13.24334955408638	-0.04781528181811	0.18906489139316
H	-12.02159622439272	2.11816951532559	0.14145369212650
H	-9.89083417940626	3.37145449516223	0.06208146886811
H	-7.75616464576531	4.62533560719685	-0.00231229525102
H	-5.61097275787517	5.87337994263605	-0.04909084464010
H	-3.47326760257638	7.12566834836956	-0.04058748777742
H	-1.00446155490497	7.13540676067999	-0.01551772623091
H	1.14433263620061	5.90153940524504	0.01063971834285
H	3.30031903540889	4.67234808354102	0.03402441657811
H	5.44563041509892	3.43863950379196	0.05648994424749
H	7.59039390032279	2.20558465384187	0.07819051896328
H	8.83367267452467	0.05139835781057	0.06435944982992
H	7.60877207054780	-2.11225190802445	0.00078772137170
H	5.47359297958459	-3.36185055944099	-0.06469518267619
H	3.33603006088467	-4.61035044538657	-0.12097952027958
H	1.19538532157103	-5.85514532060449	-0.16124657893507
H	-0.93777921023376	-7.10875093285051	-0.17214064050013
Y	-2.65400367888094	0.53174280616391	2.23331759654120
H	-3.37847888252471	1.75053997473280	3.60660231834213
H	-1.79895713824991	-1.02398709316158	3.06660703943545

Y-G-2H_2

C	-7.21090967368051	3.70775670774900	-0.08462129108021
C	-7.21342894336668	1.24698409370756	-0.07664626796302
C	-7.20124494275631	-1.22264115084994	-0.02724257499962
C	-7.17542358774491	-3.68077685777983	0.04284969707109
C	-2.88246189303588	-6.15184881829501	0.06426915509420
C	-2.94759217084410	3.74062362163584	-0.12788739980845
C	-2.95452523862058	1.26734484031707	-0.18537709554238
C	-2.93718713517268	-1.20923545976428	-0.09516325340164
C	-2.93543657978828	6.21494051533746	-0.06572465056082
C	-2.91319523110494	-3.67679520587876	-0.01491134915976
C	1.32956704266639	3.76689612281060	-0.05140651513055
C	1.34067048884088	1.28516383834410	-0.12095082164556
C	1.34751002250451	-1.19702044387471	-0.11909232964773
C	1.35964633316646	-3.67501326394437	-0.05835400514301

C	-11.48969835171751	1.20755388676103	-0.01943665122521
C	5.60836052269474	1.30934809939497	-0.02062985800782
C	-11.47844481737018	-1.22339408065828	0.02556443078542
C	5.61852035331481	-1.18521796575585	-0.03696711407894
C	-5.81414857161620	3.73470039614463	-0.09865743704341
C	-5.79660599797210	1.25535505184341	-0.09070378716326
C	-5.78413734148903	-1.21906383204069	-0.03360047833349
C	-5.77740672515783	-3.69563677846253	0.02756555648538
C	-1.51935945061566	-6.14979957766503	0.04441491474142
C	-1.54093253602674	3.75033634473965	-0.10661146521512
C	-1.52564529869562	1.27569659014641	-0.20626725963547
C	-1.51224146053767	-1.20770695126614	-0.11485380461223
C	-1.57206064061390	6.22265613412120	-0.05114762211474
C	-1.50645023815704	-3.67555913983961	-0.02128256478507
C	2.72569189817651	3.74811814895657	-0.02820305576132
C	2.75241536851435	1.28684538010787	-0.07084088908277
C	2.76309701626241	-1.18513919794204	-0.08034881837988
C	2.75629607899307	-3.64579264240737	-0.05316557875680
C	-10.06953178274109	1.24516707152885	-0.03408623188981
C	7.02562900874057	1.28444694471834	0.00226902708880
C	-10.05735954028345	-1.24707800928577	0.01211841862839
C	7.03827018789569	-1.14842859803416	-0.01444313366685
C	-5.08460322098234	2.49131298479664	-0.11462054202440
C	-5.07723187173063	0.02088253509352	-0.09319744892044
C	-5.05955761725753	-2.44704946513553	-0.02083271758744
C	-5.07249540263311	4.95460886565780	-0.09051677270299
C	-5.02702020449301	-4.90943485447796	0.05592353233947
C	-0.80444550762155	2.51968252530279	-0.15411877727142
C	-0.79502557393234	0.03156618446782	-0.21444694234900
C	-0.78459307470602	-2.44212464557764	-0.08199035714888
C	-0.81026354399527	4.99657822336725	-0.06648236105046
C	-0.76815903472374	-4.91817467417040	-0.00171678431346
C	3.47133371871399	2.53838240293719	-0.03869484897703
C	3.47301554688236	0.05315940032840	-0.07882205580931
C	3.49189690149545	-2.43044479812233	-0.06333318769218
C	-9.34705159681714	2.45957416235182	-0.05759293525685
C	-9.34455944527582	0.00253494527451	-0.02064774586501
C	7.71664714732148	0.06993869289628	0.00374325600195
C	-9.32358140431821	-2.45365451255937	0.03192396323785
C	-3.67173960570909	2.50159022750882	-0.13307993716680
C	-3.66109424755448	0.02518181138716	-0.12317264094674
C	-3.64409940871828	-2.44495775862019	-0.02798661279680
C	-3.68591805926273	4.98172657432855	-0.09401812816741
C	-3.64089896414069	-4.92378917925908	0.03817692475773
C	0.61038703906653	2.51781781085300	-0.09915584216780
C	0.63006816537601	0.04063913754257	-0.14579445439827
C	0.62898256240996	-2.43189872469031	-0.10050774939188
C	0.57574126699249	4.98043085213745	-0.04223185415129
C	0.61749647999983	-4.89471698453593	-0.01957502535413
C	4.87233376865262	2.51884143423792	-0.01655530649986
C	-12.17000993899323	-0.01141466738016	0.00918412799445
C	4.89451030700422	0.05907742219989	-0.04432565843483
C	4.89438682014084	-2.39935558383207	-0.04672082313548
C	-7.94282470954162	2.49092043691537	-0.07390784903530
C	-7.92266200973279	0.00891279317539	-0.03357157601331
C	-7.91773910330181	-2.47220582397005	0.01738798805106
H	-3.43318818736711	-7.09491756996637	0.10032275774128
H	-5.57106353584766	-5.85703419652285	0.09376989524666

H	-7.71734273548520	-4.62943889195403	0.07752657640068
H	-9.86607169877753	-3.40201892606532	0.06100547782072
H	-12.02198985565654	-2.17021368161296	0.04958840029925
H	-13.26239343935497	-0.01625772408221	0.02000130906607
H	-12.04283697811930	2.14906433147165	-0.03015153879601
H	-9.89905669013011	3.40283248424148	-0.06027838993305
H	-7.76182402626658	4.65177484442506	-0.07692298931296
H	-5.62411957011558	5.89842865951342	-0.07246889818230
H	-3.49278396958536	7.15464952121369	-0.05146068267082
H	-1.02459845523349	7.16781254008521	-0.02595057349848
H	1.11947733761812	5.92828061483134	-0.01177010471724
H	3.26809430859210	4.69654143425764	0.00046237048026
H	5.41609744647581	3.46648497646735	0.00686115674579
H	7.57106997414845	2.23024714613297	0.01992365492856
H	8.80907706885878	0.07548722290578	0.02132175978982
H	7.59098687886960	-2.09015119456779	-0.01006001484231
H	5.44596260952049	-3.34276477838003	-0.03652247508785
H	3.30692199198196	-4.58976035220798	-0.03046727918238
H	1.16908492187688	-5.83830500535010	0.00182552700030
H	-0.96412535769858	-7.09066494560202	0.06309485668007
Y	-1.23723223449376	0.69071733206468	2.29420560591492
H	0.25720144054745	1.25690015778815	3.43657051294449
H	-2.79191343330569	0.16920251287211	3.37706178820542
H	0.36836452071919	-1.10280672977353	2.93103077452070
H	-0.03357068304671	-1.56381334723462	2.47922976862489

Zr-G-H_2

C	-7.18622956924869	3.69340683671787	0.02178253705158
C	-7.18981328857450	1.23172555230753	-0.00918582693873
C	-7.18376332385467	-1.23961175435750	-0.03286679651366
C	-7.16136472639966	-3.69864832749781	-0.04305174915036
C	-2.86914452127620	-6.17429151210747	-0.12061715609612
C	-2.92751663144429	3.72672754265578	-0.07529259998220
C	-2.92959638907659	1.25335766271034	-0.09795510102654
C	-2.92229576080583	-1.23099226797714	-0.16025336785321
C	-2.91040310403803	6.20053776300074	-0.01638476194828
C	-2.89836450804686	-3.69878980413601	-0.13385460352221
C	1.34911755416745	3.74582862430763	-0.05900809595348
C	1.35797805868354	1.26457895526496	-0.11668450943988
C	1.36394248122580	-1.22035529215346	-0.12596796359865
C	1.37548729525860	-3.69921209708654	-0.13059084130607
C	-11.46478553209753	1.19870236386650	0.09629504047346
C	5.62495183728755	1.28393877343316	-0.01379016524855
C	-11.45904962594357	-1.23274303405114	0.07408622430633
C	5.63350105732609	-1.21113193875682	-0.04031093880696
C	-5.78951966874221	3.72290199975447	-0.00959538393435
C	-5.77566932525118	1.24043065847792	-0.03312173126420
C	-5.76694176066349	-1.23833007432709	-0.05586115586821
C	-5.76259452260989	-3.71552679553041	-0.07656041182238
C	-1.50587102878217	-6.17250346648458	-0.12952824601344
C	-1.51884958884356	3.73070458603943	-0.08206969343402
C	-1.49953376290323	1.25785252421300	-0.22849733399129
C	-1.49041732384055	-1.22962878095254	-0.16318296574439
C	-1.54714103287298	6.20451144864344	-0.01949228694855
C	-1.48919837727339	-3.69765941253391	-0.13129213055201
C	2.74614095287743	3.72529890829842	-0.03156760768682

C	2.76851693015365	1.26408494469651	-0.08477847272349
C	2.77935917142647	-1.20908417837904	-0.10596555260525
C	2.77371584699749	-3.66989055926313	-0.10939202130392
C	-10.04611430919362	1.23278929841344	0.06449853409527
C	7.04157348805125	1.25864573433420	0.01272432595400
C	-10.03796684451102	-1.26044314926188	0.04143703320180
C	7.05394788698975	-1.17386109110604	-0.01362971872128
C	-5.05999214054043	2.47871998198555	-0.04077466146005
C	-5.06042562742949	0.00380993309705	-0.07477753418878
C	-5.04383490170876	-2.46753874205659	-0.09440784369801
C	-5.04978236239036	4.94363350725479	-0.00726154757051
C	-5.01367007416701	-4.93006309514768	-0.09187492931782
C	-0.78332377126599	2.50296582506028	-0.13600126075365
C	-0.77263441133966	0.01646917202123	-0.26435255394136
C	-0.76634677520705	-2.46568497881552	-0.14375168806421
C	-0.78813802064724	4.97652951273456	-0.04842298696664
C	-0.75284839680783	-4.94102010447902	-0.13372403649090
C	3.48925109177164	2.51497041916470	-0.04083930416355
C	3.48886811803883	0.02903252647107	-0.06725570625108
C	3.50720977331070	-2.45539988213923	-0.09082038938767
C	-9.32104293948577	2.44678399632180	0.05941334874346
C	-9.32274973326894	-0.01121738963069	0.03629531984484
C	7.73263460298779	0.04344821165828	0.01209414317165
C	-9.30789525866497	-2.46874933442501	0.01490085260398
C	-3.65086982816125	2.49207394003647	-0.07159241712392
C	-3.64497625467578	0.00523817825488	-0.12528480177642
C	-3.62842960492131	-2.46865459524041	-0.11732325969254
C	-3.66279868928656	4.96821361227558	-0.03374794145606
C	-3.62687614640759	-4.94539865403072	-0.11640038433722
C	0.62863052752379	2.49826592710194	-0.10896108694733
C	0.64893763033828	0.01982640836133	-0.15179332848859
C	0.64792445716753	-2.45605696413647	-0.14644170831524
C	0.59817789560713	4.96049259460226	-0.03979217223121
C	0.63357795823677	-4.91912731352770	-0.13241252232443
C	4.89023329716676	2.49393757173402	-0.01095254351059
C	-12.14758482537917	-0.01998192020588	0.10072649106246
C	4.90932157959198	0.03400516108603	-0.04190604734223
C	4.91110850252562	-2.42445096621855	-0.06432527648870
C	-7.91810028858076	2.47655972670330	0.02640120905430
C	-7.90148377852138	-0.00681278694977	0.00805956303333
C	-7.90218018231082	-2.48960215821164	-0.01985069267810
H	-3.42015372978239	-7.11783224697116	-0.11373821812909
H	-5.55844194493442	-5.87786474243384	-0.07801808312555
H	-7.70450320324502	-4.64717669134514	-0.03159995767702
H	-9.85266268002026	-3.41614200014274	0.02193209674590
H	-12.00492416887066	-2.17853707418284	0.07816361123829
H	-13.23974466291823	-0.02296628116350	0.12560816213835
H	-12.01587406840778	2.14119808843630	0.11749109181952
H	-9.87187647875633	3.39051526387909	0.08234102826725
H	-7.73806165845186	4.63672496825812	0.04531296716328
H	-5.60117639764145	5.88709109952845	0.02256257831123
H	-3.46525366719333	7.14149961218086	0.00699747295715
H	-0.99715325798874	7.14835186059213	0.00032964194558
H	1.14333164636592	5.90755154227297	-0.01071672509624
H	3.29022230152561	4.67264787849476	0.00304736281941
H	5.43502448616092	3.44094188098751	0.01663343289077
H	7.58723100989164	2.20424562775342	0.03400009152544
H	8.82494032081053	0.04848950356274	0.03259305673734

H	7.60637737289796	-2.11577753973720	-0.01300248274117
H	5.46247422170985	-3.36797879489897	-0.06095113551284
H	3.32443974802400	-4.61399329861108	-0.09902331996686
H	1.18411101963411	-5.86346466860820	-0.12760922261595
H	-0.95182361098386	-7.11423256297424	-0.13111053787715
Zr	-1.61116011753307	0.31523455438437	2.00585395316918
H	-0.49436174871358	0.99647301745560	3.34768605323216
H	-2.89086118882894	-0.82414095860042	2.71899927414978

Zr-G-2H_2

C	-7.16614385821355	3.71659542488314	-0.02542217246286
C	-7.16511521958751	1.25193428663022	-0.04467689579269
C	-7.15480142000189	-1.22022906469444	-0.02828842521972
C	-7.12877487814570	-3.68310536981900	0.03365866407941
C	-2.83284478513925	-6.14681283440868	0.19693308019529
C	-2.90681134023366	3.75130889815956	0.06324260481950
C	-2.91372796995352	1.27720641521591	0.11504789451459
C	-2.90141786211287	-1.19734598351325	0.12569368392128
C	-2.89215967515931	6.22553804231038	0.06285607909701
C	-2.86744605759865	-3.67164938921257	0.15915733359582
C	1.37140031727056	3.78210595770694	-0.06742966111332
C	1.37926394945582	1.29618771131702	-0.08665574874593
C	1.39023038934409	-1.18631828755864	-0.05507930960422
C	1.40696092186203	-3.67104396370577	0.00751882237447
C	-11.43555887353642	1.20956639872187	-0.15993527708941
C	5.64267219614603	1.32005431139518	-0.22287317755383
C	-11.42465677764665	-1.22040402845605	-0.14417452357958
C	5.65411799861754	-1.17147042553051	-0.20052936813426
C	-5.76920185930105	3.74838657210016	0.01748249469182
C	-5.75272469574479	1.26322226707044	-0.00059231112603
C	-5.74056079975304	-1.21648746988389	0.01857078504099
C	-5.73197110957844	-3.69860873526021	0.08040861694359
C	-1.46942912618663	-6.14266590476438	0.18040819717767
C	-1.49390890656250	3.75771176135376	0.04734279976225
C	-1.46224029023453	1.28086426787818	0.02560736349706
C	-1.44848018608292	-1.19381737145965	0.13220809299453
C	-1.52858834213616	6.23156487089625	0.04714782425764
C	-1.45533472452246	-3.66822135743362	0.13175863827978
C	2.76787227666751	3.76289112879029	-0.12192462469779
C	2.79196621218020	1.29829665278896	-0.14151974083013
C	2.80270745524368	-1.17523247347383	-0.11334769187291
C	2.80230450429413	-3.64039353455186	-0.05272031567203
C	-10.01776561562667	1.24677595085640	-0.11680624380955
C	7.06089372198715	1.29624545651005	-0.26911830868924
C	-10.00542363546917	-1.24438589744071	-0.10036326986397
C	7.07171375819204	-1.13417391402351	-0.24792922688581
C	-5.03802983533909	2.50443288802052	0.02940579238869
C	-5.04161262020036	0.02608482960791	0.00214339505159
C	-5.01297914914125	-2.44766425211728	0.06561490965769
C	-5.03237437776678	4.96997844863365	0.04427253039991
C	-4.98208790110303	-4.91119939557479	0.13851905036684
C	-0.75479911180918	2.53090434498117	0.01986639623056
C	-0.74176549154966	0.04674460424734	-0.04035226520517
C	-0.72712420754383	-2.43682356986406	0.10218306171159
C	-0.76731879208424	5.00494006853924	0.02351851751953
C	-0.71821834546397	-4.91082360906670	0.13435807953208

C	3.50928184994457	2.55073563017349	-0.15525215082080
C	3.50993759226806	0.06490696096215	-0.14248677504816
C	3.53292835803328	-2.42142241678209	-0.10739383816456
C	-9.29716168804912	2.46491849881045	-0.09495501699204
C	-9.29365101558423	0.00477634418629	-0.09119411155709
C	7.75312975527949	0.08412081273273	-0.28176757116883
C	-9.27410412486618	-2.45482358828209	-0.06118112449202
C	-3.63210520183291	2.51747021070591	0.08392060014211
C	-3.62459617453478	0.03125371528941	0.04121089730921
C	-3.60576907584061	-2.44384676202023	0.12501586134124
C	-3.64350116898334	4.99270505091356	0.06144284878514
C	-3.59364956826160	-4.92002581212222	0.16775250427928
C	0.65166775004625	2.53100562604749	-0.05556486302418
C	0.67976548578877	0.05156691293229	-0.06978545471151
C	0.67455692515807	-2.42761008853997	0.00547184954789
C	0.62073386651579	4.99500545380553	-0.02525617576745
C	0.66901732762977	-4.89063325062423	0.07772916883074
C	4.91083223901107	2.53129995255217	-0.20185060942447
C	-12.11738348550506	-0.00925604771578	-0.17418180067183
C	4.93075173521117	0.07102516178251	-0.19141466832059
C	4.93348736060615	-2.38931169546667	-0.15832621973509
C	-7.89521278792356	2.49707060057029	-0.05330441291740
C	-7.87267862718482	0.01199269500212	-0.04906421240853
C	-7.87160332586963	-2.47230948243369	-0.01774152378081
H	-3.38161918887249	-7.09138818969673	0.22163069872853
H	-5.52357605834775	-5.86076468138788	0.14884465887443
H	-7.67257258220287	-4.63141944093384	0.04034184234313
H	-9.81941414401795	-3.40202449323798	-0.06142288125580
H	-11.96873510032573	-2.16736902868731	-0.15268780489042
H	-13.20912439309973	-0.01447344961752	-0.20769897728182
H	-11.98853534979779	2.15115453989843	-0.18069601225477
H	-9.85165006423723	3.40670151536494	-0.10886720366867
H	-7.72047410517765	4.65873159191276	-0.03642171471024
H	-5.58370004027482	5.91381697760403	0.04230658286711
H	-3.44783880615491	7.16631948173323	0.07218084481215
H	-0.98074118214562	7.17692324321385	0.04269118799486
H	1.16224024867748	5.94445884820443	-0.03732948272762
H	3.31300828663595	4.71029204896748	-0.13332966292519
H	5.45647684044759	3.47813794514291	-0.21862999081661
H	7.60495310584830	2.24288005973599	-0.29320090563030
H	8.84484690854161	0.08863825574143	-0.31720884198047
H	7.62496712683551	-2.07578021630590	-0.25574570472466
H	5.48819111931118	-3.33099170427762	-0.16141483044163
H	3.35646120850203	-4.58261243805689	-0.05146968644931
H	1.21902760254183	-5.83525503251383	0.08180431363502
H	-0.91416346748400	-7.08364531099242	0.19321694906435
Zr	-1.99587918461251	0.17987292209253	2.14169201921273
H	-0.93981177162927	0.53344422815982	3.67901804413770
H	-3.75619100506636	0.26306811862387	2.70069935859988
H	-2.63396188915998	-1.73165760896818	2.70071892417615
H	-1.84106397652177	-1.86083739100010	2.67356892392834

Zr-G-3H_2

C	-7.20336195020860	3.71802637195537	-0.06284987501869
C	-7.19783148599651	1.25707027147440	-0.06257045749646
C	-7.18525838393442	-1.21590749471010	-0.03035495918820

C	-7.16581786221104	-3.67591423452700	0.03405422734353
C	-2.85437640377942	-6.13479609158548	0.11505475595547
C	-2.93334102974260	3.75214396980332	-0.04291970935768
C	-2.93184474782469	1.28325432682095	-0.03377306385927
C	-2.91896813794821	-1.19816181041027	-0.00225954874357
C	-2.91696233391323	6.22151941458742	-0.04591297566334
C	-2.89567371192933	-3.66650821075642	0.05203903387179
C	1.35098985811255	3.78560976587345	-0.10354379572802
C	1.36064681281198	1.30280507805839	-0.10020132862866
C	1.37321605266090	-1.17656571033738	-0.06982592434033
C	1.38882454711127	-3.65873872276617	-0.01091258695000
C	-11.47289057633043	1.21282289375185	-0.06882947328693
C	5.62854543118843	1.32863570338687	-0.13227588369468
C	-11.46053274866091	-1.21498837135478	-0.03679925676118
C	5.64121604547092	-1.16000643173601	-0.10157779386571
C	-5.79641655839187	3.74925112623859	-0.05257553906812
C	-5.77976259782512	1.26696274279634	-0.05132850396393
C	-5.76715757163849	-1.21119928560797	-0.01944436219051
C	-5.75857073108229	-3.69270817618774	0.04447069401121
C	-1.48955314654310	-6.12907888853274	0.10330158362778
C	-1.51196684390324	3.75940410409826	-0.06632321724359
C	-1.49468155840987	1.29089529091932	-0.07064329835373
C	-1.48188820305763	-1.19265459923562	-0.04096386664625
C	-1.55214539804169	6.22917203598727	-0.05647176757051
C	-1.47418261929706	-3.66028766119224	0.02754848244788
C	2.75925307847788	3.76849614725358	-0.12078123002786
C	2.77828620470525	1.30684441093502	-0.11735230189155
C	2.79081436691131	-1.16666048707609	-0.08710007404993
C	2.79686941076297	-3.62774600436029	-0.02887711389758
C	-10.04885918645004	1.25034222336739	-0.06313495985093
C	7.05243361591940	1.30527270996344	-0.14556271803641
C	-10.03621917956121	-1.23801493713224	-0.03038536277416
C	7.06475938990295	-1.12264835746141	-0.11556598244458
C	-5.06970682049960	2.50608381844718	-0.04929306147712
C	-5.06540064607220	0.03117433163908	-0.05775094004267
C	-5.04447937301185	-2.44259327182866	0.01446722999142
C	-5.05964094417183	4.96429626790719	-0.04553004306856
C	-5.00962168946610	-4.89961381061246	0.08339168179163
C	-0.78162332275319	2.53862284927559	-0.07276772594287
C	-0.76659281926074	0.05198924368453	-0.12949361327437
C	-0.75621811984006	-2.43288955637598	-0.01146078933002
C	-0.79019581628272	5.00487474790104	-0.06874044337463
C	-0.73992311362107	-4.89802532710098	0.05733377021713
C	3.49132684657401	2.56176698724211	-0.12388614466717
C	3.49821152322476	0.07376838571755	-0.09665691383208
C	3.51657000112263	-2.41409496860332	-0.06247516435943
C	-9.33675482609419	2.46596289302809	-0.06980150528941
C	-9.32647540516787	0.00980167590416	-0.04689712187709
C	7.74793380049191	0.09472535988379	-0.13746417207562
C	-9.31180957629569	-2.44611568276712	-0.00518797133212
C	-3.65117420958559	2.52205064599295	-0.04656084009665
C	-3.64949898638432	0.03826592473602	-0.05905571843786
C	-3.62581463249014	-2.44420719431214	0.01683288376871
C	-3.66633346354837	4.98922688500340	-0.04384180621927
C	-3.61608962617676	-4.91046105836878	0.08467602264992
C	0.63715109240977	2.53704922160488	-0.09733385415145
C	0.65953788327320	0.05948935926459	-0.09086991282766
C	0.66240974495151	-2.41769938645179	-0.03663424337611

C	0.60214396868541	4.99385377916592	-0.08786345973680
C	0.65229728188612	-4.87363003198302	0.03677152823668
C	4.90460183594530	2.53744996155823	-0.13657316301149
C	-12.15603252243913	-0.00461077903174	-0.05650124711532
C	4.91910050299012	0.08074504391816	-0.11159627536096
C	4.92951914443180	-2.37580505755944	-0.07588672715661
C	-7.92364539780210	2.50377533563555	-0.06417556632627
C	-7.90534529908625	0.01704629889014	-0.04014246124346
C	-7.89836043809721	-2.46950976328307	0.00098861955515
H	-3.40129911535594	-7.08030854046347	0.14572086805427
H	-5.54976271599328	-5.84965932355793	0.10866261814782
H	-7.70845448306566	-4.62411766546197	0.05574455955053
H	-9.85709005049686	-3.39306122954837	0.01288255218213
H	-12.00304323068183	-2.16291061835408	-0.02513133773876
H	-13.24822077309487	-0.01027668719827	-0.06107369450560
H	-12.02480637606165	2.15527526145203	-0.08212093072789
H	-9.89158382607844	3.40749126389794	-0.07670260263197
H	-7.75559318527520	4.66091049828223	-0.06642077413617
H	-5.60930589315249	5.90919520857259	-0.04604333907937
H	-3.47337191184475	7.16196702108028	-0.04141601894301
H	-1.00581056599781	7.17546426628973	-0.05978254340813
H	1.14276578573296	5.94390541081268	-0.09068428509416
H	3.30167782880002	4.71690835864319	-0.12695622252541
H	5.45021677206563	3.48424965898300	-0.14772380095783
H	7.59504696202177	2.25307096916176	-0.16170300356731
H	8.84006363877517	0.10012928621757	-0.14788839109845
H	7.61689828995763	-2.06503833298325	-0.10833162478911
H	5.48471623889395	-3.31700586828473	-0.06352395622570
H	3.34896420784653	-4.57040687590914	-0.01090753902719
H	1.20239621611116	-5.81796029271590	0.05868552944001
H	-0.93375088969956	-7.06959497231333	0.12517026527142
Zr	-1.80221854887477	0.07167510490154	2.17267997941082
H	-0.66316146360436	0.09394889392524	3.66520989326744
H	-3.53773464262000	0.07256569793086	2.81603165669020
H	-2.39518297919775	-1.89631788223437	2.75569645786339
H	-1.61918804607710	-1.98858375177530	2.85199045956917
H	-2.37664378313217	2.06339522665859	2.71227047184160
H	-1.60338188509378	2.13688367356787	2.83719205529437

Zr-G-4H_2

C	-7.19571368633125	3.72177432420188	-0.07974153350501
C	-7.19195057778809	1.26063309023530	-0.06586406325249
C	-7.18040472798279	-1.21121657424050	-0.02458834158324
C	-7.16276732660815	-3.67153079187390	0.03862236678185
C	-2.85186191152823	-6.13112772941498	0.18001363533368
C	-2.92476783013722	3.75421569044068	0.01310410119923
C	-2.92371475357155	1.28428246266414	0.01403600682126
C	-2.91188268568944	-1.19516434107214	0.06510022895379
C	-2.90729175032207	6.22252953987241	0.01084619096685
C	-2.89247666800655	-3.66401531812739	0.11863023263665
C	1.35999181144838	3.78528104756033	-0.05863214034248
C	1.36721312960989	1.30170747383375	-0.07749553540326
C	1.37819429298297	-1.17572318757881	-0.05894250082018
C	1.39311212354431	-3.65789382471366	0.01918321295222
C	-11.46579655985978	1.21803770377959	-0.14501231547056
C	5.63529679057842	1.32461263007938	-0.17950814541990

C	-11.45499422973284	-1.20963972587988	-0.10498206607448
C	5.64621289225767	-1.16336218454497	-0.15632417556708
C	-5.78806371393267	3.75298838687052	-0.04549860526029
C	-5.77346911991460	1.26952506280359	-0.05957463441376
C	-5.76229749857244	-1.20755719200414	-0.01265167998816
C	-5.75466848939457	-3.68984427772626	0.07217370743547
C	-1.48659832212187	-6.12474412539174	0.16895482597324
C	-1.50176396763741	3.76108093188242	-0.00006125558518
C	-1.48249214585104	1.29147449944952	0.02322341444760
C	-1.47099231571185	-1.18815632669446	0.03312849758435
C	-1.54198168939993	6.22893790801679	0.00914781908154
C	-1.46969696641594	-3.65753134057467	0.09323758757607
C	2.76781955299924	3.76591150453554	-0.10291219074950
C	2.78518459507953	1.30484541011575	-0.10108711547503
C	2.79658012464301	-1.16720197014919	-0.07813151758447
C	2.80038952738892	-3.62786333423910	-0.02806918372997
C	-10.04214272405781	1.25496875513713	-0.12000292416763
C	7.05898478889363	1.30060412284639	-0.21653784941590
C	-10.03103035221690	-1.23280652722047	-0.07892050925884
C	7.06960480897509	-1.12729434704290	-0.19388309140134
C	-5.06188329702535	2.50953037295826	-0.03476621308009
C	-5.06194858254479	0.03431101989764	-0.03106786610267
C	-5.03979527576183	-2.44029603620392	0.04485421033026
C	-5.05115989494867	4.96675101107231	-0.02170489524593
C	-5.00762426162733	-4.89646052507638	0.12798433759488
C	-0.77191038739013	2.53950259070514	0.00388506640946
C	-0.75391517744929	0.05324011891328	-0.04547015683594
C	-0.75013957518993	-2.42974463930458	0.05430494741689
C	-0.78057380789852	5.00480110272608	-0.00198474710480
C	-0.73711097814233	-4.89416690901005	0.11848145041526
C	3.49875537470272	2.55897026680369	-0.12452322321110
C	3.50392679440480	0.07165309482571	-0.12315559847713
C	3.52116582602004	-2.41525998050254	-0.07678384428491
C	-9.32940615660668	2.47075325625766	-0.11969367731141
C	-9.32135947443088	0.01445974334463	-0.08990481889846
C	7.75354660148775	0.08950835769756	-0.22411004079092
C	-9.30732561523587	-2.44135742941817	-0.03907724605686
C	-3.64428787986663	2.52600870546496	-0.00806796110763
C	-3.64140945072048	0.04145796078407	-0.02228332765028
C	-3.62252766460617	-2.44330034546853	0.07694085018797
C	-3.65714250063174	4.99116728736557	0.00211122592704
C	-3.61353732357509	-4.90762612347568	0.14433641899581
C	0.64503046296793	2.53556030128992	-0.04518565416870
C	0.66678468781308	0.05988135962679	-0.06098982018532
C	0.66688417673966	-2.41454206571668	0.00307180709128
C	0.61339734487317	4.99307628255021	-0.02835113138428
C	0.65662945373826	-4.87053951783090	0.08295173684505
C	4.91181536159796	2.53389361263778	-0.16514968143460
C	-12.14977738574442	0.00088852827868	-0.13819573564157
C	4.92562369075833	0.07760388532532	-0.15234632696938
C	4.93358271050888	-2.37863968390864	-0.11852110722885
C	-7.91665871607618	2.50836611227425	-0.09094483702026
C	-7.89950445373683	0.02107006405706	-0.07242867661232
C	-7.89397323660257	-2.46548525331769	-0.00932058380496
H	-3.39842904379672	-7.07689539655386	0.21023619046144
H	-5.54833242006270	-5.84622783459116	0.14940146227801
H	-7.70604031341352	-4.61948856468432	0.05323006994811
H	-9.85323241179992	-3.38806001508197	-0.02853524190367

H	-11.99796021338898	-2.15737985473368	-0.09756592532961
H	-13.24182411562195	-0.00424499551971	-0.15783235895562
H	-12.01720861647351	2.16063315034970	-0.16867765066770
H	-9.88384009273367	3.41233147052558	-0.13959363681622
H	-7.74700696983313	4.66507864988892	-0.09469323653319
H	-5.60003719304258	5.91208548379165	-0.02941372875281
H	-3.46297984563327	7.16343875975561	0.01176798421331
H	-0.99516235071076	7.17503867388803	0.00990457430846
H	1.15424605266036	5.94298746306186	-0.03329018265936
H	3.31105841301597	4.71387111071185	-0.11848126697445
H	5.45770017725372	3.48048152177697	-0.18388597248922
H	7.60191841584277	2.24814237340035	-0.23809430522796
H	8.84539387447823	0.09396620339391	-0.25279409758329
H	7.62086450337812	-2.07026965797366	-0.19769093948385
H	5.48786038954695	-3.32051794280846	-0.11875590814240
H	3.35156847153353	-4.57133304184977	-0.02302605878603
H	1.20561176441631	-5.81569844560210	0.09914690437626
H	-0.93070061609051	-7.06526473863920	0.19131399017842
Zr	-2.18186609198152	0.07221277314624	2.16697633843576
H	-0.47614135091284	0.12464208001700	2.92375938255633
H	-3.87064229281772	0.00172776972597	2.95961868186145
H	-2.36225093796364	-1.98387810552517	2.83655876376005
H	-1.57983831444019	-1.89101810519228	2.82875225376245
H	-2.79197243389092	2.04523878702518	2.79404308195583
H	-2.01060778155187	2.14711880574398	2.78927241466614
H	-2.56505043095994	0.06700006628324	4.21608489759385
H	-1.74933404042195	0.11550760481043	4.20488818606876

Pd-G-H_2

C	-7.14848574366828	3.70520999215768	-0.00948571708343
C	-7.15381227397867	1.24413369222240	0.01644979070333
C	-7.14686945378976	-1.22896619837119	-0.00132107627425
C	-7.12807373661899	-3.68970011533702	-0.05555431525714
C	-2.83583215624446	-6.16495018747598	-0.09100459247256
C	-2.88709589696591	3.73058165485933	-0.00944451698597
C	-2.87754971980877	1.26683690779938	0.03127473869782
C	-2.87074908952457	-1.22650309754866	0.02399027348015
C	-2.87155434229250	6.20606015649374	-0.04478083816461
C	-2.86633834593725	-3.69015816717419	-0.03659291755511
C	1.38190493162501	3.75168136230350	-0.08043590965176
C	1.38121164389132	1.27045782112160	-0.08280228201294
C	1.38831820654478	-1.20436177768536	-0.08660033337925
C	1.40337955296964	-3.68545839727476	-0.09556367970915
C	-11.42705693161594	1.20996931535865	-0.01798365058886
C	5.65118680028429	1.28958741176235	-0.14695959400076
C	-11.42019791048526	-1.21818591246809	-0.03502706624972
C	5.65840278365842	-1.19901670524220	-0.15070359275304
C	-5.74709760434416	3.73043974814556	-0.01276238790743
C	-5.73781325792709	1.25336611684284	0.02239298725165
C	-5.73127039012686	-1.23034229355660	0.00539413110201
C	-5.72646111250818	-3.70706167549308	-0.05474280781350
C	-1.47195010817633	-6.15957495879410	-0.09372485637277
C	-1.47712514399656	3.73301911203953	-0.03910657900922
C	-1.46076292123308	1.26302607604268	-0.01709461302505
C	-1.45327902987495	-1.21334476819043	-0.01868606955774
C	-1.50765932151818	6.20902793238571	-0.05401071140450

C	-1.45609171346683	-3.68332086971810	-0.06118396881133
C	2.78230383690470	3.73328036687838	-0.10464282990833
C	2.79859680412911	1.27167190834015	-0.09775601717559
C	2.80559466660988	-1.19758617689360	-0.10162147459776
C	2.80366816667013	-3.65919767380238	-0.11830648206276
C	-10.00485661579750	1.24446131113668	-0.01066118093654
C	7.07358425394756	1.26349967593779	-0.16846395812357
C	-9.99783636560134	-1.24472743083075	-0.02810747406974
C	7.08056504584226	-1.16463989179643	-0.17207513059423
C	-5.02082305236478	2.48693689061893	-0.00046842970921
C	-5.02682255784176	0.01331355595986	0.05467359401653
C	-5.00789566617589	-2.45964846465818	-0.02914425087103
C	-5.00970988838112	4.95108463022226	-0.02514615425068
C	-4.98163560370392	-4.92307223329221	-0.07366481037629
C	-0.75207454288641	2.50083963269724	-0.03152327965492
C	-0.74589114363119	0.02725618892001	-0.03805073345927
C	-0.73764053782988	-2.44743154525616	-0.04218457439230
C	-0.75053286565358	4.98040776571437	-0.05247807322921
C	-0.72215971520074	-4.92655067392214	-0.08040527786041
C	3.51591078901669	2.52142444279513	-0.11387993324059
C	3.51665396280786	0.03916665780522	-0.11651782924125
C	3.53028357724352	-2.44315466116596	-0.12193413038700
C	-9.28615352747526	2.45902526769743	-0.00827114293869
C	-9.28361184737278	0.00184418650231	-0.01102102428377
C	7.76632058472239	0.05145579354515	-0.18026565564897
C	-9.27238260416538	-2.45524550873028	-0.04179802747635
C	-3.60440094175540	2.49656589540914	0.00260141044308
C	-3.59341618897300	0.01884308744123	0.09067683521818
C	-3.59128158225072	-2.46080557109843	-0.01660896781589
C	-3.62191830761678	4.97371857877660	-0.02853813508737
C	-3.59372076997399	-4.93736472364069	-0.06957144478074
C	0.66310293963113	2.50223479479583	-0.06348545609676
C	0.66982233180994	0.03098026139853	-0.05314514502162
C	0.67739806022219	-2.44044654099251	-0.07181222696304
C	0.63741875081427	4.96740379560539	-0.07312036988613
C	0.66572427704434	-4.90524845752133	-0.09680425351812
C	4.92480418299679	2.49925043856925	-0.13619381544799
C	-12.11290435789060	-0.00602152254964	-0.02873729149236
C	4.93709644731859	0.04319908023216	-0.13659177083284
C	4.93901797702981	-2.41290308015330	-0.14391961468162
C	-7.87747571593981	2.49025938044029	-0.00188271706484
C	-7.86336442488584	0.00577924886504	-0.00332651138368
C	-7.86351022638592	-2.47890838845801	-0.03537437183096
H	-3.38515197699308	-7.10947045061764	-0.10641371026071
H	-5.52755266360533	-5.87004695657871	-0.09393581190147
H	-7.67297043448884	-4.63699790037736	-0.07466469985704
H	-9.81965097494122	-3.40120446884992	-0.06027123090995
H	-11.96524222719091	-2.16465576014361	-0.04643812831807
H	-13.20502662114001	-0.00905256810508	-0.03407006062347
H	-11.97740100801889	2.15343939735380	-0.01623693017368
H	-9.83858799273292	3.40213555407475	-0.01458253103037
H	-7.69862529435923	4.64964752391915	-0.01795188937146
H	-5.56147539224763	5.89484340413037	-0.03533454151292
H	-3.42667421566359	7.14730203185570	-0.05057766008036
H	-0.95704491223921	7.15286057252882	-0.06595327833862
H	1.18279960605988	5.91482365372965	-0.08559216641765
H	3.32835109183394	4.68011980025423	-0.11674955944505
H	5.47086243236480	3.44604992979242	-0.14587039290731

H	7.61835985485975	2.21016297007448	-0.17576406897258
H	8.85833656247019	0.05460731082453	-0.19667179012875
H	7.63076800797484	-2.10813566608662	-0.18219777281227
H	5.49044992846156	-3.35653507179088	-0.15677896288705
H	3.35500776828587	-4.60289372820539	-0.13466058425360
H	1.21647491576495	-5.84949831828737	-0.11391487183199
H	-0.91556985975211	-7.09994960263288	-0.10983474542660
Pd	-3.62042001152654	-0.05889129672776	2.24269507354529
H	-3.94878668741321	0.24354779518204	3.89400096719969
H	-3.33415521564083	-0.36181062206420	3.90428762823576

Nb-G-H_2

C	-7.21372565592992	3.70753396116869	-0.05494507749737
C	-7.21274874607726	1.24595957578604	-0.05908043814792
C	-7.20187543518926	-1.22608309858539	-0.04236414193965
C	-7.18518059138873	-3.68675652796870	-0.01680838151463
C	-2.88195927385423	-6.15555002998225	-0.02660706009021
C	-2.94386469965283	3.73416252076216	-0.02845349195790
C	-2.94970749204117	1.26752493704037	-0.08042311144611
C	-2.93542096349024	-1.21367960350131	-0.04628593000939
C	-2.92381492611580	6.20411739097637	-0.00234285461830
C	-2.91670711396086	-3.68375632100415	-0.03098869755985
C	1.33864418288520	3.76094730221089	-0.03613788939899
C	1.34217727537235	1.27896382770202	-0.07340164732063
C	1.35096349409033	-1.20460810284795	-0.10953270536382
C	1.36519119335114	-3.68834687781452	-0.12104263550240
C	-11.48737072459177	1.20767783179786	-0.05253755574893
C	5.60867352678106	1.29691038787962	-0.06728387562207
C	-11.47787841525938	-1.22017975675900	-0.03663223468885
C	5.61689052235034	-1.19369137644290	-0.10262903003400
C	-5.80718095420137	3.73586603552820	-0.04888243727768
C	-5.79405359493131	1.25318863827299	-0.07975144255471
C	-5.78398683471259	-1.22256751357717	-0.05669668031318
C	-5.77827219701678	-3.70379394834868	-0.01580932279727
C	-1.51803232264545	-6.15475618919895	-0.05445548971789
C	-1.52286072122559	3.73858268298885	-0.03872119777538
C	-1.51225827895976	1.27116266408960	-0.06245227653771
C	-1.49866836759640	-1.21667593188989	-0.09885788036871
C	-1.55934619468498	6.21017328377060	0.00059680573131
C	-1.49547704858456	-3.68343924240654	-0.07652284106989
C	2.74557145830175	3.74083140829585	-0.03512631930923
C	2.75808977114637	1.27992723410930	-0.07886816201990
C	2.76616756780188	-1.19582530580474	-0.11428262548168
C	2.77160536479765	-3.65849595536534	-0.12690664267181
C	-10.06338217838209	1.24285834809168	-0.05630749198579
C	7.03294514945686	1.27001663186403	-0.06654441186310
C	-10.05392557277019	-1.24512437581227	-0.03991003987190
C	7.04047579061402	-1.15802033292028	-0.10110099170916
C	-5.08268999792466	2.49187385678482	-0.05744003658466
C	-5.08232843656060	0.01810127796800	-0.07751277054072
C	-5.06188691321849	-2.45580784533728	-0.03001923108078
C	-5.06752418805314	4.94996888622082	-0.03029563524072
C	-5.03169736667419	-4.91338817593363	-0.00574845691931
C	-0.79662676134222	2.51795723709943	-0.04286661671991
C	-0.78326827015717	0.03215311664871	-0.16618339614106
C	-0.77573064838685	-2.46025521392483	-0.09452008218424

C	-0.79890587587824	4.98430372636211	-0.01579890870407
C	-0.76478365570480	-4.92458161799295	-0.07998708432329
C	3.47426965614158	2.53253732130363	-0.05189928694297
C	3.47685685720950	0.04442593945861	-0.08440762934140
C	3.49124829186048	-2.44416973474299	-0.11945883498107
C	-9.34866901173609	2.45770808164924	-0.05982225181140
C	-9.34269028782911	0.00161402581843	-0.05200587434803
C	7.72584008150773	0.05828565391040	-0.08333465274627
C	-9.32992788621201	-2.45447426285422	-0.02892545893609
C	-3.66427217377251	2.50520921643609	-0.06108259246116
C	-3.66306740196584	0.02563139969083	-0.10452451050021
C	-3.64429189476489	-2.45965476471276	-0.03090944687901
C	-3.67450102277983	4.97195062310029	-0.02127711775228
C	-3.63933192079270	-4.92718734334793	-0.01993253713170
C	0.62191224302985	2.51325817991532	-0.05768868491774
C	0.63776801213500	0.03500720709575	-0.11119496217025
C	0.64056891233755	-2.44527670050606	-0.12276518093456
C	0.59231872728874	4.97174164128667	-0.01507246675069
C	0.62646385883108	-4.90374645973661	-0.10559561830449
C	4.88798350035435	2.50564501018245	-0.04982971824655
C	-12.17210434504783	-0.00877709127599	-0.04353468634827
C	4.89476917153396	0.04912968610152	-0.08605791016002
C	4.90407584681964	-2.40761223237846	-0.11810145316862
C	-7.93628422619162	2.49339697444884	-0.05994301787337
C	-7.92111710396130	0.00745426816606	-0.06336156352947
C	-7.91705907280398	-2.47891980087953	-0.02905656005675
H	-3.43262663451287	-7.09920713396508	-0.01044344865075
H	-5.57382783020572	-5.86265251941667	0.00618084679683
H	-7.72844495602839	-4.63481662838219	-0.00772382684234
H	-9.87619637003878	-3.40096246858878	-0.01878824269562
H	-12.02164012220567	-2.16741956768838	-0.02755021925451
H	-13.26427453911962	-0.01304084491362	-0.04054207695988
H	-12.03800652595448	2.15096410886509	-0.05566512455755
H	-9.90249255663795	3.39991360066227	-0.06019721266232
H	-7.76485041147657	4.65110318954384	-0.05141967339809
H	-5.61527196634069	5.89602173670947	-0.02329014081872
H	-3.47918423460364	7.14513149511912	0.00831491695453
H	-1.01176938032220	7.15564142062495	0.01489391148598
H	1.13488628021295	5.92051858159339	0.00336436103650
H	3.29089321883339	4.68757630149511	-0.01810963233620
H	5.43552859206253	3.45129880330222	-0.03384047045084
H	7.57774467408495	2.21654613057836	-0.05226646456495
H	8.81799127819826	0.06169342772926	-0.08221397617243
H	7.59096908306848	-2.10128885241908	-0.11353566537275
H	5.45799080435032	-3.34965337499655	-0.12686918934333
H	3.32407077518097	-4.60120728877130	-0.12846698224550
H	1.17456931511459	-5.84947109175029	-0.10508049800235
H	-0.96549010436152	-7.09739025038026	-0.05880884575952
Nb	-1.81717985329857	-0.10234941785090	2.00786858663972
H	-3.47006074307666	-0.07290630159933	2.64189413452690
H	-0.76464148390012	-0.45269931363045	3.43204537343307

Nb-G-2H_2

C	-7.19987302198253	3.68991054836824	0.03623965653044
C	-7.20552882818165	1.22514810314495	-0.01917987076029
C	-7.19792259322926	-1.24629251842536	-0.04470792903500

C	-7.17355748709960	-3.71124997142651	-0.04384200679988
C	-2.87632845528692	-6.18319992366550	0.05508685428931
C	-2.93678170940000	3.71612387846082	0.14202963159751
C	-2.94779219811046	1.24353332676353	0.08609389579272
C	-2.93921897913574	-1.23590798114313	0.06465708062521
C	-2.91991250894341	6.18981898968168	0.19384083494450
C	-2.90974665085413	-3.70873918154462	0.06652646255466
C	1.34295901578976	3.74463654613239	0.05571597605680
C	1.34997597784662	1.25756339785961	-0.01753037289943
C	1.35869769603421	-1.21874122464244	-0.04198225810962
C	1.36946089914976	-3.70563582967522	-0.02431756555089
C	-11.47650381319171	1.19066290459454	-0.10279047782563
C	5.61609704163269	1.28034907736152	-0.10747775478608
C	-11.46861696245773	-1.23901005731121	-0.13030993285112
C	5.62488956800328	-1.20963477030079	-0.13582287031734
C	-5.80185529956829	3.71970588106514	0.07250759121770
C	-5.79119932045539	1.23299693722893	-0.00958966753520
C	-5.78250149895035	-1.24432484000755	-0.03277008416394
C	-5.77582400000407	-3.73095167730837	-0.00580539037248
C	-1.51251898309539	-6.17827761052357	0.05199699468777
C	-1.52201895495910	3.72107931339700	0.12456487700041
C	-1.49388795650964	1.24721253363374	0.08141473970273
C	-1.48516898690775	-1.22934148186907	0.05937760375229
C	-1.55600075092173	6.19443823058604	0.19121725884894
C	-1.49647732794212	-3.70347073323240	0.04857451659840
C	2.74063473534338	3.72575921012871	0.01556656734243
C	2.76347544109177	1.26050538359790	-0.05552676086283
C	2.77334682516416	-1.21098771048426	-0.08131650013414
C	2.76643601222054	-3.67700027489625	-0.06628850003164
C	-10.05755214587444	1.22514433574973	-0.07529347306037
C	7.03419692406926	1.25605494204583	-0.14171981661479
C	-10.04888521651242	-1.26497141112844	-0.10304462515569
C	7.04347496797704	-1.17382364934192	-0.16979793210031
C	-5.07187515080819	2.47453349900047	0.05301023385549
C	-5.08568549191208	-0.00363044922280	-0.01542565239521
C	-5.05481146384554	-2.48067573989323	0.00402990050838
C	-5.06169968578973	4.93830567586577	0.12912732888753
C	-5.02616882592251	-4.94521098185369	0.02180941304927
C	-0.78253578232293	2.49607100271106	0.09814580502975
C	-0.77386270747370	0.01140806880753	-0.03016108976779
C	-0.76515583188717	-2.47199803059669	0.04991213034418
C	-0.79549828871960	4.96773252290101	0.14849157895285
C	-0.76073380293279	-4.94553238699287	0.04081196645555
C	3.48230585930590	2.51284157972276	-0.03448377218703
C	3.48122520246766	0.02716812735612	-0.07417603330131
C	3.50048440378214	-2.45821168333168	-0.08918115990650
C	-9.33480790925173	2.44187176300545	-0.04004172819139
C	-9.33647579698286	-0.01758510573748	-0.07879567134787
C	7.72656775807962	0.04354906605790	-0.17320983419990
C	-9.31825838161192	-2.47708007632027	-0.09453867573462
C	-3.66660138617231	2.48630509727381	0.09645415731069
C	-3.65995364635048	0.00126155425627	-0.01516778008059
C	-3.64895572602816	-2.48235530953545	0.05005168661594
C	-3.67212848243053	4.95786530244155	0.15528644814347
C	-3.63694610258600	-4.95581531707145	0.04854637078911
C	0.62074290222788	2.49490088857342	0.03685064610835
C	0.65359965490741	0.01654733964820	-0.03409527780853
C	0.63858110923812	-2.46089109198268	-0.01197770336266

C	0.59410242477266	4.95765680722681	0.11638443315533
C	0.62830812734663	-4.92490735172440	0.00737090118493
C	4.88367053465575	2.49260252106149	-0.06834564977134
C	-12.16052216063695	-0.02652437515185	-0.13077280472872
C	4.90382876717997	0.03262464233579	-0.10845034280048
C	4.90179525652127	-2.42749714689793	-0.12405156925042
C	-7.93188977531303	2.47226670262395	-0.00918365648493
C	-7.91425878529014	-0.01290955939761	-0.06186586192023
C	-7.91509345705925	-2.49822881611674	-0.06257283167879
H	-3.42485791684830	-7.12832426673904	0.05444460034667
H	-5.56841286691602	-5.89440800117553	0.01224936627437
H	-7.71928391868217	-4.65842056016493	-0.05630573281041
H	-9.86517449998555	-3.42328418730553	-0.11139273646705
H	-12.01398703766482	-2.18512463580946	-0.14998861851355
H	-13.25256746129766	-0.02989379293812	-0.15204158848475
H	-12.02813530903891	2.13334289538441	-0.10095411265441
H	-9.88825175701001	3.38443598764209	-0.03484680193372
H	-7.75292837155129	4.63285870772248	0.04666975507311
H	-5.61084921142575	5.88341307917313	0.14394946221429
H	-3.47484827917749	7.13088547475028	0.21828047975425
H	-1.00738711382327	7.13923298938761	0.21491112106765
H	1.13658704252637	5.90664778843118	0.13417431054322
H	3.28673256242220	4.67271344392381	0.02808533008559
H	5.43076968682091	3.43887554515452	-0.06166180414280
H	7.57941542459919	2.20246732206313	-0.14234020529687
H	8.81853031367065	0.04795941956918	-0.19975765564159
H	7.59544816533970	-2.11604726001222	-0.19236018241030
H	5.45550097405987	-3.36978342386993	-0.13990295041965
H	3.31853195978343	-4.62047073307621	-0.07739786161900
H	1.17712791266780	-5.87041093419248	0.00008415745622
H	-0.95721777478356	-7.11944416887983	0.05023329539534
Nb	-2.20651734164794	0.02030402022212	1.92273962718174
H	-0.57234852529669	0.14834063282831	2.83220385293390
H	-1.17490024826424	0.15760109938760	3.51571571257240
H	-3.82413534572117	0.16497952686247	2.86406429371625
H	-3.20750587666027	0.16841259974477	3.53466222773137

Ni-G-H_2

C	-7.16870099641782	3.72106669556907	0.04728596581944
C	-7.17490359641795	1.25941441027261	0.02306349334663
C	-7.16632881171572	-1.21319613488063	0.00142002567474
C	-7.15212835899338	-3.67634737629249	-0.01274170902621
C	-2.85722742152940	-6.15294822757264	-0.01515064698535
C	-2.90578660132182	3.74224586480720	0.04982658188328
C	-2.90751944000020	1.27454060546670	0.00868344365671
C	-2.90643416876626	-1.20716489075809	0.01645422864067
C	-2.88866742596735	6.21772480186808	0.07716918465684
C	-2.88849323968365	-3.67832560967472	-0.00224419982597
C	1.36665871499043	3.76389085786386	0.00847367097577
C	1.36893190391746	1.28458028255374	-0.03045462811140
C	1.37464242919061	-1.19449662653129	-0.04282702145626
C	1.38761716746287	-3.67813630496594	-0.04311403585576
C	-11.44804134178998	1.22658295291432	0.02349062577053
C	5.63655921179470	1.30118187148620	-0.05497861545101
C	-11.44118218367265	-1.20181497818468	0.00303470220631
C	5.64287293974976	-1.18822405738061	-0.07351082208119

C	-5.76797648372209	3.74577774275342	0.04783862415561
C	-5.75796319380267	1.26618655359582	0.00852051380595
C	-5.75110203503914	-1.21284173304988	-0.01111435822579
C	-5.75079125533794	-3.69647081409060	-0.00853850421302
C	-1.49314633902881	-6.14932261697340	-0.02205209748081
C	-1.49541368537530	3.74590647317481	0.02635850225518
C	-1.48080802347147	1.27814292371377	-0.00812937550631
C	-1.46853251450815	-1.20285597425871	0.02067500742907
C	-1.52474745047627	6.22112649804160	0.06974710928723
C	-1.47429756023910	-3.67441854659756	-0.01302561874037
C	2.76754568587943	3.74668561760918	-0.00539188766339
C	2.78591641418669	1.28514309782247	-0.02717049272811
C	2.78996099292385	-1.18757460697984	-0.04635366829641
C	2.78859992889722	-3.65090096287921	-0.06022553766473
C	-10.02559950754042	1.26091639529169	0.02176726443309
C	7.05905411336070	1.27406531284969	-0.06561535759353
C	-10.01932763481242	-1.22859983748746	0.00079538147114
C	7.06453167965183	-1.15424726187080	-0.08383735558039
C	-5.04177358388750	2.50054311458446	0.02830729887713
C	-5.04568195444978	0.02980836136862	0.00011061327920
C	-5.03065600504485	-2.44771693415549	-0.00568143787097
C	-5.02806871086840	4.96468645487216	0.06428678314336
C	-5.00493178392374	-4.91155223039710	-0.00871745934796
C	-0.76904473089174	2.51541730571485	0.01588218232227
C	-0.75247121542545	0.04547777134662	-0.04547408837157
C	-0.75136346974759	-2.44596233022198	-0.01436211728338
C	-0.76750680278645	4.99281741713355	0.04554456727940
C	-0.74094573888520	-4.91731740532768	-0.02293715749741
C	3.50304562603451	2.53534611994378	-0.02515929363417
C	3.50184453516862	0.05102338631519	-0.05611501902708
C	3.51501972686672	-2.43440894257300	-0.06186993888376
C	-9.30674615404875	2.47521444940211	0.03323194917562
C	-9.30462029726869	0.01803242002126	0.00945604275677
C	7.75102185286542	0.06186255544424	-0.08004053446059
C	-9.29361440021192	-2.43926523050398	-0.00768579083193
C	-3.62611588036786	2.50874360924896	0.02037253235345
C	-3.63004708000258	0.03792073295353	-0.02688560390561
C	-3.61800115422997	-2.45348172688622	-0.00657702620280
C	-3.63991432514056	4.98558225393044	0.06291386246210
C	-3.61570709948181	-4.92478291183677	-0.00884040311211
C	0.64683982723152	2.51513814707000	-0.00357254769696
C	0.66274681961902	0.04444605112820	-0.03117251612861
C	0.66076440753155	-2.43313291386004	-0.03326290871307
C	0.62067358114471	4.97894296861756	0.03377230368380
C	0.64811700955260	-4.89711579698531	-0.03567415801875
C	4.91167759089304	2.51182175865134	-0.03813612931805
C	-12.13375868976500	0.01083525724600	0.01392794673480
C	4.92183301477794	0.05466834690060	-0.05957044536128
C	4.92341888859649	-2.40265300975906	-0.07481235171104
C	-7.89821723893548	2.50588978367550	0.03336230501841
C	-7.88469273060385	0.02173367427575	0.00008578004532
C	-7.88491086693455	-2.46373091271886	-0.00790070526004
H	-3.40680188253110	-7.09748041430431	-0.01680183332293
H	-5.54955621884748	-5.85948986671379	-0.01219955549204
H	-7.69916171848163	-4.62265725873741	-0.01719871617081
H	-9.84098688343860	-3.38535078888095	-0.01398348399248
H	-11.98659522871625	-2.14813153821489	-0.00360000287595
H	-13.22593362976382	0.00773740963443	0.01533535301492

H	-11.99823808747588	2.17009882855513	0.03270997074201
H	-9.85885451677653	3.41851078733876	0.04181136800324
H	-7.71846842658235	4.66571422378852	0.05888715623686
H	-5.57775023643068	5.90969257762726	0.07637690492625
H	-3.44363171449700	7.15895519063836	0.09303605906364
H	-0.97429990113652	7.16507490107152	0.08085546064323
H	1.16560002892714	5.92671890394247	0.04496708712389
H	3.31240929935927	4.69421814405425	0.00036823532576
H	5.45905913544245	3.45790717070059	-0.03545789700752
H	7.60452843468197	2.22036767964322	-0.06204679823137
H	8.84315384661982	0.06446818986824	-0.08815608992114
H	7.61462182356924	-2.09782365441084	-0.09443767731636
H	5.47558754201010	-3.34590522069759	-0.08579684281734
H	3.34047361397799	-4.59434942122806	-0.06947984183985
H	1.19749877231361	-5.84227057146117	-0.04264512950110
H	-0.93861176432481	-7.09093728838124	-0.02880162749102
Ni	-2.19554994980132	-0.47987401306429	1.85457787884505
H	-2.12367028668258	-0.11330436393771	3.40601824778398
H	-2.06291083117075	-0.99311359867542	3.32432444679441

Ni-G-2H_2

C	-7.14795363027679	3.65519109105002	-0.00163351811291
C	-7.14408100342324	1.19225730704688	0.04038299967631
C	-7.13168883941382	-1.27860898838551	0.07494138048826
C	-7.10387215371329	-3.73898992933374	0.05589755505620
C	-2.80732969845035	-6.20381046198223	-0.06477938273749
C	-2.88778114706107	3.69067547245612	0.14344713242262
C	-2.87392812412760	1.22142061239684	0.19933239704889
C	-2.85470043680569	-1.26651826119185	0.08962544469033
C	-2.87567456255217	6.16535090906989	0.13423121754017
C	-2.84210642364666	-3.72949568876951	0.00400959531374
C	1.38542603880152	3.72052858141049	0.10868185063188
C	1.39203442546799	1.24054152411370	0.04091985342011
C	1.40458674218718	-1.23296487141103	-0.05755008941920
C	1.42429898564177	-3.71230930945955	-0.13903825076459
C	-11.41823242610960	1.14564314372557	-0.03162167542111
C	5.66104263772199	1.27321511281631	-0.07404546400478
C	-11.40557348072573	-1.28242931724335	0.00540923986433
C	5.67372987510281	-1.21380488276447	-0.16467390522301
C	-5.74748847050452	3.68576769064694	0.03624615662406
C	-5.72962942238244	1.20407369197758	0.06496057745024
C	-5.71427812437066	-1.27362523769793	0.09132499325354
C	-5.70195182017842	-3.75188213079496	0.05261646684124
C	-1.44390237594633	-6.19515416202106	-0.10610838350433
C	-1.47608734253382	3.69344514596840	0.14842550222231
C	-1.44569846087858	1.22242115063465	0.12586610625845
C	-1.43909436791227	-1.25118344308010	0.00830290961938
C	-1.51187815920077	6.16893682107326	0.15863249640656
C	-1.43350510212973	-3.72014092929002	-0.05111385573820
C	2.78613632407133	3.70647377420415	0.07810728322212
C	2.80901898857536	1.24654665709716	-0.00752136597168
C	2.82123660600284	-1.22101563757205	-0.10034129398286
C	2.82442649626739	-3.68144617128780	-0.17504041340468
C	-9.99666278130196	1.18413200931780	-0.01063982170965
C	7.08327500638016	1.25171578924566	-0.11040261901985
C	-9.98305839167527	-1.30474832577863	0.02666651530839

C	7.09554832900768	-1.17488562582428	-0.19864931152607
C	-5.01868215801833	2.44424709540035	0.06410876913649
C	-5.01374963246923	-0.03176680483507	0.13779204347122
C	-4.98619930610922	-2.50192626122948	0.07602875637084
C	-5.01229542047759	4.90815318620179	0.06440545725882
C	-4.95485290594056	-4.96627285729110	0.01678819500540
C	-0.74506858216464	2.46237629895562	0.14073753169598
C	-0.73137600905038	-0.00928798830532	0.04382335529327
C	-0.71989549175491	-2.48048940212979	-0.03964834521734
C	-0.75238114554979	4.94124935352966	0.15410856149256
C	-0.69662761236620	-4.96047163562675	-0.10179263474421
C	3.52309501576627	2.49763649502313	0.02262392078139
C	3.52962725250958	0.01701095844307	-0.06346493924731
C	3.54865934139974	-2.46386451052993	-0.15621180045269
C	-9.28005073150560	2.40063269919917	-0.02293800489460
C	-9.27246001102928	-0.05607159167367	0.02294272985243
C	7.77847829870676	0.04259315744566	-0.17163917449667
C	-9.25331073127702	-2.51265265346895	0.04383721361935
C	-3.60594908975625	2.45901011259003	0.12905902732032
C	-3.58598586157896	-0.03252566808479	0.14782469203213
C	-3.56882151572164	-2.50030168190158	0.04900268523781
C	-3.62490212069817	4.93242210363429	0.11270434435594
C	-3.56711047466682	-4.97809405365971	-0.01326381125116
C	0.67012756423960	2.46926834590603	0.09119119716126
C	0.68333855340444	0.00009087352779	-0.00731469830956
C	0.69543617821790	-2.47003815056987	-0.08836081897104
C	0.63672132312694	4.93289584548389	0.14548299013088
C	0.69071344673141	-4.93495090276725	-0.14719965968997
C	4.93182978304109	2.47992722851891	-0.01246570292054
C	-12.10113284476032	-0.07242739193355	-0.02211494034391
C	4.94998841817281	0.02545026598640	-0.10219582384112
C	4.95710669455489	-2.42936459329593	-0.18929629270399
C	-7.87223129936788	2.43668666349985	0.00079956471745
C	-7.85222983754609	-0.04797756690985	0.04590231519591
C	-7.84405337898348	-2.53085791460255	0.06027596983142
H	-3.35469155642409	-7.14959936945494	-0.07218330232174
H	-5.49942067226907	-5.91421082585001	0.00218978118700
H	-7.64530259061066	-4.68845087591107	0.04211766575017
H	-9.79699782771798	-3.46086336061259	0.03824406276819
H	-11.94802328807115	-2.23046059398757	0.00918707389062
H	-13.19315213385744	-0.07834968919328	-0.03849998612312
H	-11.97102185740430	2.08739606746178	-0.05615178693793
H	-9.83499049542384	3.34196855146106	-0.04980873336536
H	-7.70176895135300	4.59727276199897	-0.02427927951931
H	-5.56475865068468	5.85131934674040	0.04457792090004
H	-3.43089710217450	7.10654891637242	0.12671085024051
H	-0.96238200957000	7.11346355925317	0.17118635347155
H	1.17864527842334	5.88236558790625	0.15332126346028
H	3.32954838773422	4.65482845684299	0.09330880718198
H	5.47572939381074	3.42783674765030	0.00780472129572
H	7.62582992030879	2.19946857002666	-0.08971686041187
H	8.87028456833104	0.04900787028076	-0.19881381682831
H	7.64776971766431	-2.11606587281591	-0.24636100289781
H	5.51065956963561	-3.37078798320412	-0.23406106784381
H	3.37786413347269	-4.62324174880611	-0.21525201437480
H	1.24400335939982	-5.87702034802055	-0.18678715018968
H	-0.88641971670170	-7.13420977003567	-0.14597819895029
Ni	-3.24480512925288	0.53053770771207	2.16026223659818

H	-1.87832314166547	-0.40152845377870	2.56259531574425
H	-2.12160410880365	-0.08685147545119	3.29551053410299
H	-4.60306629664013	1.46971249495343	2.58980092782868
H	-4.30446821914063	1.21668156356760	3.32427468964828

W-G (mul 7)

C	-7.18340554862151	3.70349026377796	-0.00550259633569
C	-7.18296853056255	1.24121449491772	-0.06006439318905
C	-7.17580491212473	-1.23519726873204	-0.07359452173402
C	-7.16124469108446	-3.69773617384237	-0.04442241005181
C	-2.85412480265351	-6.16279723873842	0.05898477056959
C	-2.91544529133491	3.72128259395945	0.04592118050184
C	-2.92464548732254	1.25027446733402	-0.02326362050526
C	-2.91743334629399	-1.21930505018292	-0.04199568998621
C	-2.89098855150500	6.19296613624330	0.12271908704207
C	-2.89294999444888	-3.69058579603264	0.00751110488212
C	1.37215196188966	3.75353074018791	-0.03895155693848
C	1.37771776193048	1.27079604493541	-0.12877857223640
C	1.38512636598533	-1.21320837663295	-0.13956769121847
C	1.39444752523011	-3.69682213686626	-0.07589948194233
C	-11.45700853366792	1.20465665159945	-0.07445585197916
C	5.64214031468645	1.28728373381340	-0.18287019678126
C	-11.44981703427790	-1.22390497673415	-0.08729791062028
C	5.64966659623980	-1.20398501317402	-0.19492527069696
C	-5.77724417460970	3.73222418274584	0.01198126898132
C	-5.76894047180657	1.24922151687003	-0.07459207878216
C	-5.76159919496000	-1.23462136129672	-0.08875999867508
C	-5.75491508828959	-3.71808649298551	-0.02732700283236
C	-1.49015599268133	-6.15882039677931	0.04978191835967
C	-1.48866581412424	3.72503197700896	0.05065506667641
C	-1.46521719180356	1.25489014485914	-0.03621897242401
C	-1.45816863076711	-1.21502265589624	-0.04560035666731
C	-1.52701624281384	6.19696945696351	0.11348052902109
C	-1.46636545538162	-3.68615766686189	0.01225496038636
C	2.77812562538011	3.73333677469700	-0.07665897506099
C	2.79189208494471	1.27118072810521	-0.13182664685861
C	2.79928129474734	-1.20521706013819	-0.14380519794955
C	2.80016439828055	-3.66789862999780	-0.11313718313016
C	-10.03387407470569	1.24021339287322	-0.07024841371928
C	7.06521140450941	1.26034322585321	-0.20798860124764
C	-10.02643489186076	-1.25108024542047	-0.08344663565225
C	7.07273454454908	-1.16826212434338	-0.21975669045123
C	-5.05211067493666	2.48837761502013	-0.02483114742213
C	-5.06220749732972	0.00953858059186	-0.11328076212318
C	-5.03742652577797	-2.46964283605638	-0.05231446776171
C	-5.03729600069761	4.94567083558886	0.06632557556093
C	-5.00759028884007	-4.92755634893381	0.01485354632882
C	-0.75825423200348	2.50916371392118	-0.01449108838460
C	-0.73792098608629	0.02291625147309	-0.17157471301818
C	-0.74322949633023	-2.46534385995113	-0.03826114551780
C	-0.76678457816981	4.97049050938259	0.06326949545457
C	-0.73710954491791	-4.92748374274824	0.01251893751096
C	3.50888599558556	2.52397594215165	-0.12183705954309
C	3.50908850022795	0.03524277205219	-0.17017869879502
C	3.52381346324821	-2.45377929035022	-0.14602424289711
C	-9.31849250008691	2.45463350533351	-0.05000718785878

C	-9.31208221491858	-0.00327230137325	-0.08021624267214
C	7.75762909089963	0.04821726090629	-0.22745568375532
C	-9.30393373082620	-2.46135451591626	-0.07595443266742
C	-3.63946153705063	2.50046584455605	0.01503023948211
C	-3.64666048597606	0.01365391485281	-0.15376077455614
C	-3.62430474441650	-2.47354033262143	-0.01301183585881
C	-3.64459841869102	4.96207812852188	0.08205344214324
C	-3.61485401577119	-4.93591351614070	0.03071712241103
C	0.65433446940315	2.50534301462726	-0.06587781106039
C	0.67803021832099	0.02675130045277	-0.15140215886514
C	0.66903426526585	-2.45268942580353	-0.09012066277701
C	0.62563623025574	4.96243628753874	0.02638138598630
C	0.65519486004403	-4.91081774396440	-0.02341492738327
C	4.91987527027061	2.49735446621359	-0.15239166951069
C	-12.14216501863313	-0.01168840074672	-0.08432321985428
C	4.92790540347867	0.03949484185666	-0.18196770584329
C	4.93459604871843	-2.41858519279988	-0.17633607850581
C	-7.90743301930293	2.48968128899861	-0.03977513982694
C	-7.89324385954490	0.00096967170036	-0.08924590979042
C	-7.89262771373879	-2.48807726336588	-0.06624013860853
H	-3.40255180578586	-7.10769066992644	0.08199681601188
H	-5.54798334326800	-5.87758121173849	0.03503685549415
H	-7.70717522348077	-4.64448059637999	-0.03443318505139
H	-9.85225440355923	-3.40683135555836	-0.07423383284018
H	-11.99485039339566	-2.17043188317956	-0.09099902477224
H	-13.23421968946556	-0.01499330271111	-0.08731272390323
H	-12.00763060558187	2.14792939071683	-0.06817476651393
H	-9.87239969737313	3.39678144784592	-0.03853826527873
H	-7.73507528476949	4.64675493394377	0.01389222754210
H	-5.58331841791377	5.89220504644845	0.09635101658559
H	-3.44482851626261	7.13440655952649	0.15557417900072
H	-0.97834856191918	7.14170917020170	0.13725083041648
H	1.16621906970302	5.91239974045079	0.04424833528367
H	3.32424450723371	4.67995782469463	-0.06484192493330
H	5.46818904803936	3.44284608562457	-0.14891658446187
H	7.61004877606013	2.20698626561025	-0.21014048119284
H	8.84966059217605	0.05163261136737	-0.24658021809362
H	7.62348960810813	-2.11139554419957	-0.23109432689018
H	5.48861059251419	-3.36072024204378	-0.18220892913686
H	3.35197455694821	-4.61127826131927	-0.11078704499577
H	1.20147658853453	-5.85763968261887	-0.01513468437552
H	-0.93598091015632	-7.10052871102777	0.06389896559958
W	-2.17946355872768	0.01056108721486	1.77891906773025

Cu-G (mul 4)

C	-7.17309905722536	3.74975344760233	-0.05751627045189
C	-7.17985645966022	1.29064237601788	-0.04861516497736
C	-7.17518598164232	-1.17926381240720	-0.03759082480473
C	-7.16266057902642	-3.63833052558692	-0.02273032299280
C	-2.87360318440592	-6.11934307067743	-0.01586371716437
C	-2.91288173749928	3.76758665669804	-0.08272151513370
C	-2.91199959926148	1.30165786763778	-0.08501623103980
C	-2.90638440721971	-1.17744925160680	-0.05766901898919
C	-2.89325381450638	6.24377260619066	-0.08894270903219
C	-2.90159457964506	-3.64453243952209	-0.04834035078997

C	1.35571028166281	3.78404849426799	-0.11115346935993
C	1.35261522097524	1.30555843826337	-0.11174565714395
C	1.35705991831069	-1.16836321948122	-0.09573353578423
C	1.36926549215328	-3.64785447166771	-0.06154292735857
C	-11.45639587319368	1.26376858690198	-0.00917975889825
C	5.62409473192550	1.32107250059107	-0.11462303677146
C	-11.45250669000077	-1.16437878678281	0.00207924047204
C	5.62863671132601	-1.16819995920405	-0.09694758432016
C	-5.77127268162923	3.77327699328467	-0.06721926780127
C	-5.76154595667611	1.29539426471100	-0.07793858489101
C	-5.75750665897177	-1.17953556450667	-0.06574288380369
C	-5.76031205987200	-3.65855615546869	-0.03130806003668
C	-1.50985913810219	-6.11608324863035	-0.01839429745862
C	-1.50243659697298	3.76887544518951	-0.10332895470940
C	-1.49376199205696	1.29984228907857	-0.10318924516765
C	-1.48405185629373	-1.17216007965806	-0.09421996683662
C	-1.52964089429078	6.24538889622122	-0.09677968506946
C	-1.48946321189061	-3.64099412749206	-0.05520330704897
C	2.75740538935590	3.76479994628404	-0.11856191961976
C	2.77142413975052	1.30579420148962	-0.10550615355738
C	2.77582556286064	-1.16333184601658	-0.08821869094714
C	2.77138323977671	-3.62275078142981	-0.06729460234709
C	-10.03155541683588	1.29633374239514	-0.02447680881870
C	7.04909173963223	1.29295114825762	-0.11767084181113
C	-10.02787455364736	-1.19318504372284	-0.01295785744979
C	7.05334964198106	-1.13510723398789	-0.10038688100055
C	-5.04766912105561	2.52818579449154	-0.07069836121676
C	-5.04781198349042	0.06007236269978	-0.07395254511805
C	-5.04089868382957	-2.41146808302814	-0.05080866647798
C	-5.03254801385623	4.99232852360572	-0.07409895863414
C	-5.01776216246535	-4.87467644413255	-0.02121258021498
C	-0.78220336144200	2.53590114045057	-0.09619501378717
C	-0.77625363193956	0.06528627394376	-0.10036250171634
C	-0.77214961500824	-2.40670164103506	-0.07110760785796
C	-0.77423027291807	5.01580916790291	-0.10082273040668
C	-0.75798328705184	-4.88435547719377	-0.03697067173503
C	3.48876033745056	2.55380832570766	-0.11603182676537
C	3.48851394460218	0.07238062554493	-0.10404168817455
C	3.49753057409754	-2.40901634389086	-0.08156217092370
C	-9.31272555336384	2.50750185977569	-0.03560717374732
C	-9.31215951820213	0.05271106335200	-0.02722370938855
C	7.74021039287508	0.08020908290482	-0.11064157163836
C	-9.30528486559099	-2.40214465077225	-0.01296451311630
C	-3.62933546347048	2.53339587634216	-0.09157498365931
C	-3.62935541392748	0.06506938780215	-0.08654132992084
C	-3.62313472551310	-2.41610768700096	-0.06681223832114
C	-3.64508097189004	5.01209769878762	-0.08352018727266
C	-3.62971865449069	-4.89051259027142	-0.02985017478579
C	0.63605296903142	2.53643141278017	-0.10362421359479
C	0.64009996528232	0.06760740339651	-0.08633268570557
C	0.64563511567991	-2.40218761590840	-0.07162268167737
C	0.61331726254338	5.00072287131539	-0.10890168121019
C	0.63064165994412	-4.86585440291465	-0.04296784529405
C	4.90098065733819	2.52975632040900	-0.12045437569750
C	-12.14336106454842	0.04878766749276	0.00352823338945
C	4.90876946063646	0.07518603929002	-0.10406387594439
C	4.90976365108035	-2.37943468017642	-0.08577252517496
C	-7.90081742928709	2.53651211936783	-0.04878156043567

C	-7.89229173084180	0.05473698206060	-0.04757667991146
C	-7.89289116677384	-2.42742869891808	-0.02609182628562
H	-3.42432917538447	-7.06301015177700	-0.00135238952908
H	-5.56473328110013	-5.82108859121309	-0.00537175517066
H	-7.70919860920764	-4.58477572394906	-0.01031175691524
H	-9.85216952556870	-3.34837237223560	-0.00196139561543
H	-11.99845568722237	-2.11026521399454	0.01297290220811
H	-13.23544704512359	0.04707701040557	0.01531978123667
H	-12.00495751744606	2.20818930042954	-0.00706853738074
H	-9.86253998512174	3.45211639196172	-0.03355761860860
H	-7.72215626523119	4.69484984588350	-0.05626625386162
H	-5.58259533703934	5.93712971900722	-0.07266632115639
H	-3.44750608763320	7.18550719763209	-0.08631531516788
H	-0.97773517665167	7.18850641700012	-0.09953181608712
H	1.16028500791498	5.94730264655663	-0.11126260676375
H	3.30359798259858	4.71156291773454	-0.12466681022776
H	5.44742969526632	3.47628841217494	-0.12786178049706
H	7.59464018746396	2.23909897866376	-0.12544794402631
H	8.83236104983714	0.08211284642891	-0.11310513294598
H	7.60228558194246	-2.07930428704633	-0.09471375786152
H	5.45975991486852	-3.32393996738340	-0.07978021854461
H	3.32148675055909	-4.56721967503039	-0.05976160574063
H	1.17978585232745	-5.81103721332008	-0.03239357635687
H	-0.95504047332028	-7.05741862401260	-0.00644261866418
Cu	-3.17024024551700	-0.60151183133073	2.38202231301312

Cr-G (mul 7)

C	-7.17054663982139	3.68827214770209	-0.03687216363158
C	-7.17366329943783	1.22745901238503	-0.05560451226639
C	-7.16589169965903	-1.24244327558690	-0.06749707755670
C	-7.14698910742207	-3.70257581626315	-0.07652836138354
C	-2.85661986417956	-6.17769173291821	-0.12954466644209
C	-2.90905615274242	3.71400609669816	-0.08372131271709
C	-2.91276778808298	1.24610138407061	-0.13892336758720
C	-2.90030056126893	-1.23345498167580	-0.14972592183585
C	-2.89447426641064	6.18971108168342	-0.03720263722582
C	-2.88638291597147	-3.70174391285376	-0.13125173142974
C	1.36131573606605	3.73735878154096	-0.09141823229474
C	1.36272088310330	1.25546902360871	-0.12591366271990
C	1.36991182771516	-1.22057559976037	-0.14565462301936
C	1.38407275579519	-3.69969857469074	-0.15305140517080
C	-11.44902385092595	1.19418264734829	0.01176372818081
C	5.63337834828159	1.27567446794505	-0.10816704842205
C	-11.44113984662576	-1.23472825492042	-0.00066190051477
C	5.64079106685077	-1.21505282916629	-0.13030188839150
C	-5.77084166657004	3.71451846303641	-0.05645927259004
C	-5.75656076824320	1.23484542700571	-0.09328501809488
C	-5.74903820247643	-1.24077056222364	-0.10641665628805
C	-5.74695799097505	-3.72001577320504	-0.09728131811548
C	-1.49303420032904	-6.17417958766896	-0.14076352928608
C	-1.50047865229672	3.71991375956658	-0.09614465489945
C	-1.48503386288179	1.25018629359662	-0.15380939520800
C	-1.47873825977041	-1.23134354762617	-0.17800749249134
C	-1.53085447811377	6.19433704374272	-0.04037349326509
C	-1.47781887207956	-3.69820728360836	-0.15849198541509
C	2.75983478764889	3.71865493944031	-0.08503090976462

C	2.77813505683829	1.25709674884088	-0.12669190767762
C	2.78761587902446	-1.21328323300139	-0.14897305720645
C	2.78297517676129	-3.67459629028717	-0.14954744955595
C	-10.02664893709409	1.22859688585731	-0.01177951429952
C	7.05312276703815	1.25044492509226	-0.10444159520981
C	-10.01914508024477	-1.26106372632669	-0.02442308397512
C	7.06303472146282	-1.17932463024956	-0.12610076864971
C	-5.04439051749459	2.47016517769342	-0.08483792029246
C	-5.04031812313803	-0.00110452827324	-0.10766324681332
C	-5.02759929592229	-2.47106947072658	-0.10736735986517
C	-5.03168178133692	4.93419266985308	-0.04628209327550
C	-5.00136041746456	-4.93545656698732	-0.10655252766156
C	-0.77109105247009	2.49035649337492	-0.11348127541437
C	-0.76306257581022	0.00882491776429	-0.15800365173100
C	-0.75905753456859	-2.46347419108838	-0.15714633840741
C	-0.77286545712815	4.96654536011825	-0.06629879998258
C	-0.74267181454048	-4.94143924797430	-0.14930682735635
C	3.49738047834944	2.50703657510680	-0.10163292954232
C	3.49779757875134	0.02358923908928	-0.12455609801201
C	3.51329571262283	-2.45916171169946	-0.14551924595287
C	-9.30680283334270	2.44212997664238	-0.01552582495554
C	-9.30516293407991	-0.01393504549764	-0.03081655914386
C	7.74566144041655	0.03684721956031	-0.11382485314354
C	-9.29172976148524	-2.47035948302350	-0.04055641358718
C	-3.62990529480001	2.48007023325980	-0.11906260919870
C	-3.62402651311629	0.00235031770064	-0.15046421841783
C	-3.61218134696623	-2.47045113084405	-0.13741192716405
C	-3.64442473613295	4.95678794676881	-0.05764644522451
C	-3.61396279577899	-4.94933246264259	-0.12404835482322
C	0.64093768610059	2.48798237739554	-0.12165146922548
C	0.65449379517080	0.01506840166296	-0.15610366839946
C	0.65627855906052	-2.45489626951753	-0.15838437249264
C	0.61446094506916	4.95231736424864	-0.06764169162143
C	0.64442390430605	-4.91930314185134	-0.15096139908896
C	4.90277598341753	2.48542642522393	-0.09652768041286
C	-12.13343085274545	-0.02218710804604	0.01665561447443
C	4.91872761640191	0.02816015591825	-0.12282068680804
C	4.92014589801813	-2.42837270351630	-0.13988128345649
C	-7.89859239824089	2.47259974205706	-0.03749291975602
C	-7.88451641489065	-0.00950336608481	-0.06097944822464
C	-7.88323737273826	-2.49218016069138	-0.06268515049683
H	-3.40728428781547	-7.12152693754856	-0.12307323033167
H	-5.54692205702363	-5.88284105660043	-0.09985674502443
H	-7.69163335137857	-4.65025589899463	-0.07064697393741
H	-9.83749884496377	-3.41730670255519	-0.03567523139517
H	-11.98613459305840	-2.18121536826703	0.00399531555108
H	-13.22549716979551	-0.02582280145045	0.03472607828930
H	-11.99933278672928	2.13746938428572	0.02627328065482
H	-9.85803165991592	3.38581801059887	-0.00059960175358
H	-7.72132566731385	4.63222403294039	-0.01854047694079
H	-5.58265172063456	5.87825086442080	-0.02588682282054
H	-3.45055943266820	7.13012973798622	-0.01743777366791
H	-0.98090876037088	7.13839402281538	-0.02281809663322
H	1.15987970153252	5.89957263757392	-0.04611373233133
H	3.30526717009199	4.66570367140290	-0.06417047580974
H	5.44832264641975	3.43244199027418	-0.08178295334299
H	7.59831004055528	2.19671025319652	-0.09395069250522
H	8.83798555006449	0.04109681791487	-0.11100514154154

H	7.61416688685445	-2.12219126344712	-0.13255062364287
H	5.47169194660535	-3.37197388476290	-0.14208589418231
H	3.33320397230754	-4.61900735845714	-0.14685363100239
H	1.19529197951443	-5.86366444098937	-0.14698977371090
H	-0.93782136606369	-7.11533568554537	-0.14193060109712
Cr	-2.06143301267042	0.30653845310606	2.20471235907025

Mo-G (mul OSS)

C	-7.17950434528854	3.70355616441546	0.00591253540153
C	-7.18460312799427	1.23747745729039	-0.03673290912102
C	-7.17707505865197	-1.23216112152763	-0.04934910102349
C	-7.15696003754674	-3.69847753592777	-0.03210313848890
C	-2.85495395823348	-6.17361423522345	-0.05594148661521
C	-2.91425040973843	3.73399718603361	-0.08212836672103
C	-2.91688288515132	1.26519169475700	-0.20164562016827
C	-2.90926299215265	-1.23235135219329	-0.21436781859113
C	-2.89256234408826	6.20475850968276	0.00823839271962
C	-2.89162062491181	-3.70221034017259	-0.12047945753642
C	1.36999610935855	3.75645542414962	-0.07215736612092
C	1.37844954728281	1.26481980235536	-0.13810561445647
C	1.38600470948665	-1.20669456038352	-0.15088396592089
C	1.39268805880215	-3.69889104114257	-0.11055435125471
C	-11.45630770439530	1.20322877769113	0.08601238266915
C	5.64649447302269	1.28510913346769	-0.05341838756373
C	-11.44890148359176	-1.22507694827399	0.07366259325432
C	5.65403306669050	-1.20192551058893	-0.06613454450045
C	-5.77564071946017	3.73411368598227	-0.02642076428833
C	-5.76992570803690	1.24279371626085	-0.09789634182242
C	-5.76239781450929	-1.22828728227746	-0.11059081087524
C	-5.75293374611836	-3.72020788838911	-0.06480695217442
C	-1.49013448943842	-6.16966979057462	-0.06592694703668
C	-1.49158487002666	3.73795006611582	-0.07949565771125
C	-1.47643251368336	1.26821602429937	-0.19099083098755
C	-1.46880354159953	-1.22682449221399	-0.20376482758857
C	-1.52774664311123	6.20917101108257	-0.00178674849205
C	-1.46895425744181	-3.69761179385957	-0.11788526170309
C	2.77411239203149	3.73413170718893	-0.05838429652077
C	2.79348106661499	1.26805599958344	-0.09742343920416
C	2.80104675975132	-1.20176642341056	-0.11012654911095
C	2.79666065414069	-3.66822679440008	-0.09642570726409
C	-10.03672361028775	1.23702624709739	0.04609730223454
C	7.06656091689851	1.26000346431686	-0.03226186358261
C	-10.02913425323756	-1.24986667219248	0.03343045427085
C	7.07393306472995	-1.16836437961666	-0.04468147632817
C	-5.04752064738358	2.49144025675646	-0.08124905974201
C	-5.06906650075528	0.00946067465273	-0.12154339046811
C	-5.03239078332034	-2.47264271247165	-0.10683741598575
C	-5.03832388144612	4.95187707905438	-0.00334871307143
C	-5.00825619618319	-4.93366602151903	-0.05440677803425
C	-0.75671770724684	2.51734432907602	-0.12923976805525
C	-0.75095918841743	0.02304131684024	-0.22789546056944
C	-0.74151513923725	-2.47213079272920	-0.15490937039838
C	-0.76766427525615	4.98269276218051	-0.04333007918515
C	-0.73750541127801	-4.93825047568048	-0.09460064522979
C	3.51098990178843	2.52106845803194	-0.07088337073736
C	3.51093297926365	0.03531945735822	-0.10456032322368

C	3.52615072717031	-2.45065164665822	-0.09638449848986
C	-9.31404794563704	2.45435653673359	0.03819252066951
C	-9.31764232194481	-0.00412654622710	0.01676221135315
C	7.75978193091588	0.04785974301406	-0.02897557775999
C	-9.29905667452416	-2.46263860993539	0.01310689418929
C	-3.64196540702678	2.50770362403679	-0.10610028656934
C	-3.63226531563352	0.01419255690261	-0.20190972369400
C	-3.62676337800251	-2.48016464373668	-0.13180440408343
C	-3.64532355334572	4.97348374744200	-0.02387801971195
C	-3.61515271559868	-4.94664225510346	-0.07517145627567
C	0.64843935960390	2.50886305344455	-0.11095032287402
C	0.68467637048263	0.02702211914247	-0.15820829896461
C	0.66357902408405	-2.45535229032794	-0.13652980437893
C	0.62550583078149	4.96958825247332	-0.04090627352125
C	0.65556708950802	-4.91675842169194	-0.09192984769703
C	4.91628573074394	2.49784631878052	-0.05213864346063
C	-12.14201887752631	-0.01311574173121	0.09845157872338
C	4.93454549929031	0.03951716834157	-0.07292936094156
C	4.93131474435215	-2.41911644329724	-0.07730700453418
C	-7.90917346567811	2.48610022891812	0.00093490196675
C	-7.89414356818582	0.00043184073073	-0.03151729744543
C	-7.89401268905179	-2.48548381715764	-0.02451522765220
H	-3.40271193167246	-7.11902097011657	-0.03223031290459
H	-5.55157655423603	-5.88186118837285	-0.02296475059061
H	-7.70362997966850	-4.64477591555303	-0.00784272877514
H	-9.84632330306769	-3.40876657856973	0.02856748691060
H	-11.99363798266294	-2.17183275565364	0.08507722262211
H	-13.23377205833884	-0.01659249910722	0.12871585597195
H	-12.00673702797063	2.14651976616547	0.10702192828904
H	-9.86703255626490	3.39694382884120	0.06328218902404
H	-7.73188863875535	4.64622640963016	0.03986607603065
H	-5.58735265815881	5.89639507188728	0.03789904099441
H	-3.44602904207035	7.14652869878129	0.04190604167768
H	-0.97992397663315	7.15451800462055	0.02245720658476
H	1.16928332842816	5.91762742979816	-0.01217060789370
H	3.32129397778733	4.68016650794551	-0.03446975207474
H	5.46371131659721	3.44387955399349	-0.03553451959786
H	7.61139824170537	2.20666353497491	-0.01853099815606
H	8.85185412484515	0.05107074688351	-0.01321148224786
H	7.62458544543949	-2.11175457316691	-0.04061922498892
H	5.48454834437410	-3.36187279014075	-0.07036566820989
H	3.34958046391163	-4.61110818722520	-0.08217696878086
H	1.20506683819318	-5.86173383087266	-0.07300256169260
H	-0.93659588719858	-7.11188121991403	-0.05161597715335
Mo	-2.18426212000250	0.00987148012517	1.47893227303649

Co-G (mul 6)

C	-7.17553029281959	3.69835186669548	-0.00700177297317
C	-7.17805292362496	1.23780736272756	-0.03405708253188
C	-7.17051960492984	-1.23253209043370	-0.04652255147807
C	-7.15299553033049	-3.69315579506526	-0.04460241262311
C	-2.85472011870318	-6.16707614815017	-0.07148010057258
C	-2.91113858081876	3.72617170554814	-0.03673972500057
C	-2.91309092329906	1.25961014056830	-0.08595976032627
C	-2.90545550991782	-1.22794238605397	-0.09810934559821
C	-2.89230459719384	6.19838106580951	-0.00764856055912

C	-2.88856838682131	-3.69482921681612	-0.07478835491035
C	1.36582887793264	3.74781747537099	-0.05778809953670
C	1.36907496153038	1.26742483343741	-0.08674515197562
C	1.37664539175087	-1.20985413983137	-0.09939196199249
C	1.38841952335948	-3.69038829918883	-0.09605306529124
C	-11.45432876103933	1.20349597970976	0.00867007541041
C	5.63891302522787	1.28680958051593	-0.10360360692948
C	-11.44693983479920	-1.22460036847510	-0.00356114555647
C	5.64636355558318	-1.20317511981059	-0.11629914310204
C	-5.77201045712901	3.72729382467754	-0.02023260525458
C	-5.75871162712151	1.24470359844684	-0.03713061234553
C	-5.75117311706475	-1.23079906730162	-0.04959640780091
C	-5.74929057159881	-3.71343215229621	-0.05820133983744
C	-1.49033106665473	-6.16336812570699	-0.08253553189593
C	-1.49558694152824	3.72928055377032	-0.04107558811164
C	-1.48189762446173	1.26306413270637	-0.04961520817431
C	-1.47427385306819	-1.22316120020219	-0.06209233815542
C	-1.52792079410093	6.20303523695116	-0.01862632379111
C	-1.47300020205853	-3.68934983399551	-0.07917031916635
C	2.77006539240751	3.72901141281003	-0.07030729195708
C	2.78667114240936	1.26895873129737	-0.08237048650483
C	2.79422743618800	-1.20284936596838	-0.09500094225906
C	2.79254955690967	-3.66299958194029	-0.10824347145660
C	-10.02949725938108	1.23795383431243	-0.00278783932431
C	7.06320042722681	1.26035199983633	-0.11161286190000
C	-10.02191947566456	-1.25032121647019	-0.01533045833679
C	7.07040879351317	-1.16803164393446	-0.12399298610263
C	-5.04708257121096	2.48286020861328	-0.04381417973885
C	-5.04728721148175	0.00921016077588	-0.06517822511910
C	-5.03199581586877	-2.46444901784478	-0.06899409914794
C	-5.03438556435636	4.94522811658895	-0.01009448584638
C	-5.00434300918655	-4.92693375614999	-0.06069863012058
C	-0.76889391665383	2.50413332706256	-0.05863984889627
C	-0.75340509625863	0.02228825344338	-0.08388845068310
C	-0.75377535474754	-2.45969068640446	-0.08411556514025
C	-0.76960420002719	4.97586958090197	-0.03560365863792
C	-0.73950388887385	-4.93151809304233	-0.08668030852759
C	3.50270428488838	2.51965800996529	-0.08268074019558
C	3.50470858219939	0.03530854781071	-0.10404787313415
C	3.51779894212249	-2.44916935095118	-0.10810139919743
C	-9.31257439897059	2.45128148429714	-0.00065399972731
C	-9.30986868609787	-0.00397143226252	-0.01551956770645
C	7.75567044259784	0.04822088718061	-0.12169561898903
C	-9.29760997826627	-2.45921475595830	-0.02546015467826
C	-3.63049568141747	2.49379627888624	-0.04343248430963
C	-3.62483284394674	0.01358253936859	-0.07904643193931
C	-3.61534924195519	-2.46681996062321	-0.06854914494124
C	-3.64387857847716	4.96682018791391	-0.01625115777804
C	-3.61371579951367	-4.94006148525198	-0.06716595472287
C	0.64759832373967	2.49986981646738	-0.06181707187449
C	0.66447020287390	0.02658688706374	-0.08269958613694
C	0.66268699404976	-2.44683096553341	-0.08725520634904
C	0.62038725964686	4.96093418827190	-0.04229785852673
C	0.65038914989977	-4.90809421322331	-0.09319922729874
C	4.91476329320864	2.49559697171155	-0.09217520725352
C	-12.13967784476630	-0.01266714122482	0.00789492558629
C	4.92525203784628	0.03966370818886	-0.10671923380369
C	4.92970348456654	-2.41646820356260	-0.11726063886530

C	-7.90031390046292	2.48379286294227	-0.01300849629168
C	-7.88771571094292	0.00039068202275	-0.02480955125389
C	-7.88515743933541	-2.48304561753371	-0.03815798097022
H	-3.40385314443405	-7.11182787898453	-0.06756100891942
H	-5.54865754820387	-5.87490550555518	-0.05527455071225
H	-7.69874709742429	-4.64006562551465	-0.03739105316059
H	-9.84363662252480	-3.40598067073555	-0.02258860716290
H	-11.99123691160086	-2.17151647799398	-0.00367195118907
H	-13.23183492547063	-0.01602975911663	0.01670625310389
H	-12.00430786074852	2.14707594081033	0.01807816847932
H	-9.86435978940329	3.39462254018320	0.01179201615774
H	-7.72709567869076	4.64176222969443	0.00985819458263
H	-5.58440144428124	5.88979515299543	0.00508022085933
H	-3.44713528643856	7.13970399637556	0.00617168283013
H	-0.97870725468688	7.14769769598335	-0.01312496912962
H	1.16510434633939	5.90884525430987	-0.03733959240724
H	3.31494081711416	4.67646243006451	-0.06945304359346
H	5.46110010203751	3.44219561654367	-0.09077983745837
H	7.60786635680577	2.20704923950727	-0.10937249989600
H	8.84784803044737	0.05151536034071	-0.12769541993354
H	7.62068321972861	-2.11146529490151	-0.13139409635760
H	5.48175691372348	-3.35969719393007	-0.12553534668627
H	3.34307468979940	-4.60712945732608	-0.11711335266583
H	1.20083492060266	-5.85268462703695	-0.09808957899555
H	-0.93539972376996	-7.10468513541157	-0.08699681338478
Co	-2.16531681365098	0.00895410623741	1.90007718577609

Fe-G (mul 5)

C	-7.19895954611362	3.69925497636714	-0.01798810768956
C	-7.20277248144722	1.23774613857762	-0.03472935394759
C	-7.19331876829042	-1.23188815522581	-0.04045055684359
C	-7.17294292192819	-3.69356339667531	-0.03694123132707
C	-2.88280229401758	-6.16670008063343	-0.03966388292375
C	-2.93930359231823	3.72602053774300	-0.05088576180165
C	-2.94109965232467	1.25719126054694	-0.10070570087151
C	-2.93073775769939	-1.22086801876624	-0.08695148524789
C	-2.92438609152360	6.20087362228126	0.00572769179738
C	-2.91379267690147	-3.69047331365808	-0.05908294670861
C	1.33066776989406	3.74880418965419	-0.03743337392138
C	1.33325615274796	1.26969075820429	-0.08038938264890
C	1.34168418888351	-1.20663338075553	-0.09881786774874
C	1.35583241845080	-3.68719967331731	-0.07529461918832
C	-11.47724658679741	1.20116611129538	0.00986371719359
C	5.60285621873402	1.29013910368995	-0.04167251617426
C	-11.46857349512737	-1.22880564309125	0.00378851787906
C	5.61109235729089	-1.19968407710745	-0.05497080741426
C	-5.80095998738561	3.72623673975105	-0.02816455414049
C	-5.78680914786013	1.24591640465681	-0.06870143119386
C	-5.77757564592904	-1.22989962767003	-0.06905712804208
C	-5.77428031846700	-3.71037189382217	-0.04836696317199
C	-1.51927162620419	-6.16227353719517	-0.04463100725179
C	-1.53091537636416	3.72930925960680	-0.03581639681235
C	-1.51350914207743	1.26078432092740	-0.09024588225575
C	-1.50351008460339	-1.21764755621747	-0.11660629651877
C	-1.56082996748791	6.20518743056896	0.00484813633253
C	-1.50494885858575	-3.68644860221461	-0.08055785226332

C	2.73037342904771	3.73182476177905	-0.03325794764282
C	2.74986068757455	1.27113241310517	-0.05803660548639
C	2.75800505503743	-1.19894793255295	-0.07054227747315
C	2.75494565235909	-3.66026385894066	-0.07161318155793
C	-10.05696016718478	1.23751623826621	-0.00627791587923
C	7.02488024730691	1.26371957795832	-0.03190209676914
C	-10.04816508669037	-1.25312946682968	-0.01232215626203
C	7.03248734117409	-1.16531045003547	-0.04465879375214
C	-5.07198321133444	2.48171284501966	-0.05073707864413
C	-5.07069635160273	0.01070252200416	-0.07991017976075
C	-5.05406988336260	-2.46104089092468	-0.06710229912533
C	-5.06184152030059	4.94605948364139	-0.01202940605897
C	-5.02793564466229	-4.92526087093112	-0.04180647747080
C	-0.80263263073646	2.49973810037907	-0.07264068976733
C	-0.79040773745964	0.02400883550522	-0.14379159664618
C	-0.78500650128461	-2.45141609363557	-0.09356630458709
C	-0.80316808310464	4.97716594475025	-0.01740843777597
C	-0.76971431850617	-4.92930379464568	-0.06155621462581
C	3.46738361336042	2.52127129955984	-0.04373840830466
C	3.46850006580190	0.03842496430674	-0.07261906878152
C	3.48359334282451	-2.44419338690315	-0.07041399846486
C	-9.33498438636358	2.45251108644537	-0.00783661870819
C	-9.33438189663206	-0.00517138969317	-0.01890556715789
C	7.71706177091095	0.05155473020053	-0.03394036076921
C	-9.31700715084015	-2.46257686971243	-0.02017506986011
C	-3.66029213993114	2.49119523869707	-0.06636935425690
C	-3.65394066954019	0.01613676802231	-0.12489695312623
C	-3.64277355478477	-2.46114648059417	-0.08018258150460
C	-3.67448574404834	4.96825902638312	-0.01761391700503
C	-3.64045694045249	-4.93864194971654	-0.04858567958514
C	0.61152613923364	2.49992132438242	-0.06486606560291
C	0.62549763361474	0.02889768328170	-0.10022305990211
C	0.62827925932671	-2.44221321832440	-0.08730479877189
C	0.58399283085918	4.96379413744567	-0.01706686443426
C	0.61766286605949	-4.90720028842417	-0.06382262643874
C	4.87512329427787	2.49955346623009	-0.03644133884497
C	-12.16031245820864	-0.01630918412493	0.01416203036671
C	4.88910203994522	0.04286163761772	-0.05493936443011
C	4.89069580445965	-2.41394897438218	-0.06229672103048
C	-7.93035636158850	2.48300247483666	-0.02204916379246
C	-7.91275566722060	0.00024673882517	-0.04230177762736
C	-7.91183040757596	-2.48234705276619	-0.03371024407081
H	-3.43279638880757	-7.11082024433642	-0.02813534808019
H	-5.57282151443443	-5.87301655915605	-0.03055564754552
H	-7.71732104457276	-4.64142137813100	-0.02750176903388
H	-9.86178968760424	-3.41010771338633	-0.01500248085379
H	-12.01307882436305	-2.17548986134512	0.00854601868909
H	-13.25255705256254	-0.02014028685070	0.02657624275602
H	-12.02916653290854	2.14354900169278	0.01928248585277
H	-9.88700971203713	3.39574521652881	0.00315370661936
H	-7.74995230299788	4.64321278074684	-0.00354133523539
H	-5.61273687133929	5.89012266711480	0.00887399960845
H	-3.48031069558974	7.14141866107893	0.02502737092130
H	-1.01068866109709	7.14914609828490	0.02191350328300
H	1.12901576208601	5.91133487943743	-0.00006437721236
H	3.27488342139065	4.67944764368712	-0.01802703980982
H	5.42084217498682	3.44645819988689	-0.02558732446476
H	7.56992139854649	2.21013879995185	-0.02212735318708

H	8.80924025633213	0.05499650737776	-0.02624500740461
H	7.58326556889183	-2.10842954972154	-0.04479791176084
H	5.44239104146850	-3.35747264076702	-0.06098406855044
H	3.30627380518112	-4.60407413741913	-0.06659117587352
H	1.16883486244229	-5.85120616672085	-0.05279906666716
H	-0.96305465577946	-7.10275840315419	-0.03565432577835
Fe	-2.19474499353756	0.00822144217346	2.13639717802161
